# Stabilisation of a Strontium Hydride with a Monodentate Carbazolyl Ligand and its Reactivity

**DOI:** 10.1002/anie.202418558

**Published:** 2024-12-10

**Authors:** Lucas Winkler, Alexander Hinz

**Affiliations:** ^1^ Karlsruhe Institute of Technology (KIT) Institute of Inorganic Chemistry (AOC) Engesserstr. 15, Geb. 30.45 Karlsruhe Germany

**Keywords:** Strontium, Hydride, Reduction, Structure, Bulky Ligand

## Abstract

The molecular strontium hydride **2** [(^dtbp^Cbz)SrH(L)]_2_ (L=benzene, toluene) was isolated and stabilized by employing a sterically demanding carbazole ligand (^dtbp^Cbz=1,8‐bis(3,5‐ditertbutylphenyl)‐3,6‐ditertbutylcarbazolyl). Compound **2** was synthesized via phenylsilane metathesis with the corresponding amide (^dtbp^Cbz)SrN(SiMe_3_)_2_ and characterized by ^1^H NMR, XRD and vibrational spectroscopy methods. We further investigated the stoichiometric reactivity of **2** towards carbon monoxide, azobenzene and trimethylsilylacetylene, showing three distinct reactivity pathways: addition, reduction and deprotonation. The reaction of **2** with carbon monoxide yields the ethenediolate complex **4** via addition, while with azobenzene reduction of the N−N double bond and release of hydrogen were observed, affording a heteroleptic strontium complex with a radical azobenzenyl ligand (**5**). The terminal alkyne is deprotonated by the hydride moiety to give the acetylide complex **6**.

## Graphical abstract

A molecular strontium hydride was stabilised by a bulky monodentate ligand and arene co‐ligands. Characteristic vibrations of the Sr_2_H_2_ core were identified and the reactivity patterns were studied with regard to addition, redox and base reactivity.

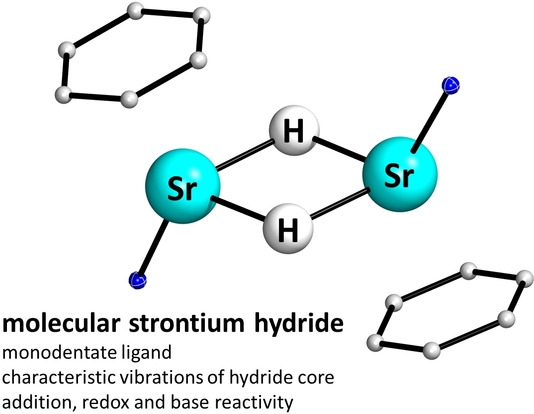

The field of molecular alkaline‐earth‐metal hydrides has received an increasing research interest within the last decades since the first isolation of magnesium and calcium hydride complexes.[^1^, ^2^] Both stoichiometric and catalytic applications of a variety of new different hydrides made these class of compounds a powerful alternative to transition metal‐based hydrides.[^3^, ^4^, ^5^, ^6^, ^7^] However, among the number of reported and structurally authenticated group 2 metal hydrides, the number of hydride complexes is dominated by magnesium and calcium.[^8^, ^9^, ^10^] Molecular hydrides of the heavier analogues like Sr and Ba are significantly less isolated since their stabilization becomes increasingly more difficult. These difficulties arise from Schlenk‐type equilibria in solution resulting in the formation of homoleptic complexes and precipitation of the metal hydrides SrH_2_ or BaH_2_. It was only in 2017 that the first molecular strontium hydride cluster was reported by Harder,^[11]^ and since then the number of well‐defined examples was increased to eight complexes involving polynuclear clusters as well as neutral hydride bridged dimers.[^11^, ^12^, ^13^, ^14^, ^15^, ^16^, ^17^, ^18^] In general, a necessary requirement for the isolation is the coordination of bulky and multidentate ligands which kinetically prevent redistribution reactions, and additional neutral co‐ligands may be required as well. Thus, typical ligand classes employed for Sr hydride stabilization are β‐diketiminates (**A**), cyclopentadienides (**B**), tris(pyrazolyl)borate and amidinate ligands (**C**, Figure 1). The synthetic utility of group 2 hydride complexes is primarily reported for calcium complexes.[^3^, ^6^] Calcium hydrides have been demonstrated to undergo hydrometalation and reduction reactions with various substrates and have been shown to facilitate catalytic hydrogenation and hydrosilylation reactions.[^7^, ^19^, ^20^, ^21^] Strontium hydrides are more scarce and their reactivity is less studied compared to its lighter homologues Mg and Ca with lots of examples for various substrate classes.^[22]^ Harder investigated the *β*‐diketiminato strontium hydride (**A**) with respect to its reactivity towards ethylene and nucleophilic aromatic substitution patterns.^[15]^ Cheng investigated the catalytic activity of cyclopentadienide‐stabilized strontium hydrides (**B**) in the hydrogenation of unactivated alkenes in a comparative study which was reported to increase from Ca to Ba.^[23]^ The Jones group demonstrated that also amidinate ligands can stabilise Sr hydride complexes (**C**)^[13]^ and, very recently, established an extremely bulky BDI ligand for the same purpose.^[24]^ Noteworthily, Sarazin used a bis(imino)carbazolide in barium chemistry, but the corresponding hydride complexes remained elusive.[^25^, ^26^]

**Figure 1:**
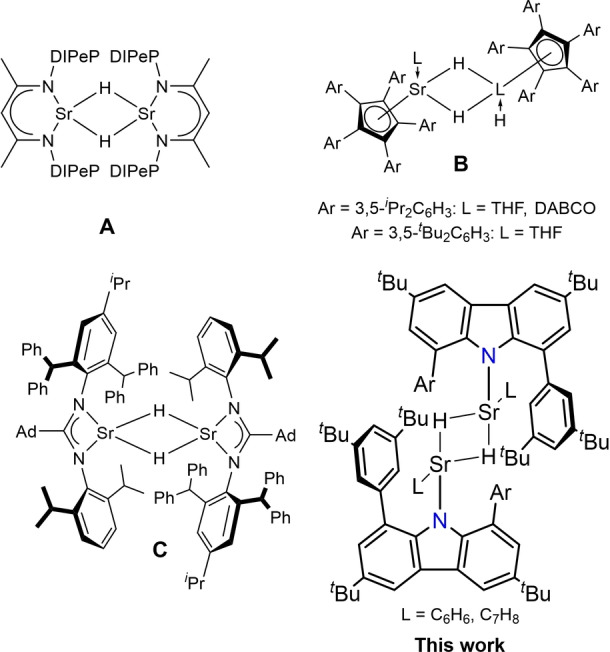
Reported neutral, hydride bridged strontium complexes (Ad=1‐adamantyl, DIPeP=2,6‐(CH(C_2_H_5_)_2_)_2_C_6_H_3_, DABCO=diazabicyclo[2.2.2]octane).

As the utilized ligands for the reported dimeric strontium hydride complexes are bi‐ or multidentate, we were interested in the employment of the bulky monodentate 1,8‐bis(3,5‐ditertbutylphenyl)‐3,6‐ditertbutylcarbazolyl ligand (^dtbp^Cbz). Our earlier contributions include stabilisation of low‐coordinated environments of p‐block elements,[^27^, ^28^] barium silanide as well as Ca and Yb hydride complexes.[^29^, ^30^] Here, we describe the isolation of a carbazolyl strontium hydride and its reactivity towards selected unsaturated substrates.

The synthesis of a molecular carbazolyl strontium hydride was pursued via σ‐bond metathesis of an amido precursor with phenyl silane (Scheme 1). Hence, transamination of ^dtbp^Cbz‐H with the bis(trimethylsilyl)amido compound Sr[N(SiMe_3_)_2_]_2_ was carried out in toluene, yielding the carbazolyl amido complex (^dtbp^Cbz)SrN(SiMe_3_)_2_ **1** within hours at ambient temperature. After evaporation of the solvent and concentrating a *n*‐hexane or benzene solution, **1** could be obtained as crystalline yellow material which allowed investigation of the solid‐state structure (see Figure S5). Depending on the solvent used for crystallization, the molecular structure shows a monomeric (benzene, **1 a**) or a dimeric (*n*‐hexane, **1 b**) carbazolyl strontium amide. Apparently, even the relatively weak donor solvent benzene is capable of breaking up the dimeric structure of **1**. A similar observation was made for the analogous barium amide,^[29]^ but for the corresponding calcium compound, only monomeric complexes were observed.^[31]^ In the monomer, the Sr ion is located in near carbazole plane and and shows contacts to both flanking arenes (3.07–3.16 Å). The metal is bent out of the central pyrrole plane of the carbazole by 12.8°. Additionally, the Sr−N2 bond of 2.4724(17) Å is shorter than the Sr−N1 bond of 2.402(2) Å. In the dimeric carbazolyl strontium amide **1 b**, Sr‐N_amide_ bonds are elongated (2.560(7), 2.621(9) Å) and the Sr atom is displaced by 2.17 Å from the carbazole plane. The ^1^H and ^13^C{^1^H} NMR spectra of **1** in C_6_D_6_ solution show only one set of signals, indicating a symmetric coordination on the NMR time scale.

**Scheme 1:**
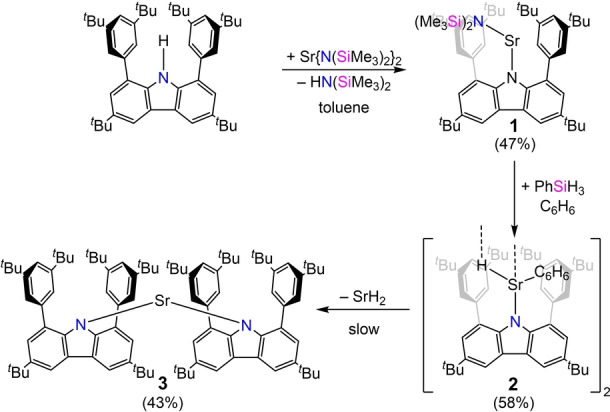
Synthesis and decomposition of carbazolyl strontium hydride **2**, isolated yields given in brackets.

To prepare the corresponding carbazolyl hydride complex, the amido precursor **1** was dissolved in benzene or toluene and treated with an excess of phenylsilane (PhSiH_3_) at room temperature without stirring of the reaction mixture. Within one night, yellow crystals deposited and, depending on the chosen solvent, the dimeric hydrides [(^dtbp^Cbz)SrH⋅C_6_H_6_]_2_ (**2 a**) and [(^dtbp^Cbz)SrH⋅C_7_H_8_]_2_ (**2 b**) could be obtained. The solid‐state structures were determined by X‐ray diffraction analysis. Both solvates **2 a** and **2 b** crystallize as hydride‐bridged dimers (Figure 2, for **2 b** see Figure S11).

**Figure 2:**
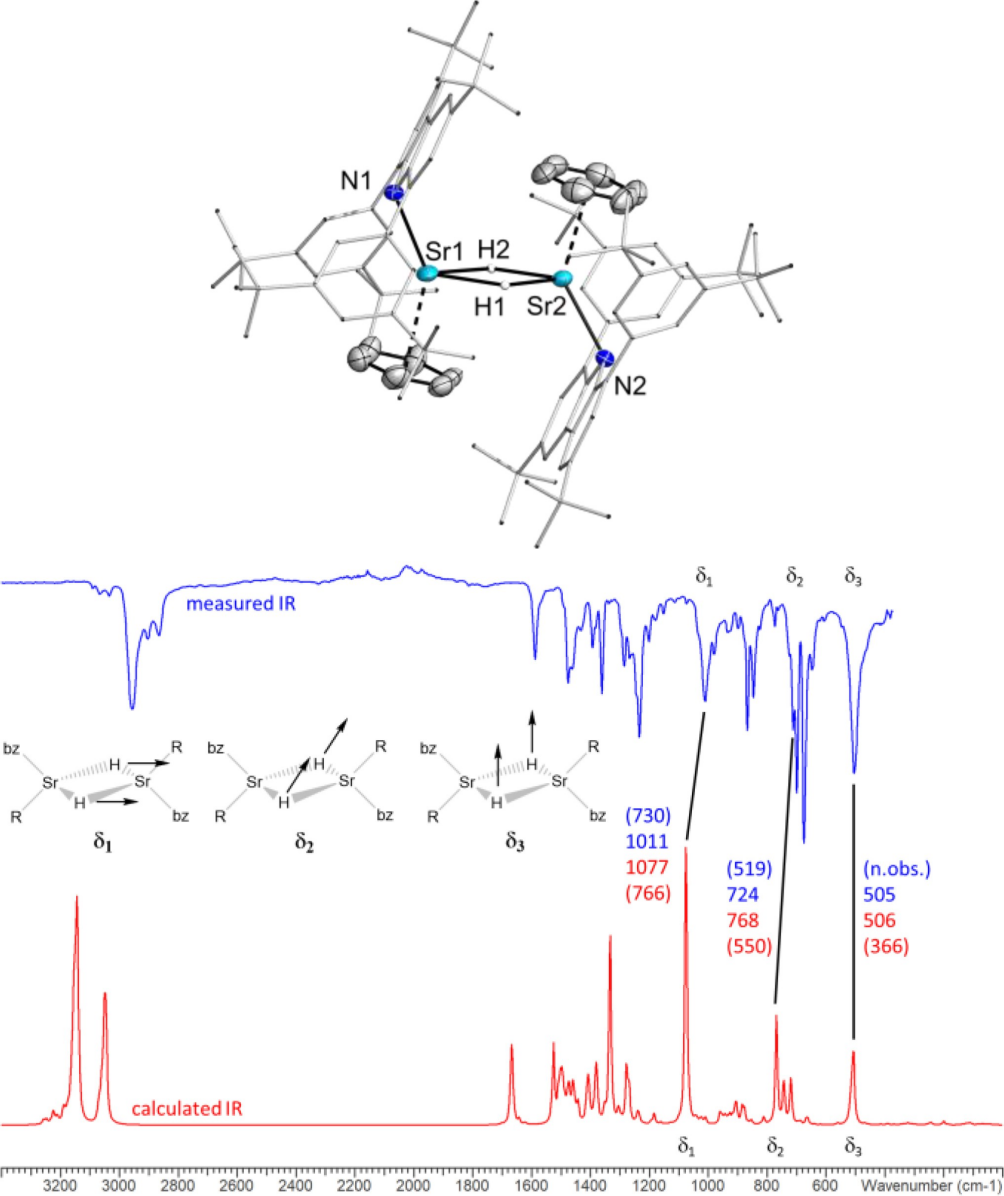
Top: Molecular structure of **2 a**. Thermal ellipsoids with 50 % probability at 150 K. Bottom: Selected vibrational modes of the Sr_2_H_2_ core (observed data in blue, calculated data in red, values for the deuteride in brackets).

The average Sr−H distances of 2.32 Å in **2 a** and 2.35 Å in **2 b** are within the range of other neutral dimeric strontium hydride complexes (2.26(5)–2.43(3) Å).[^13^, ^15^, ^16^] Each of the Sr atoms is additionally coordinated by one arene solvent molecule. The Sr−N distances (2.513(2) and 2.4431(19) Å for **2 a**) are slightly shorter than for known strontium hydride complexes, stabilized by monoanionic nitrogen‐based ligands like amidinates or ß‐diketiminates.[^13^, ^15^] Additionally, in both complexes, the Sr atoms show contacts towards the carbon atoms of the carbazole backbone or the *ipso* carbon of one flanking arene. The solubility of the strontium hydride **2** is poor in aliphatic and aromatic solvents. However, NMR characterization was possible in a saturated C_6_D_6_ solution. The ^1^H NMR spectrum shows one signal set and a hydride resonance at 3.42 ppm (see Figure S6). This chemical shift is at higher field than the reported neutral dimeric strontium hydrides (δ=4.91–6.64 ppm),[^13^, ^15^, ^16^] but compares reasonably well to the computed value of 2.40 ppm (Gaussian16/PBE0‐GD3BJ/Def2‐SVP).^[32]^ The shielding of the hydride resonance is likely due to interations with adjacent π systems. In silico, various parts of the molecule were removed to elucidate the origin of the unusual highfield shift. Surprisingly, the impact of the flanking arenes on the ^1^H NMR shift is considerably smaller than that of the coordinated benzene molecule (Δδ 0.73 vs 1.89 ppm). In addition to NMR spectroscopy, compound **2** was analyzed by vibrational spectroscopy. The IR spectrum shows three characteristic vibrations (Figure 3) for [(^dtbp^Cbz)SrH⋅C_6_H_6_]_2_ (**2 a**). Based on DFT calculations, the three vibrational modes δ_1_ (1011 cm^−1^), δ_2_ (724 cm^−1^) and δ_3_ (505 cm^−1^) were identified and exclusively involve the Sr_2_H_2_ core of the molecule. Two further vibrational modes that involve the Sr_2_H_2_ moiety were found in the DFT model (see Supporting Information 4.1). However, they could not be observed experimentally. In the Raman spectra, no clear assignment was possible.

**Figure 3:**
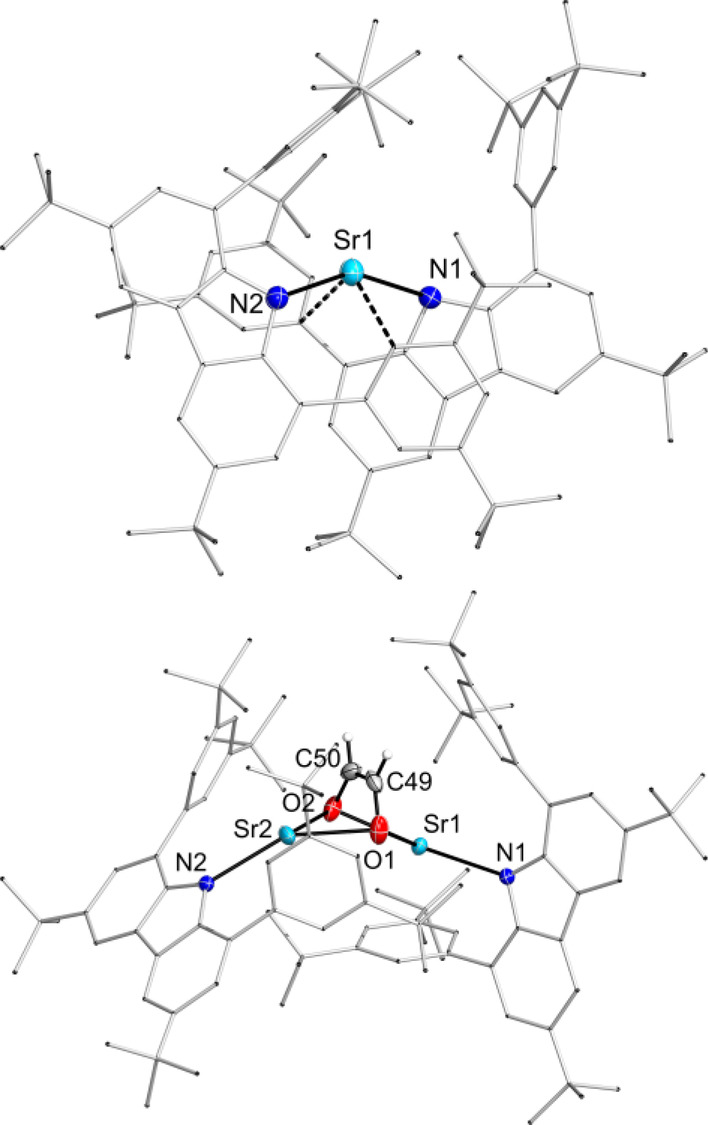
Molecular structure of **3** and **4**. Thermal ellipsoids with 50 % probability at 200 K (**3**)/150 K (**4**).

The hydride complex is stable in the solid state for weeks at ambient temperature, but in solution with aromatic solvents decomposition is observed within days (see Figure S7). In aliphatic solvents the decomposition rate is diminished due to the lower solubility. From a sample of **2** in *n*‐hexane, the homoleptic bis(carbazolyl) complex (^dtbp^Cbz)_2_Sr **3** was isolated after one week. Unambiguous identification was possible, as single crystals of compound **3** were obtained from *n*‐hexane solution. The molecular structure is shown in Figure 3.

Due to the high steric bulk of the two carbazoles, the Sr−N distances are elongated (Sr1−N1 2.558(3) Å and Sr1−N2 2.499(3) Å) in comparison to the hydride complex **2**. To saturate its coordination sphere, the metal atom exhibits several contacts to different carbon atoms of the carbazole backbones, arene moieties or *tert*‐butyl groups (2.89–3.31 Å). Another consequence of the proximity of both carbazoles is twisting of the carbazole planes with a dihedral angle of 63.0°. The N1−Sr−N2 angle 153.03(8) is significantly larger compared to the amide **1** in the monomer.

To better understand the chemistry of the strontium hydride complexes, we were interested in the reactivity of compound **2** towards selected unsaturated molecules, carbon monoxide, azobenzene and trimethylsilylacetylene (Scheme 2).

**Scheme 2:**
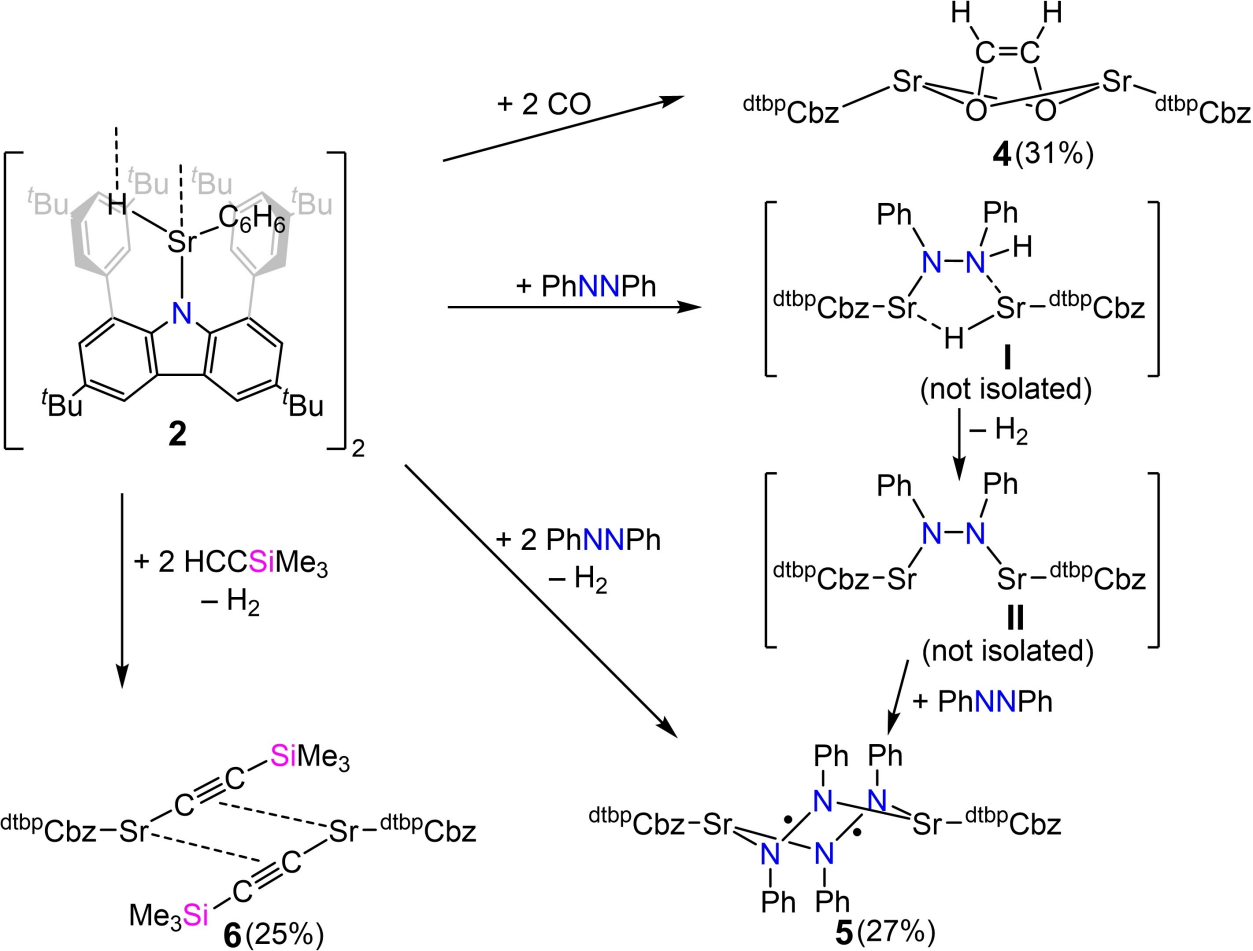
Reactivity of carbazolyl strontium hydride **2** (isolated yields given in brackets).

In an initial reaction performed on NMR scale, a suspension of the hydride **2** in C_6_D_6_ was exposed to a carbon monoxide atmosphere and after mixing a pronounced colour change to orange was observed. The NMR experiment afterwards confirmed the consumption of the hydride and showed one major product. Reaction with carbon monoxide led to the formation of a dimeric carbazolyl strontium *cis*‐ethenediolate complex **4** (Figure 5).

Concentrating a toluene solution led to formation of single crystals which allowed elucidation of the solid‐state structure. Both Sr^2+^ atoms exhibit a nearly similar coordination sphere. The strontium ion is slightly bent out of the plane of the central pyrrole ring (Sr1 17.19° and Sr2 16.49°). There are secondary Sr‐arene contacts (six for Sr1 and three for Sr2) in the range of 3.073(2) to 3.416(3) Å. The bridging *cis*‐ethenediolate ligand is bound symmetrically to each metal center which is similar to the bond pattern of [(Tp^Ad,*i*Pr^)Ba(*cis*‐OCH=CHO)Ba(Tp^Ad,*i*Pr^)].^[23]^ As a result of the coordination modes of the *cis*‐ethenediolate ligand and the carbazoles to each strontium ion, both carbazoles are twisted with a torsion angle of 97.3°. ^1^H and ^13^C{^1^H} NMR analysis of compound **4** was performed to identify the ethenediolate moiety with resonances at 3.05 and 77.17 ppm. Keeping a C_6_D_6_ solution of **4** at room temperature for some days indicated slow decomposition by formation of a precipitate. NMR analysis revealed that the *cis*‐ethenediolate complex slowly decomposes into the homoleptic compound **3**. The formation of ethenediolate is the commonly observed reaction when alkaline earth metal hydrides are treated with CO and **2** is no exception here.^[33]^

Reactions of molecular hydrides with azobenzenes are reported in the literature with different element‐hydrogen bonds. The usual reaction pattern is the hydrometallation of the N−N double bond.[^34^, ^35^, ^36^] In contrast to that, the strontium hydride **2** was found to act as a reducing agent instead of a hydride source like it is reported for C−C double bonds.[^6^, ^15^] An equimolar reaction of SrH moiety of **2** with azobenzene, leading to the formation of a monoanionic azobenzenyl ligand. Workup of the reaction yielded dark green crystals of the carbazolyl strontium azobenzenyl complex **5** (Figure 4).

**Figure 4:**
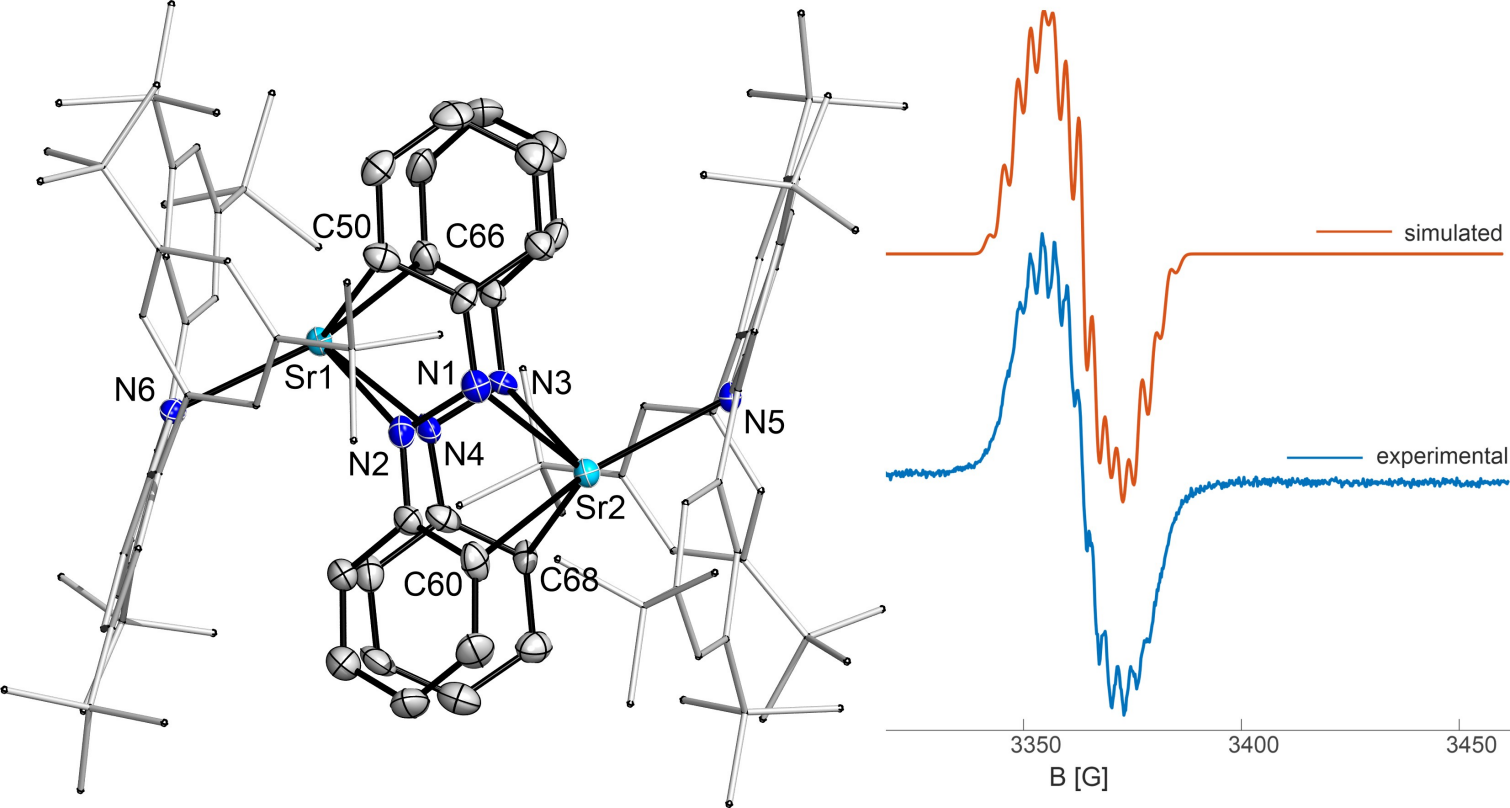
Top: Molecular structure of **5**. Thermal ellipsoids with 50 % probability at 100 K. Bottom: CW‐EPR spectrum of **5** in toluene at ambient temperature (simulation parameters: g 2.0023, A(N1) 15.0, A(N2) 14.9, A(4 *o*‐H) 10.0, A(2 p‐H) 7.0 MHz).

Compound **5** crystallizes as bridged dimer in the monoclinic space group *Cc*. The average N−N bond length of 1.36 Å is comparable to a reported magnesium azobenzenyl complex and lies within the range of a N−N single (1.45 Å) and N−N double bond (1.25 Å).^[37]^ Both Sr^2+^ ions show coordination to each azobenzenyl moiety in a η^4^ fashion with two shorter and two longer contacts between Sr and C or N, respectively. Additionally, the Sr‐N_Carb_ bonds are elongated in comparison to the hydride **2**. NMR in situ experiments corroborate the formation of **5** via two intermediates **I** and **II** which can be tentatively assigned as featuring a diphenylhydrazido monoanionic (**I**) and a diphenylhydrazido dianionic ligand (**II**, see also Supporting Information section 2.5). Unexpectedly, both NMR and EPR spectra of solutions of **5** are observable. The ^1^H and ^13^C{^1^H} NMR spectra of compound **5** display typical resonances for a diamagnetic compound.^[38]^ In addition to NMR spectroscopy, the azobenzenyl complex **5** was studied with EPR spectroscopy as well. The EPR spectrum of **5** in toluene solution at ambient temperature shows a signal at a g value of 2.0023 with hyperfine splitting due to interaction of the electron with two distinct N atoms, four o‐CH and two p‐CH protons, typical for an organoradical. A possible explanation for both NMR and EPR activity is that the major fraction of **5** is present as diamagnetic dimer, allowing regular NMR spectra to be detected, while only a minor fraction occurs as either dimer in its triplet state or as dublet monomers, allowing EPR detection. Notably, the depicted simulation was achieved with a dublet monomer spin system model. The UV/Vis spectrum shows absorption in the full measured range, with broad maxima at 572 and 731 nm in the visible region. However, this allows no further distinction, as the dimeric singlet **5** is predicted to exhibit strong transitions at 762 and 738 nm (Gaussian16/PBE0‐GD3BJ/Def2‐SVP),^[32]^ while both monomeric doublet and dimeric triplet **5** show strong transitions at 561 nm or 551 and 571 nm, respectively, and either no or very weak transitions in the longer wavelength range.

Lastly, the reactivity of strontium hydride **2** was tested towards a terminal alkyne. A facile reaction was observed, as the acidic terminal proton of trimethylsilylacetylene (Me_3_SiC≡CH) is readily depronotonated in toluene at room temperature to give a dimeric acetylide complex [(^dtbp^Cbz)Sr(CCSiMe_3_)]_2_ **6** (Figure 5).

**Figure 5:**
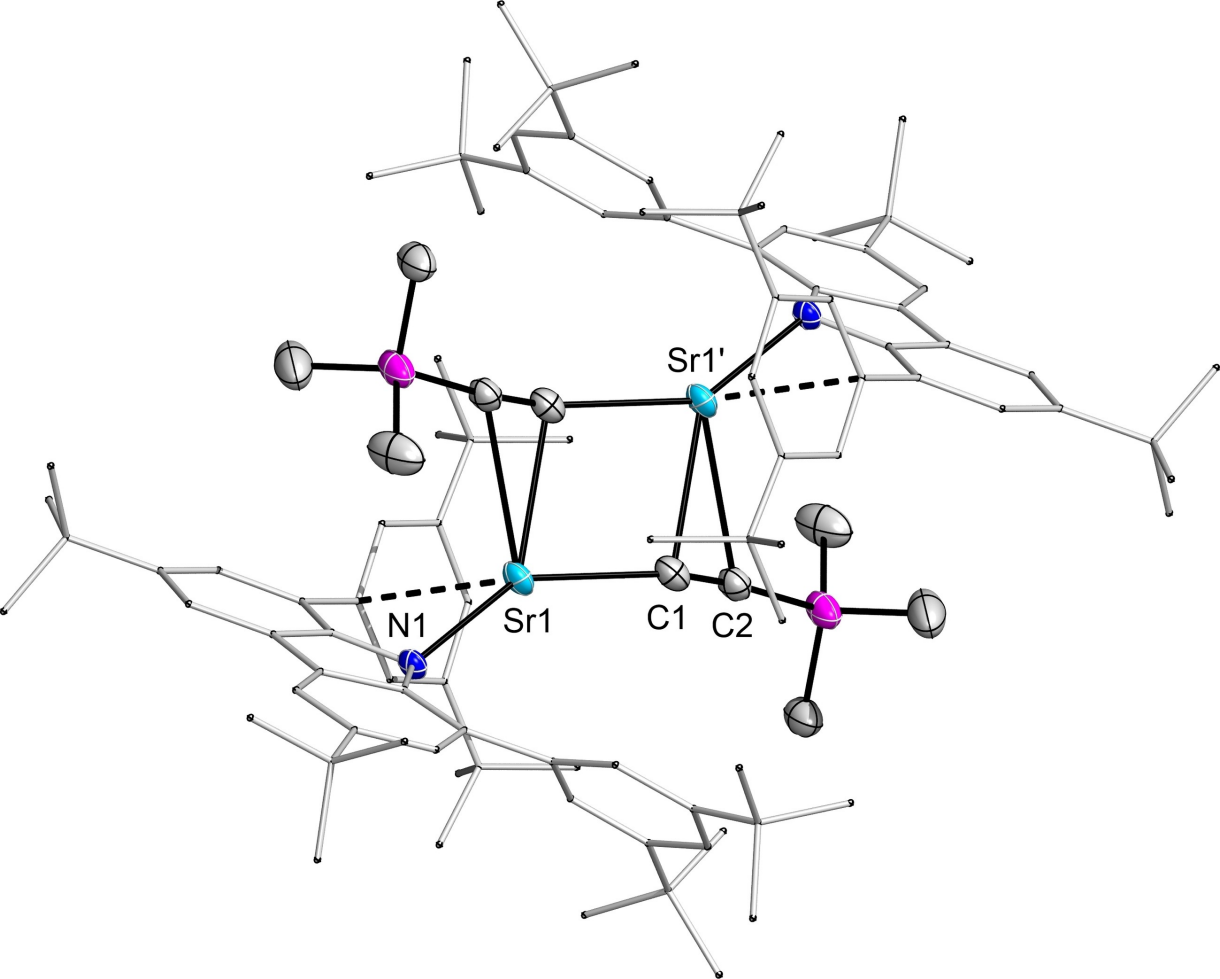
Molecular structure of **6**. Thermal ellipsoids with 50 % probability at 100 K.

Suitable crystals for XRD diffraction were obtained from a saturated toluene solution. The bond length of 1.202(5) Å within the acetylid (C1−C2) is comparable to reported calcium acetylide complexes.[^39^, ^40^] The four‐membered Sr_2_C_2_ core is essentially planar. The metal atom is bent out of the plane of the central pyrrole ring by 30.8° and additionally coordinated by contacts from an arene moiety and the second atom of the acetylide moiety (Sr1−C2 3.026 Å).

The ^13^C{^1^H} NMR chemical shifts for the metal‐bound acetylide carbons are found at a characteristic low‐field resonance with 174.23 ppm (C_α_) and at 130.50 (C_β_) which both are in comparable regions with regard to calcium acetylides.[^40^, ^41^]

We report the synthesis of a dimeric strontium hydrido complex with a monodentate carbazolyl co‐ligand. The hydride complex features a ^1^H NMR resonance at 3.42 ppm and shows characteristic vibrational modes of the Sr_2_H_2_ core. Its reactivity with carbon monoxide, azobenzene and a substituted acetylene was investigated, displaying addition, reduction and deprotonation reactivity. The reaction with carbon monoxide leads to the expected ethenediolate product. In the reaction with azobenzene, reduction to a radical anion azobenzene ligand was observed while H_2_ was eliminated. A terminal alkyne was deprotonated to form an acetylide complex. The limit of stabilization appears to be nearly reached, as the hydride complex is isolable, but both the hydride and the ethenediolate derivative slowly decompose in solution.

## Supporting Information

The authors have cited additional references within the Supporting Information (Ref. [42–49]).

Deposition Number(s) 2145433 (for **1 a**), 2145434 (for **1 b**), 2145440 (for **2 a**), 2145439 (for **2 b**), 2145441 (for **3**), 2385469 (for **4**), 2385470 (for **5**), 2385471 (for **6**), 2403487 (for **II**) contain the supplementary crystallographic data for this paper. These data are provided free of charge by the joint Cambridge Crystallographic Data Centre and Fachinformationszentrum Karlsruhe

## Conflict of Interests

The authors declare no conflict of interest.

## Supporting information

As a service to our authors and readers, this journal provides supporting information supplied by the authors. Such materials are peer reviewed and may be re‐organized for online delivery, but are not copy‐edited or typeset. Technical support issues arising from supporting information (other than missing files) should be addressed to the authors.

Supporting Information

## Data Availability

The data that support the findings of this study are available in the supplementary material of this article.
