# Supplementary material for: Stabilisation of a Strontium Hydride with a Monodentate Carbazolyl Ligand and its Reactivity
Source: Angew Chem Int Ed Engl. 2024 Dec 10;64(5):e202418558. doi: 10.1002/anie.202418558 (PMC11773309; doi:10.1002/anie.202418558)
Supplement: Supplementary file 1 — Supporting Information [file ANIE-64-e202418558-s001.pdf]

Angewandte Chemie
Eine Zeitschrift der Gesellschaft Deutscher Chemiker

Supporting Information

Stabilisation of a Strontium Hydride with a Monodentate Carbazolyl Ligand and its Reactivity

*L. Winkler, A. Hinz**

1 General Considerations

All experiments were conducted in dry glassware under an inert argon atmosphere by applying standard Schlenk techniques unless otherwise reported.

Toluene (Roth, 99%), *n*-hexane (Roth, 98%) and benzene (Honeywell) were dried over sodium and distilled prior to use. C_6D_6 (Eurisotop, 99.5%) was dried over molecular sieves and distilled prior to use. All dry solvents were stored under argon in gas-tight ampoules over 3 Å molecular sieves. Phenyl silane (97%, Sigma-Aldrich), carbon monoxide (Air Liquide, 98%) and azobenzene(Sigma-Aldrich) were used as received. Trimethysilylacetylene (abcr, 98%) was dried over molecular sieves, distilled and degassed prior to use.

Starting materials were prepared according to literature protocols: ^{dtbp}CbzH,[1] Sr[N(SiMe$_3$)$_2$]$_2$.[2]

NMR spectra were acquired on a Bruker Avance 400 MHz or Avance 300 MHz spectrometer. Reported chemical shifts are referenced to the ^{1}H and ^{13}C NMR resonances of the deuterated solvent.[3] Coupling constants *J* are given in Hertz as positive values regardless of their real individual sign. ^{1}H, ^{13}C{^{1}H} NMR and ^{29}Si NMR spectra were obtained at 400, 100.7 MHz and 79.5 MHz, respectively. The multiplicity of the signals was indicated by the abbreviations br (broad), m (multiplet), s (singlet), d (doublet), t (triplet), etc.

Continous wave EPR spectroscopy (CW-EPR) was performed at X-band on a Bruker EMXplus spectrometer. The field was calibrated by using 2,2-diphenyl-1-picrylhydrazyl with a *g* value of 2.0036.[4] CW EPR simulations were carried out by using the EasySpin implementation in MatLab.

IR spectra were recorded on a Bruker Alpha FT-IR spectrometer using the ATR technique (attenuated total reflection) on powdered samples, and the data are quoted in wavenumbers (cm^{-1}). The signals were classified according to their intensity into very strong (vs), strong (s), medium (m), weak (w) and very weak (vw).

Single crystals were mounted in perfluoropolyalkyl ether oil on a cryo loop and then brought into the cold nitrogen stream of a low-temperature device (Oxford Cryosystems Cryostream unit) so that the oil solidified. Diffraction data were collected using a Stoe IPDS II diffractometer and graphite-monochromated Mo-Kα (0.71073 Å) or Ga-Kα (1.34143 Å) radiation. The structures were solved by direct methods with SHELXS[5] intrinsic phasing with SHELXT[6] followed by full-matrix least-squares refinement using SHELXL-2014/7[7] and the ShelXle GUI.[8] All non-hydrogen atoms were refined anisotropically. The contribution of the hydrogen atoms, in their calculated positions, was included in the refinement using a riding model.

UV/VIS spectra were recorded on a UV/VIS-Excellence UV7 spectrometer of the company Mettler Toledo. The absorption was measured between 250 and 900 nm.

2 Syntheses

2.1 ^{dtbp}CbzSrN(SiMe$_3$)$_2$ (**1**)

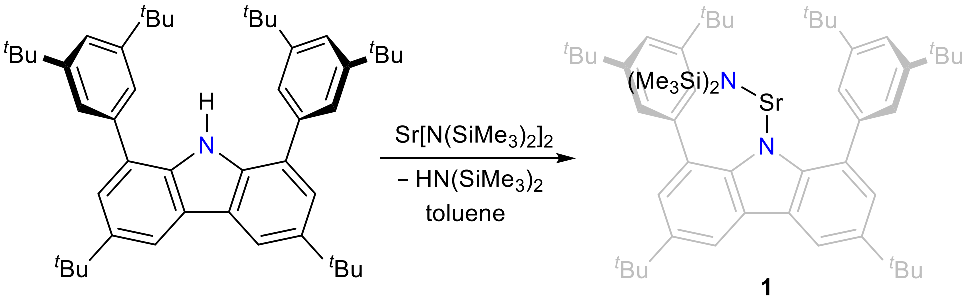

1

As solids, ^{dtbp}CbzH (663 mg, 1.011 mmol) and Sr[N(SiMe$_3$)$_2$]$_2$ (408 mg, 1.000 mmol) were combined. To the mixture, 13 mL of toluene were added via syringe. The resulting solution quickly turned dark yellow and was stirred overnight at ambient temperature. Then, the solvent was evaporated and the crude product was treated with 22 mL *n*-hexane, filtered and concentrated until incipient crystallization. Within one night, suitable crystals were obtained. The crystals were washed twice with 2 mL *n*-hexane and dried in vacuo. Crystalline yield: 428 mg, 0.474 mmol, 47%. The characterisation data were obtained from the crystalline material.

^{1}H NMR (400 MHz, C_6D_6): δ (ppm) = -0.20 (s, 18 H, Si(C*H*$_3$)$_3$), 1.28 (s, 36 H, Ar-C(C*H*$_3$)$_3$), 1.58 (s, 18 H, Carb-C(C*H*$_3$)$_3$), 7.57 (t, 2 H, $^4J_{HH}$ = 1.9 Hz, *p-CH*), 7.69 (d, 2 H, $^4J_{HH}$ = 1.9 Hz, C^{2,7}*H*), 7.94 (d, 4 H, $^4J_{HH}$ = 1.9 Hz, *o*-C*H*), 8.65 (d, 2 H, $^4J_{HH}$ = 2.0 Hz, C^{4,5}*H*).

^{13}C{^{1}H} NMR (100.7 MHz, C_6D_6): δ (ppm) = 5.43 (s, Si(*C*H$_3$)$_3$), 31.85 (s, Ar-C(*C*H$_3$)$_3$), 32.63 (s, Carb-C(*C*H$_3$)$_3$), 34.91 (s, Carb-*C*(CH$_3$)$_3$), 35.44 (s, Ar-*C*(CH$_3$)$_3$), 116.97 (s, *C*^{4,5}), 122.79 (s, *p*-*C*H), 122.97 (s, *C*^{2,7}), 124.09 (s, *o*-*C*H), 126.43 (s, *C*^{4a,4b}), 126.72 (s, *C*^{1,8}), 138.80 (s, *C*^{3,6}), 146.63 (s, *i*-*C*), 148.90 (s, *C*^{8a,9a}), 153.98 (s, *m*-*C*).

^{29}Si NMR (79.5 MHz, C_6D_6): δ (ppm) = -17.0 (s, -*Si*(CH$_3$)$_3$).

IR (ATR): ũ (cm^{-1}) = 2954 (s), 2867 (vw), 2163 (vw), 1588 (w), 1462 (w), 1392 (w), 1362 (m), 1286 (w), 1271 (w), 1236 (vs), 1202 (vw), 1152 (vw), 1058 (vs), 968 (vw), 928 (w), 866 (s), 851 (m), 817 (vs), 759 (w), 722 (m), 702 (vw), 665 (w), 647 (w), 606 (vw), 584 (w), 498 (vw), 456 (vw), 431 (vw), 405 (vw), 544 (vw), 504 (vw), 467 (vw), 426 (vw), 411 (vw), 390 (w).

EA found (calc. for $C_{54}H_{82}N_2Si_2Sr$): C 71.16 (71.82), H 8.81 (9.15), N 3.43 (3.10).

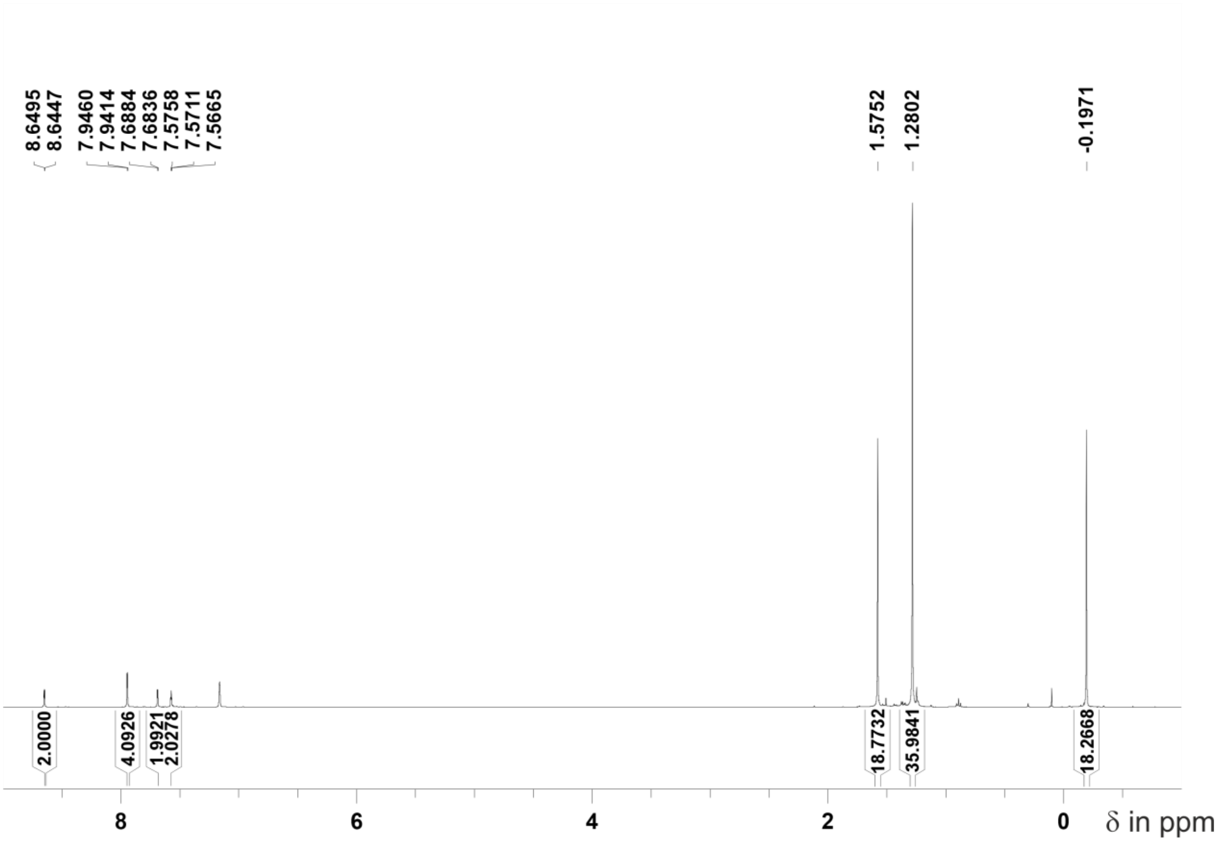

Figure S1: ^{1}H NMR spectrum of **1**.

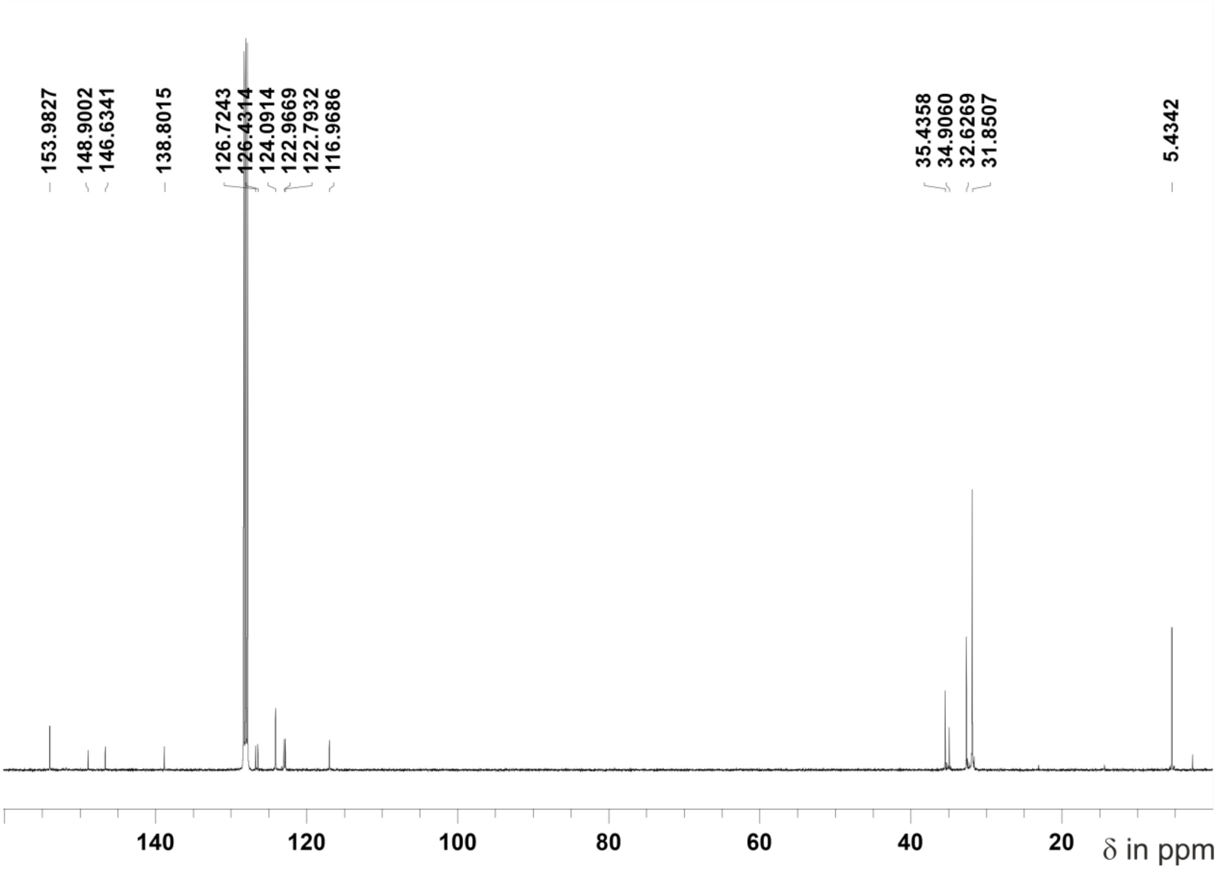

Figure S2: $^{13}C\{^{1}H\}$ NMR spectrum of **1**.

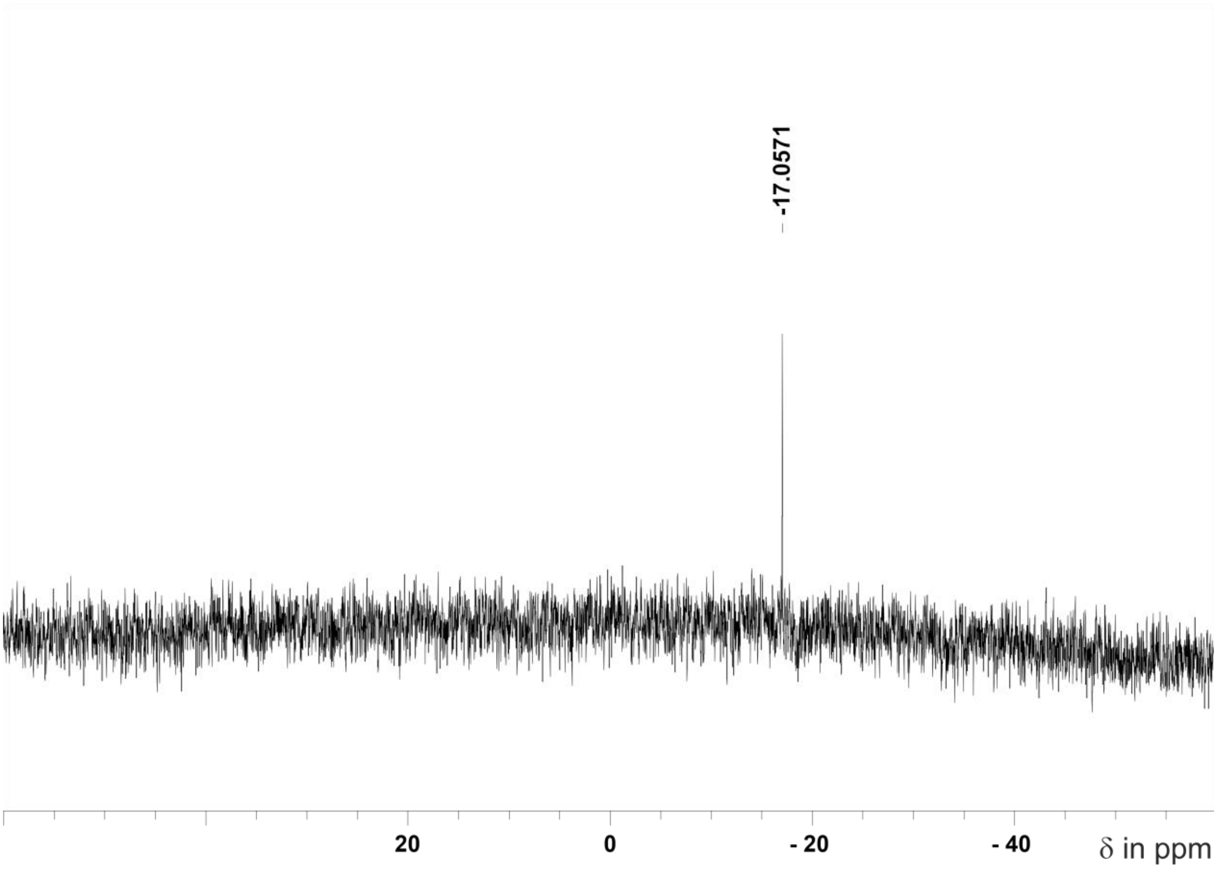

Figure S3: ^{29}Si NMR spectrum of **1**.

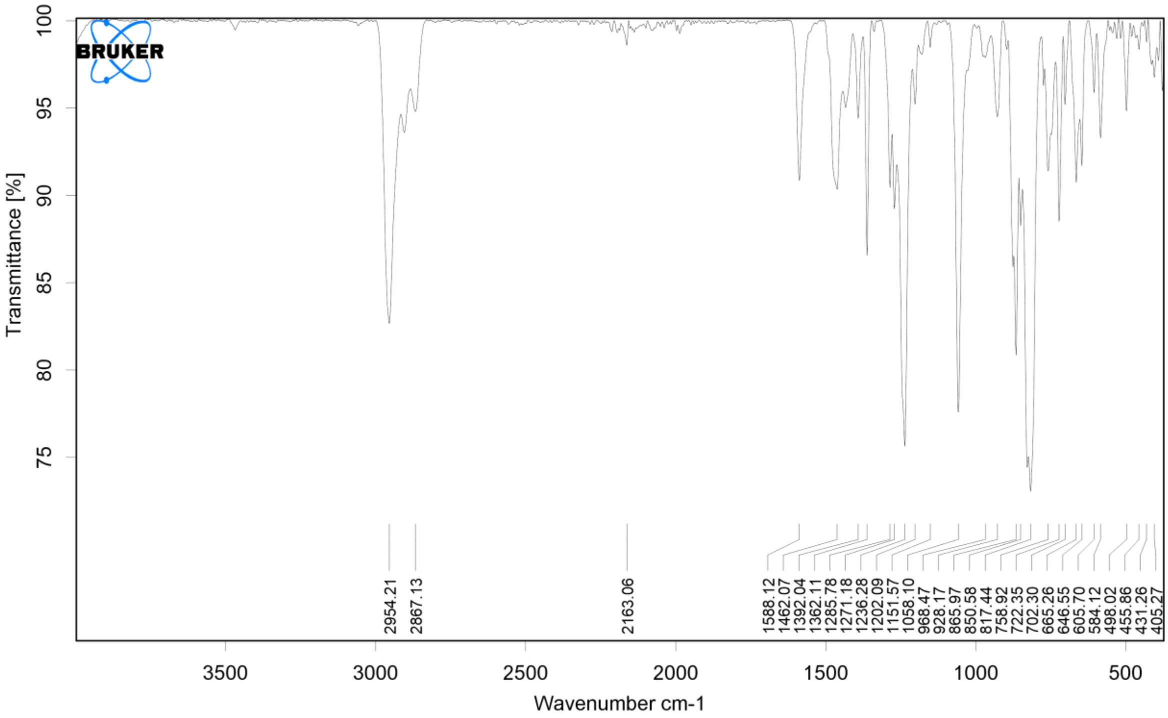

Figure S4: IR spectrum of **1**.

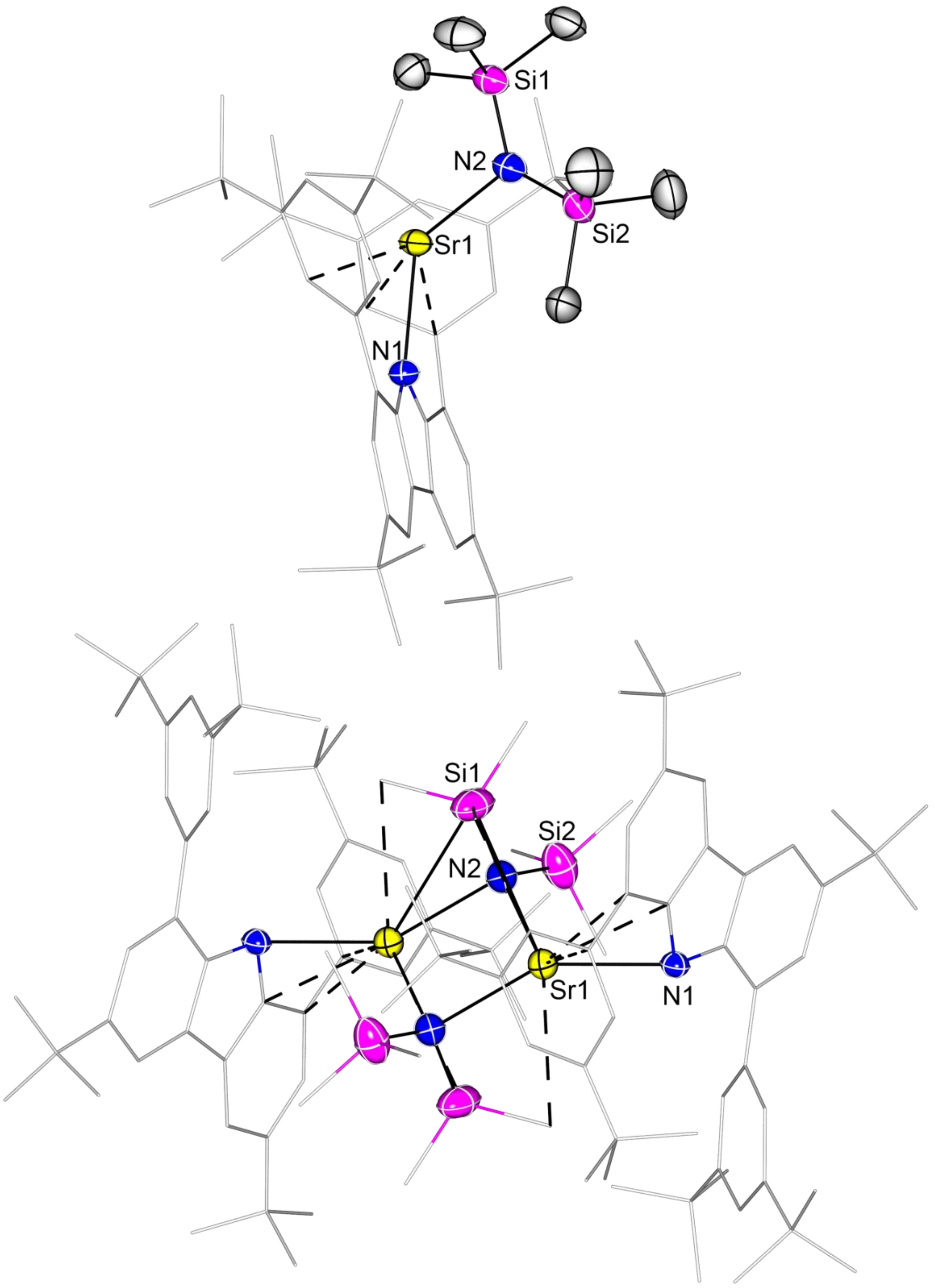

Figure S5: Molecular structures of **1a** (top) and **1b** (bottom). Thermal ellipsoids with 50% probability at 200 K (**1a**) and 150 K (**1b**).

2.2 [^{dtbp}CbzSrH·arene]$_2$ (2)

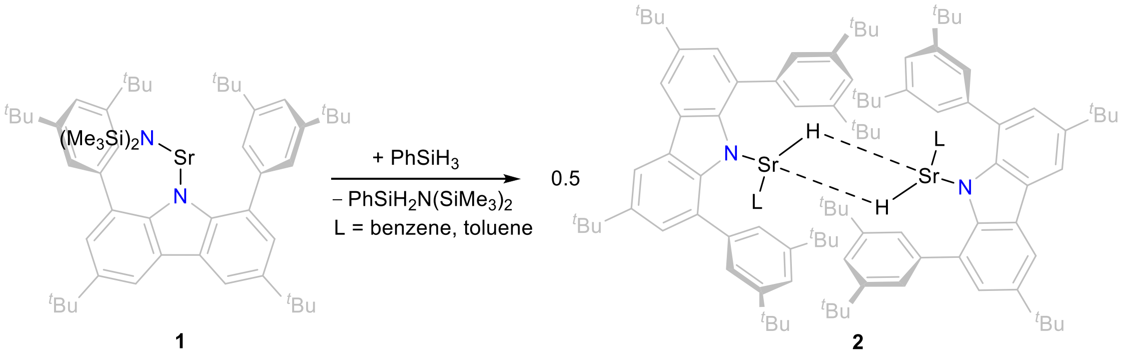

The amide **1** (248 mg, 0.275 mmol) was dissolved in 2.0 mL of benzene. To that solution, phenylsilane (64 µL, 56.1 mg, 0,518 mmol, 1.88 eq.) was added via microsyringe. The mixture was shaken and then left undisturbed overnight. The hydride **2** crystallized within one night. Afterwards, the supernatant was removed via syringe, the crystals were washed multiple times with *n*-hexane and briefly dried. Crystalline yield: 119 mg, 0.080 mmol, 58%. The characterisation data were obtained from the crystalline material.

In the same reaction, toluene can be used as solvent, and RSrH·toluene is obtained.

^{1}H NMR (400 MHz, C_6D_6): δ (ppm) = 1.47 (br, 36 H, Ar-^{t}Bu), 1.55 (s, 18 H, Carb-^{t}Bu), 3.42 (s, 1 H, Sr*H*), 7.49 (d, 2 H, $^4J_{HH}$ = 2.0 Hz, C^{2,7}*H*), 7.55 (t, 2 H, $^4J_{HH}$ = 1.8 Hz, *p*-C*H*), 7.89 (br, 2 H, *o*-C*H*), 8.51 (d, 2 H, $^4J_{HH}$ = 2.0 Hz, C^{4,5}*H*).

^{1}H NMR (400 MHz, THF-D_8): δ (ppm) = 1.31 (s, 36 H, Ar-^{t}Bu), 1.41 (s, 18 H, Carb-^{t}Bu), 7.22 (br, 2 H, *p*-C*H*), 7.30 (s, 4 H, *o*-C*H*), 7.91 (br, 4 H, C$^{2,7/4,5}$*H*), hydride Sr*H* could not be assigned.

Due to the poor solubility of compound **2**, only ^{1}H NMR solution characterization was performed.

IR (ATR): ũ (cm^{-1}) = 2953 (m), 2903 (vw), 2866 (vw), 1589 (w), 1475 (w), 1392 (w), 1361 (m), 1286 (w), 1269 (w), 1234 (s), 1201 (vw), 1181 (vw), 1151 (vw), 1012 (m), 980 (vw), 933 (vw), 899 (vw), 866 (m), 848 (w), 775 (vw), 726 (w), 713 (s), 702 (vs), 644 (vw), 506 (s), 408 (vw), 385 (vw).

Raman (cm^{-1}): 71 (100), 104 (76), 147 (39), 175 (12), 210 (21), 239 (14), 368 (7), 519 (7), 550 (13), 609 (9), 700 (7), 783 (11), 825 (18), 852 (11), 867 (8), 885 (9), 897 (10), 924 (18), 995 (57), 1026 (7), 1099 (9), 1153 (9), 1178 (13), 1203 (16), 1255 (22), 1267 (17), 1288 (12), 1319 (62), 1336 (24), 1365 (9), 1398 (13), 1444 (18), 1466 (19), 1566 (22), 1591 (57), 2708 (7), 2779 (7), 2864 (15), 2904 (29), 2926 (22), 2956 (28), 3064 (19).

EA: no satisfactory analysis could be obtained.

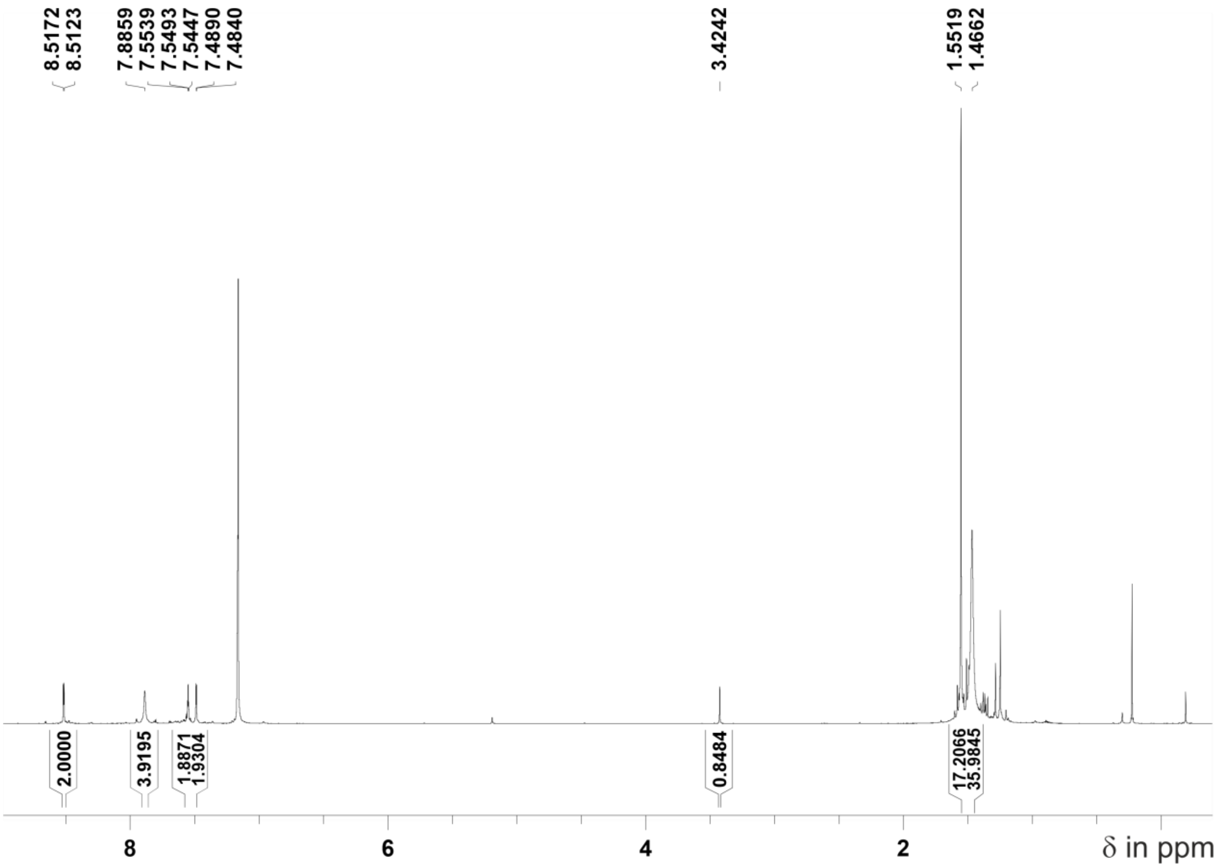

Figure S6: ^{1}H NMR spectrum of **2**.

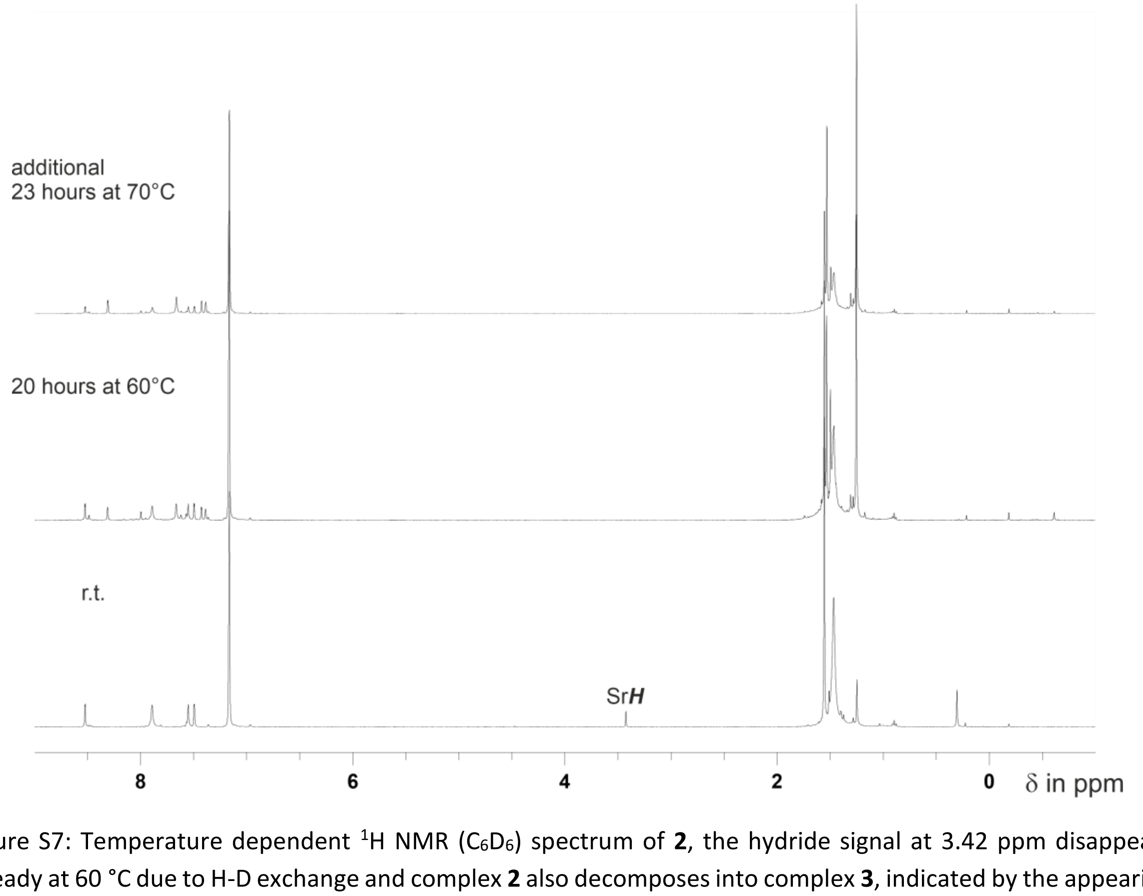

Figure S7: Temperature dependent ^{1}H NMR (C_6D_6) spectrum of **2**, the hydride signal at 3.42 ppm disappears already at 60 °C due to H-D exchange and complex **2** also decomposes into complex **3**, indicated by the appearing chemical shifts.

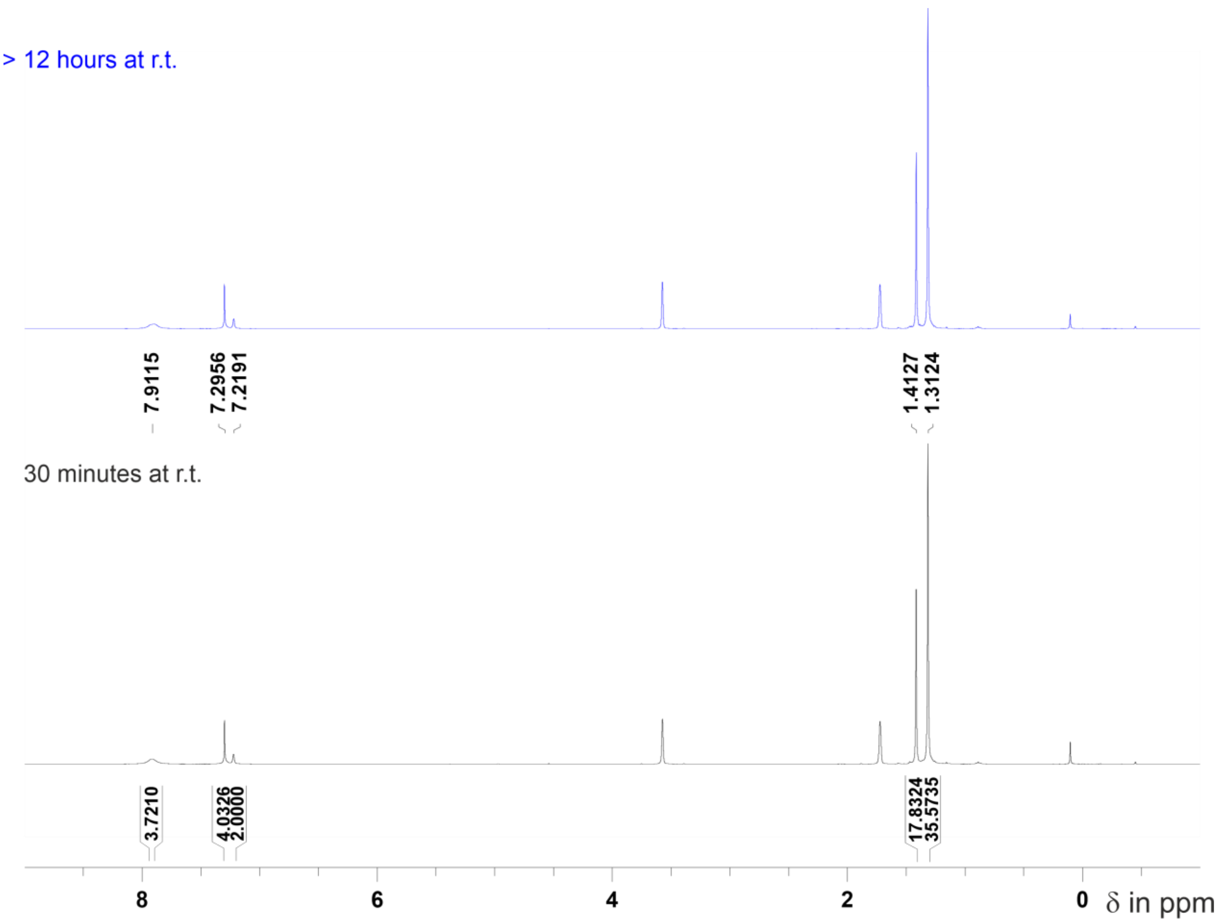

Figure S8: ^{1}H NMR spectrum of **2** in THF-D_8, the hydride signal is not observed.

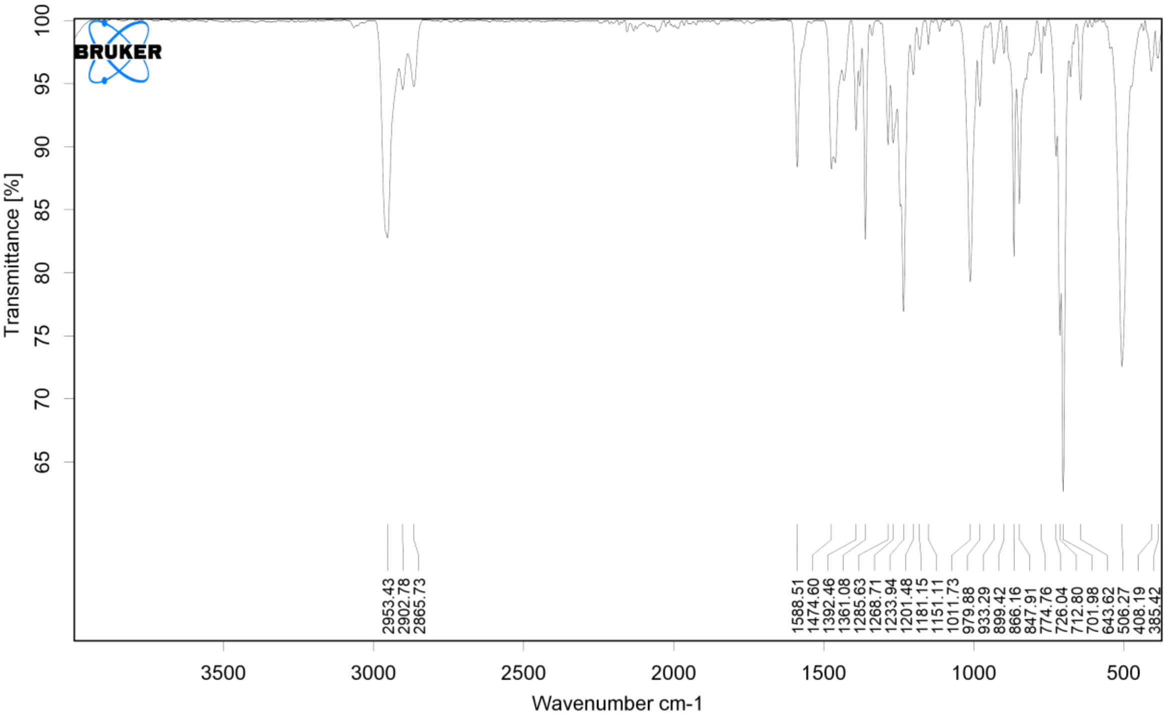

Figure S9: IR spectrum of **2**.

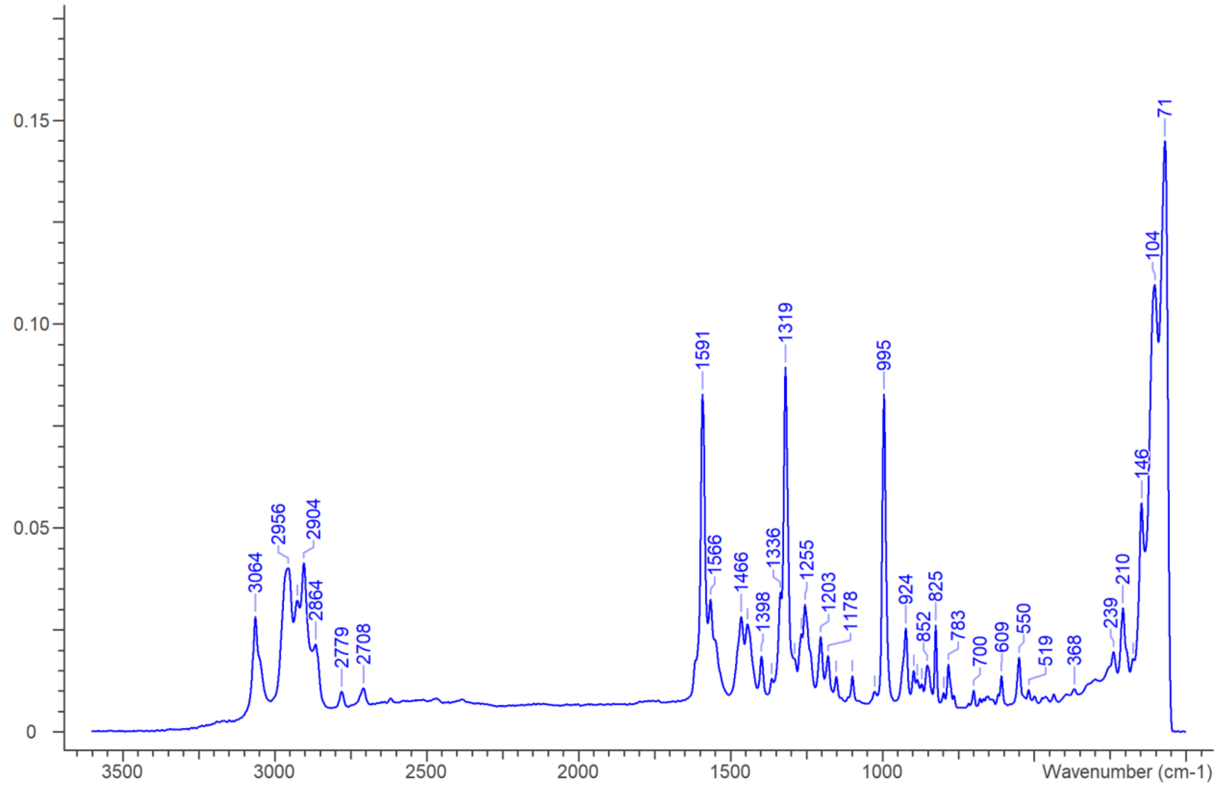

Figure S10: Raman spectrum of **2**.

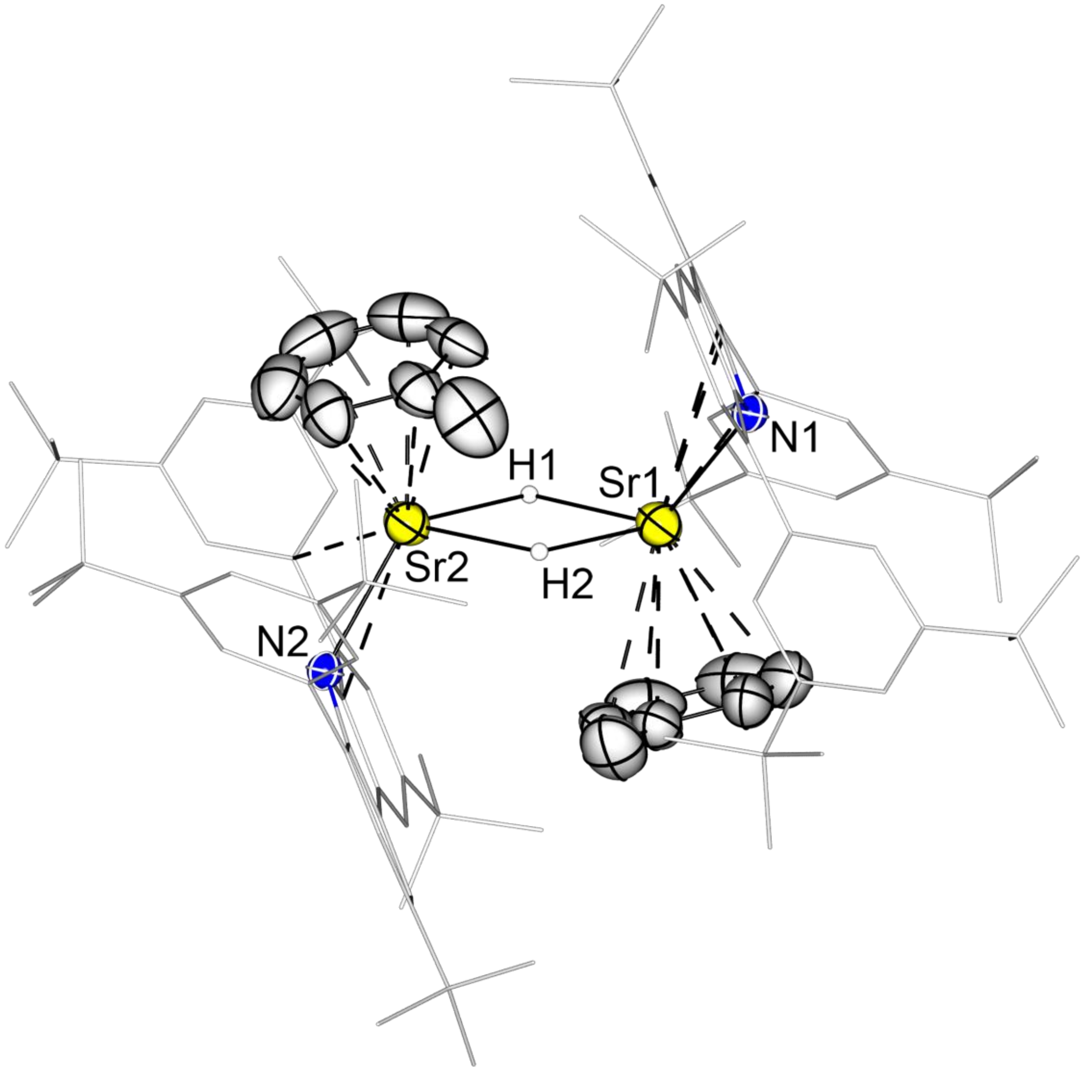

Figure S11: Molecular structure of **2b**. Thermal ellipsoids with 50% probability at 200 K. Selected interatomic distances [Å]: Sr1-H1 2.336(13), Sr2-H2 2.372(13), Sr1-N1 2.513(3), Sr2-N2 2.506(3).

2.3 (^{dtbp}Cbz)$_2$Sr (**3**)

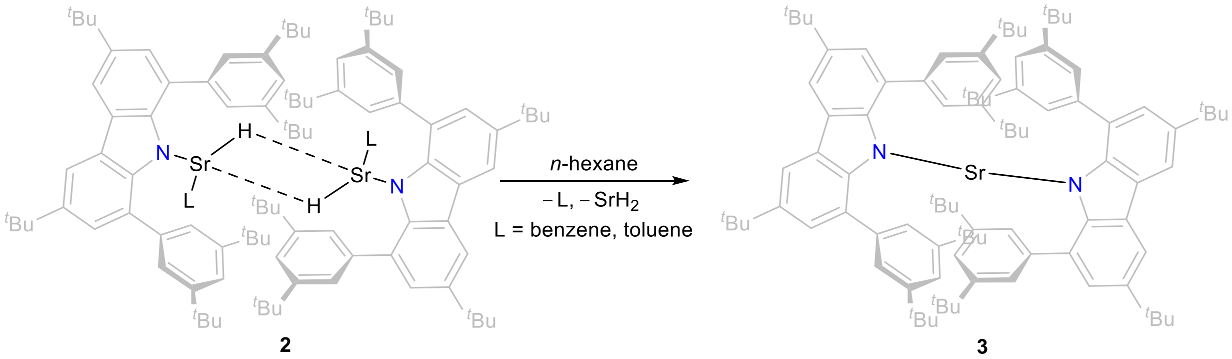

A sample of RSrH·toluene (120 mg, 0.072 mmol) was redissolved in 10 mL *n*-hexane. The yellow solution was left at ambient temperature for a week, and then slowly concentrated to incipient crystallisation (approx. 1 mL). Storage at ambient temperature afforded a yellow crystalline material. The supernatant was decanted and the crystals were dried in vacuo (44 mg, 0.031 mmol, 43%).

^{1}H NMR (400 MHz, C_6D_6): δ (ppm) = 1.25 (br, 36 H, Ar-^{t}Bu), 1.53 (s, 18 H, Carb-^{t}Bu), 7.38 (br, 2 H, *p*-C*H*), 7.41 (br, 2 H, $C^{2,7}H$), 7.65 (br, 4 H, *o*-C*H*), 8.29 (br, 2 H, $C^{4,5}H$).

^{13}C{^{1}H} NMR (100.7 MHz, C_6D_6): δ (ppm) = 31.97 (s, Ar-C(CH_3)$_3$), 32.59 (s, Carb-C(CH_3)$_3$), 34.87 (s, Carb-*C*(CH_3)$_3$), 35.20 (s, Ar-*C*(CH_3)$_3$), 116.91 (s, $C^{4,5}$), 121.81 (s, *p*-*C*H), 123.25 (s, *o*-*C*H), 125.43 (s, $C^{2,7}$), 126.54 (s, $C^{1,8}$), 127.23 (s, $C^{4a,4b}$), 138.99 (s, $C^{3,6}$), 143.99 (s, *i*-*C*), 148.04 (s, $C^{8a,9a}$), 152.19 (s, *m*-*C*).

IR (ATR): ũ (cm^{-1}) = 2954 (s), 2866 (w), 1589 (m), 1462 (w), 1392 (w), 1361 (m), 1287 (m), 1228 (vs), 1202 (w), 1181 (w), 1115 (w), 982 (w), 932 (vw), 899 (vw), 866 (m), 845 (m), 762 (vw), 718 (m), 698 (w), 676 (vw), 645 (w), 518 (vw), 499 (vw), 463 (vw), 418 (vw).

EA: no satisfactory analysis could be obtained.

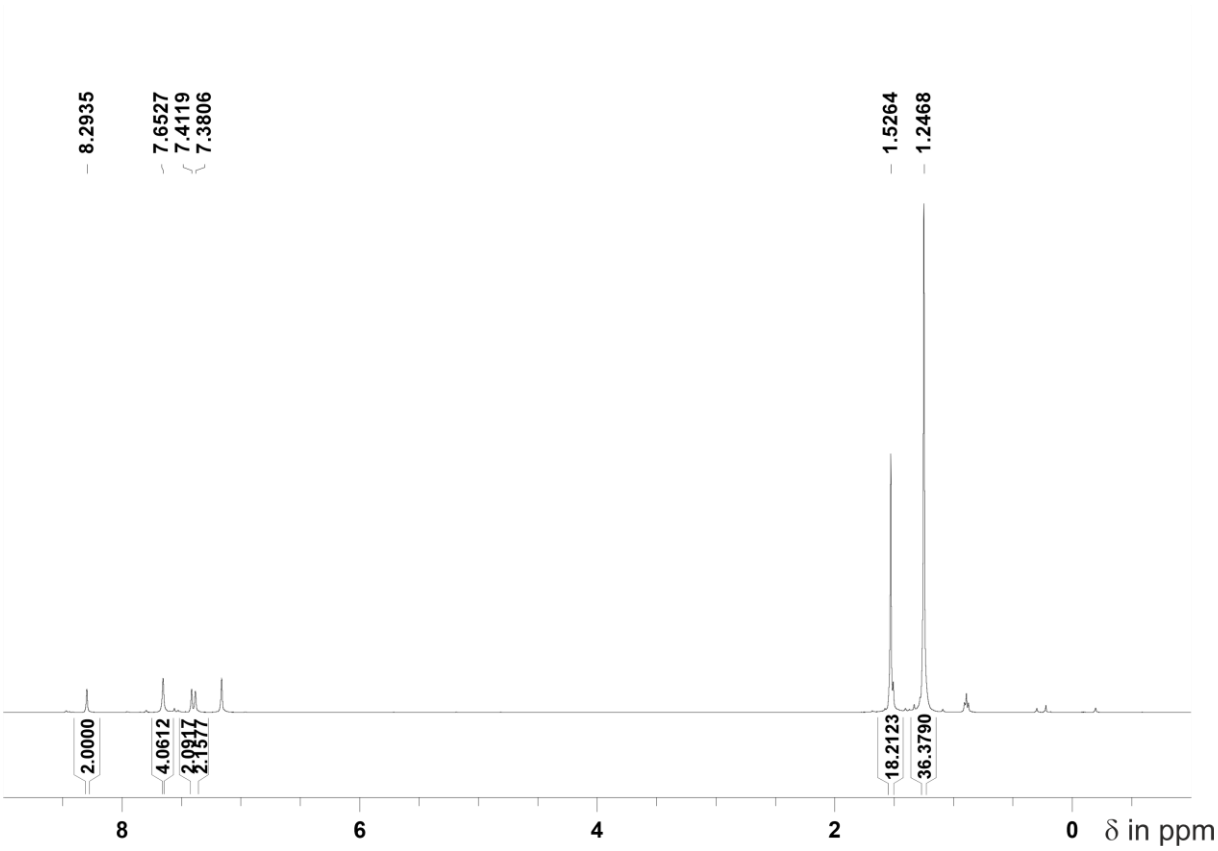

Figure S12: ^{1}H NMR spectrum of **3**.

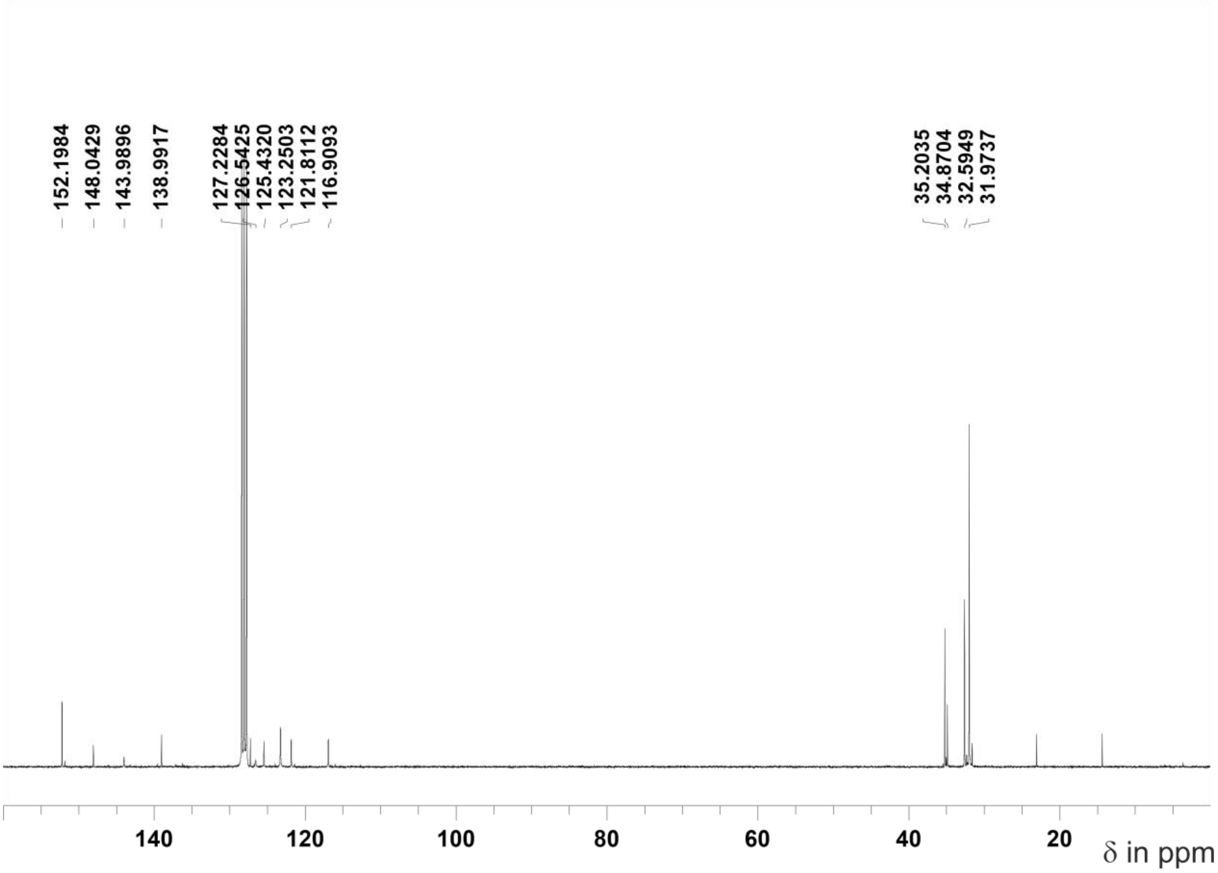

Figure S13: $^{13}C\{^{1}H\}$ NMR spectrum of **3**.

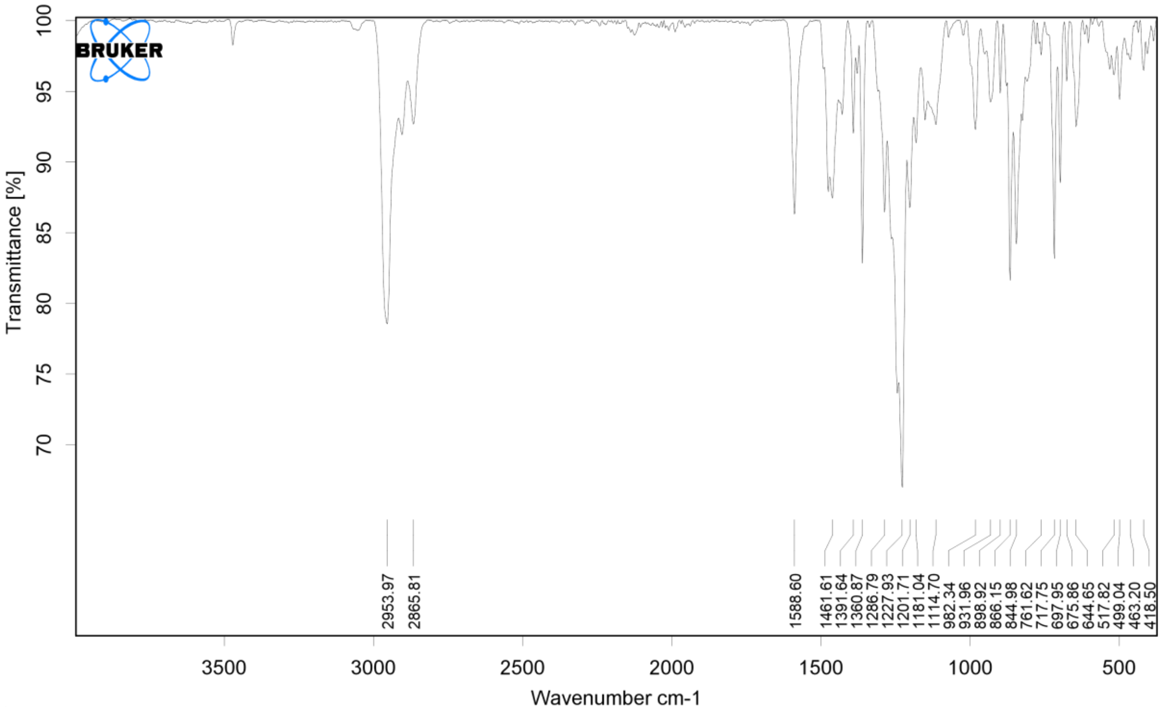

Figure S14: IR spectrum of **3**.

2.4 [^{dtbp}CbzSr(OCH)]$_2$ (**4**)

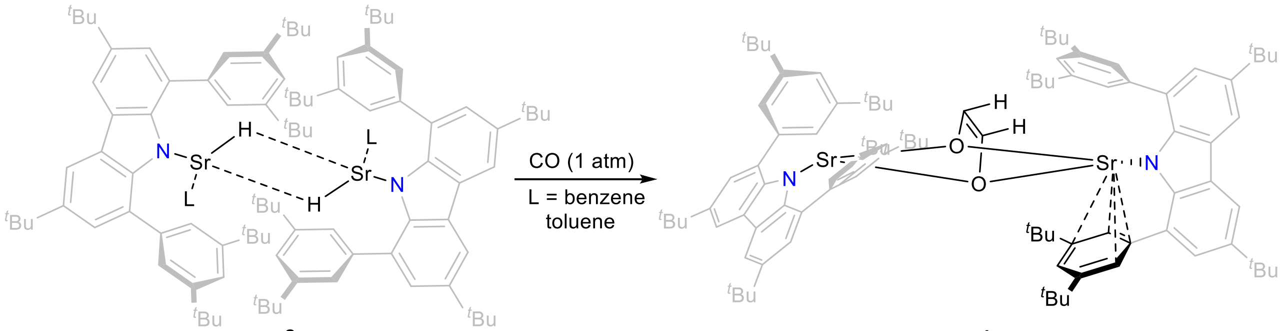

2 **4**

Freshly prepared hydride **2** (80.0 mg, 0.054 mmol) was suspended in 4 mL benzene. The suspension was degassed twice and afterwards exposed to a carbon monoxide atmosphere. Within some minutes, the suspension turns bright orange and the consumption of the hydride **2** is observed by a vanish of the yellow precipitate of the hydride. Concentrating the resulting orange solution yielded suitable crystals for XRD. Crystalline yield: 27 mg, 0.017 mmol, 31%.

The NMR characterisation data were obtained from a NMR reaction with freshly prepared hydride.

IR data are obtained from the briefly dried crystalline material.

^{1}H NMR (400 MHz, C_6D_6): δ (ppm) = 1.14 (s, 36 H, Ar-^{t}Bu), 1.61 (s, 18 H, Carb-^{t}Bu), 3.05 (br, 1 H, OC*H*), 7.36 (br, 2 H, *p*-C*H*), 7.56 (br, 2 H, $C^{2,7}H$), 7.73 (br, 4 H, *o*-C*H*), 8.61 (br, 2 H, $C^{4,5}H$).

^{13}C{^{1}H} NMR (100.7 MHz, C_6D_6): δ (ppm) = 31.64 (s, Ar-C(CH_3)$_3$), 32.74 (s, Carb-C(CH_3)$_3$), 35.07 (s, Carb-*C*(CH_3)$_3$), 35.25 (s, Ar-*C*(CH_3)$_3$), 77.17 (s, O*C*(H)=*C*(H)O), 116.22 (s, $C^{4,5}$), 122.04 (s, *p*-CH), 123.26 (s, $C^{2,7}$), 123.85 (s, *o*-CH), 126.64 (s, $C^{4a,4b}$), 127.09 (s, $C^{1,8}$), 138.18 (s, $C^{3,6}$), 145.19 (s, *i*-*C*), 149.02 (s, $C^{8a,9a}$), 152.99 (s, *m*-*C*).

IR (ATR): ũ (cm^{-1}) = 2954 (vs), 2904 (w), 2865 (w), 1590 (s), 14914558 (m), 1477 (m), 1462 (m), 1393 (m), 1362 (vs), 1287 (s), 1244 (vs), 1202 (w), 868 (vs), 846 (w), 824 (vw), 769 (vw), 719 (s), 698 (w), 675 (m), 646 (w), 603 (vw), 497 (w), 464 (vw), 412 (vw), 395 (vw).

EA: no satisfactory analysis could be obtained.

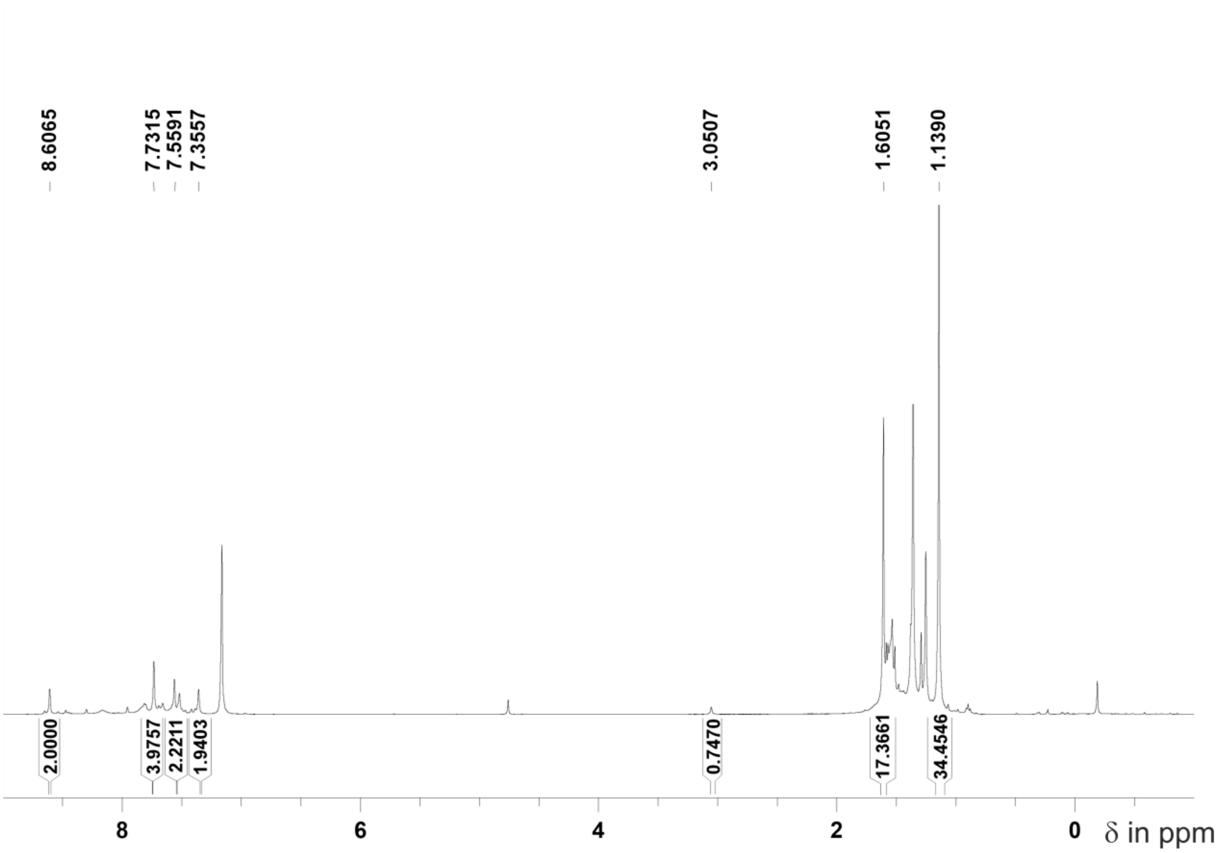

Figure S15: ^{1}H NMR spectrum of **4**.

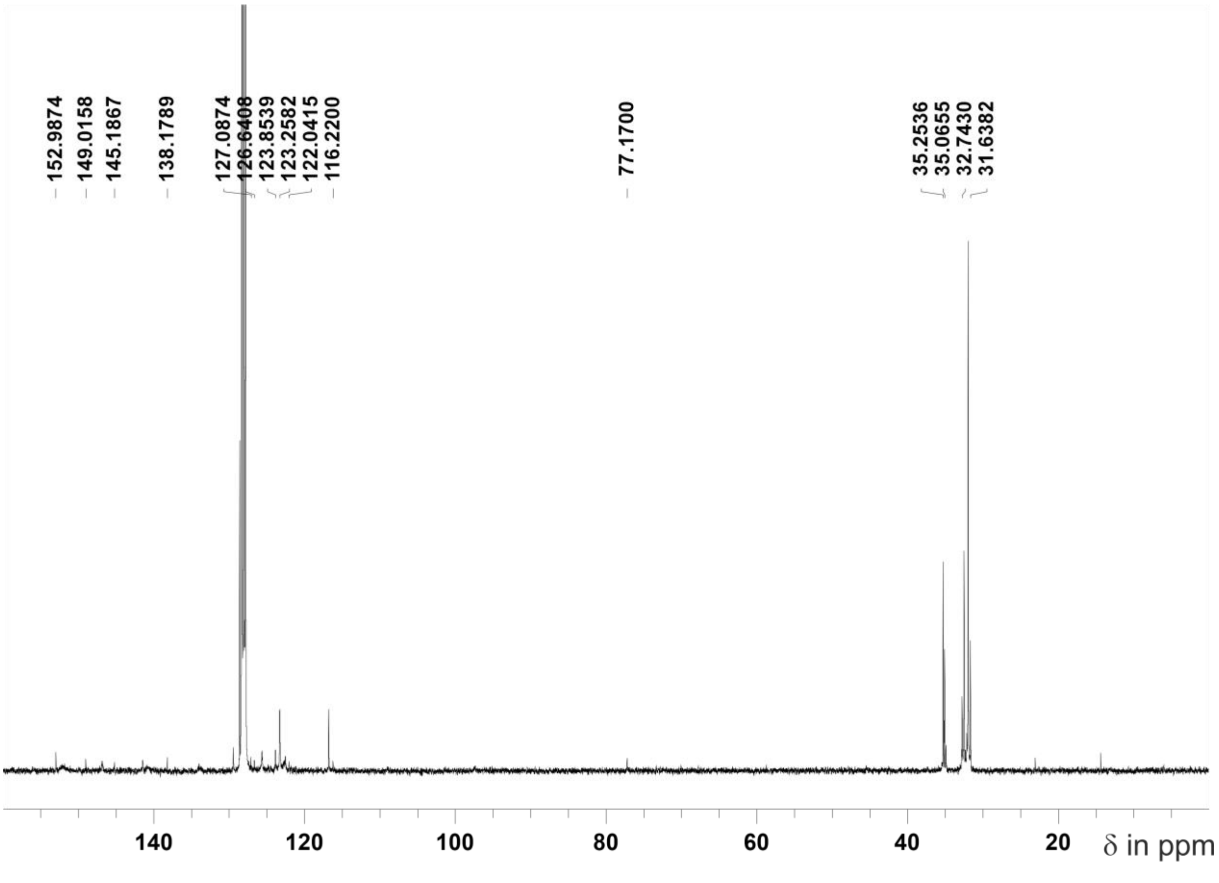

Figure S16: $^{13}C\{^{1}H\}$ NMR spectrum of **4**.

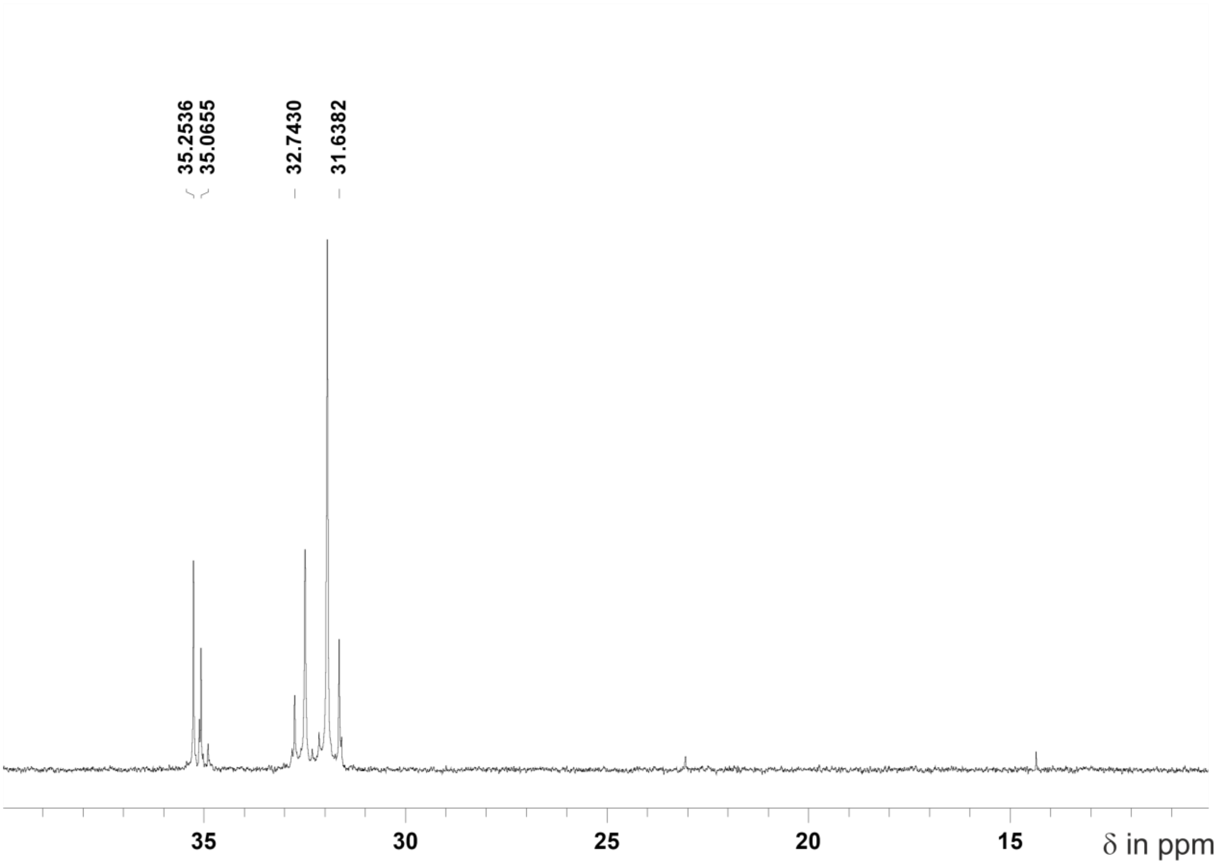

Figure S17: $^{13}C\{^1H\}$ NMR spectrum of **4** (10-40ppm).

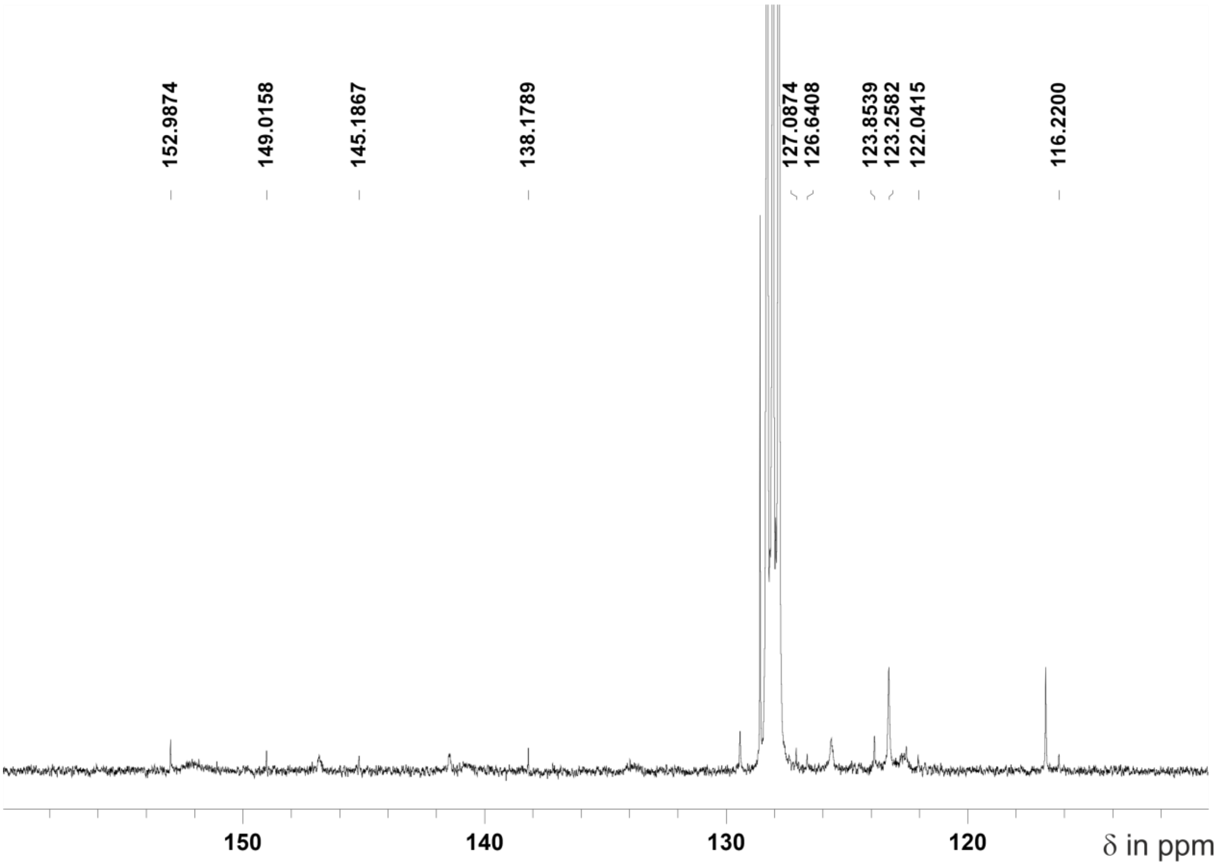

Figure S18: $^{13}C\{^1H\}$ NMR spectrum of **4** (110-160ppm).

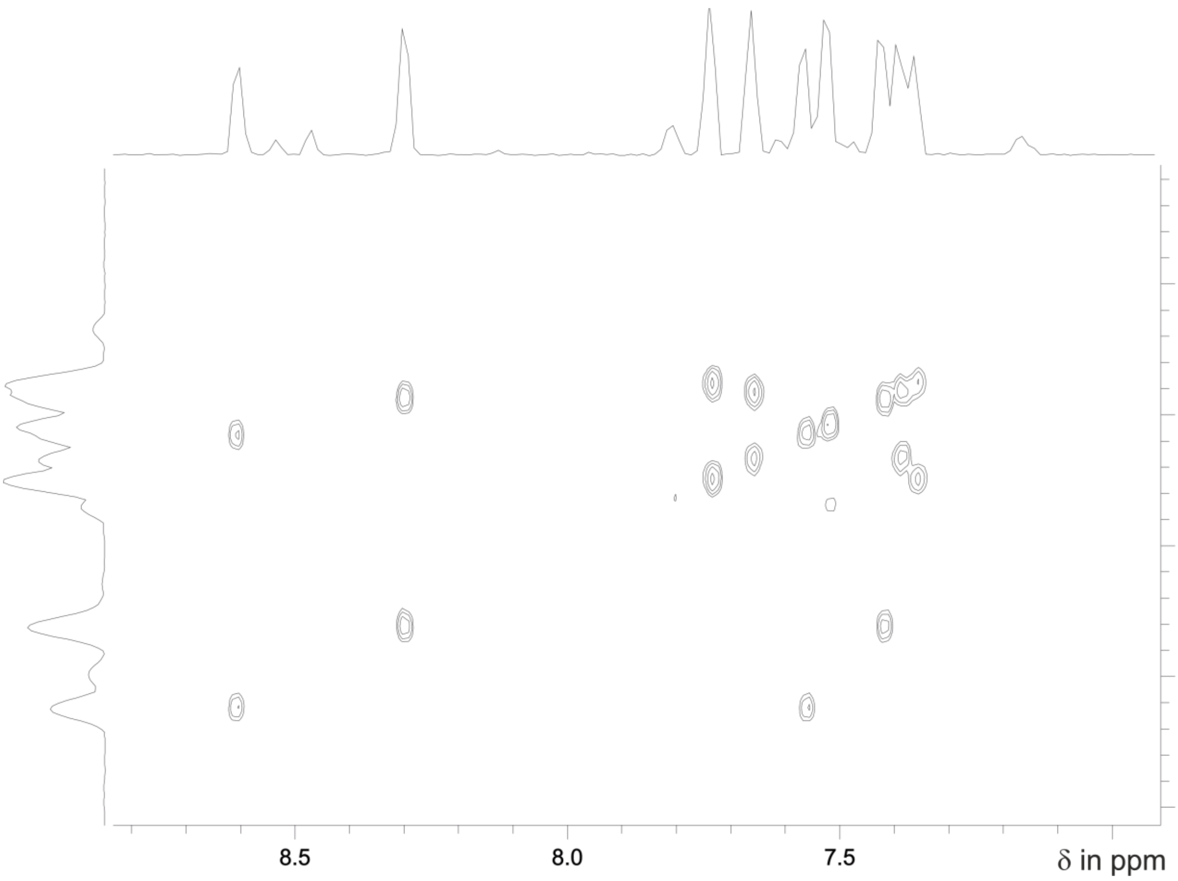

Figure S19: ^{1}H-^{1}H COSY NMR spectrum of **4** (relevant aromatic region).

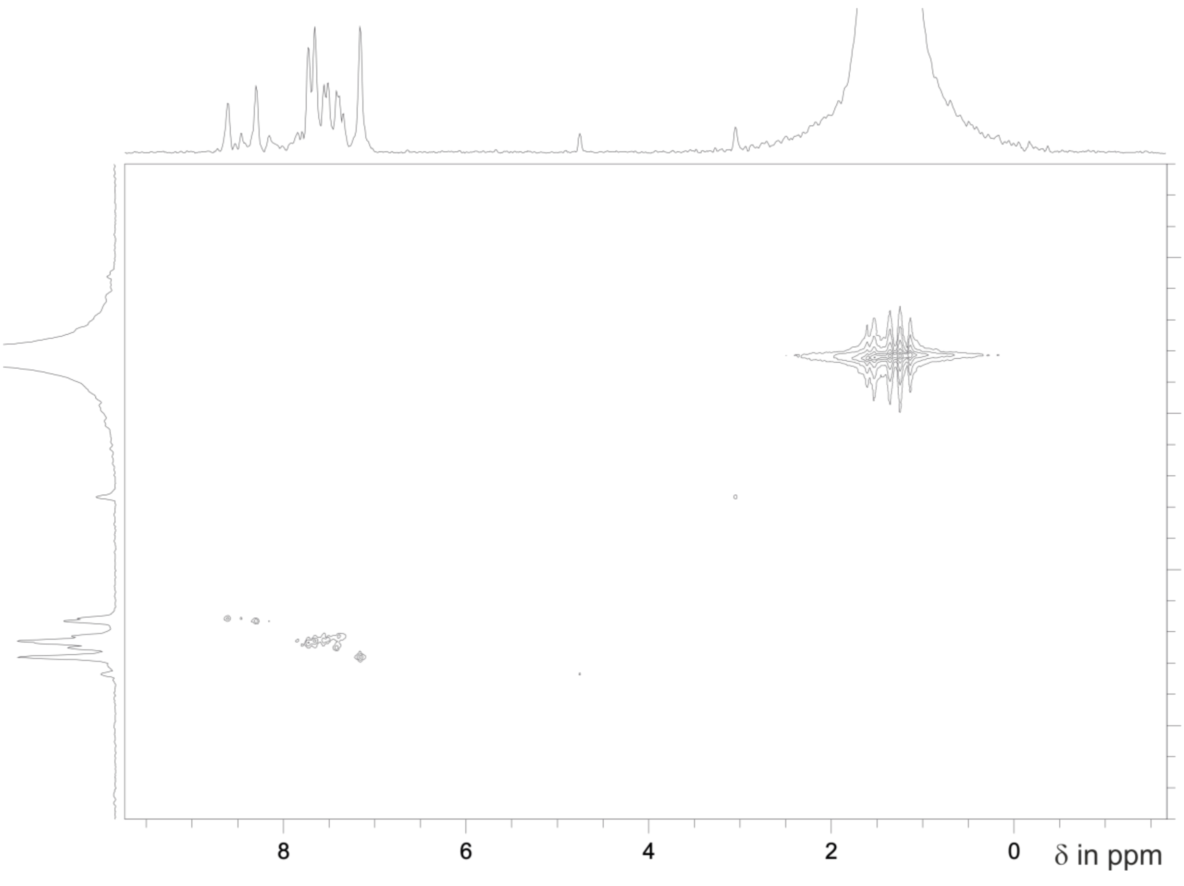

Figure S20: ^{1}H-^{13}C HMQC NMR spectrum of **4**.

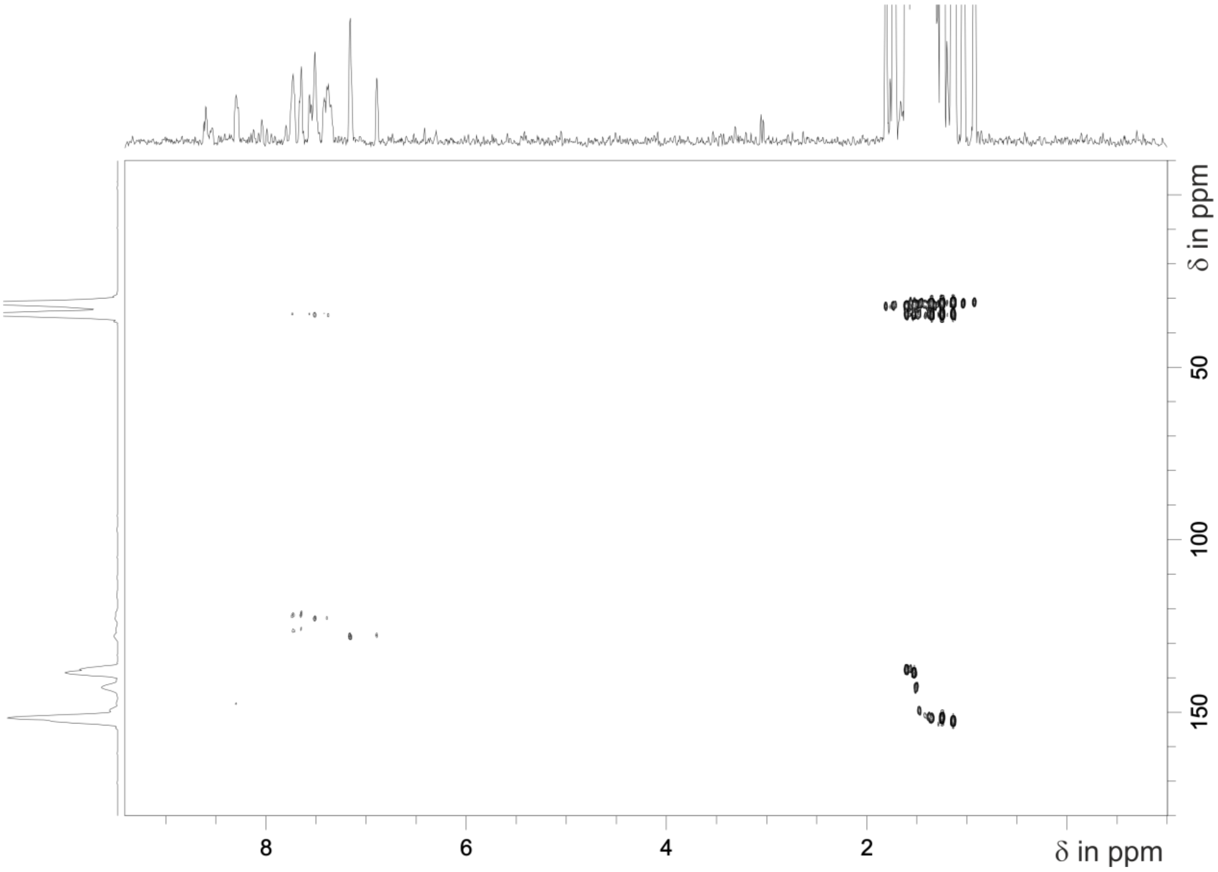

Figure S21: ^{1}H-^{13}C HMBC NMR spectrum of **4**.

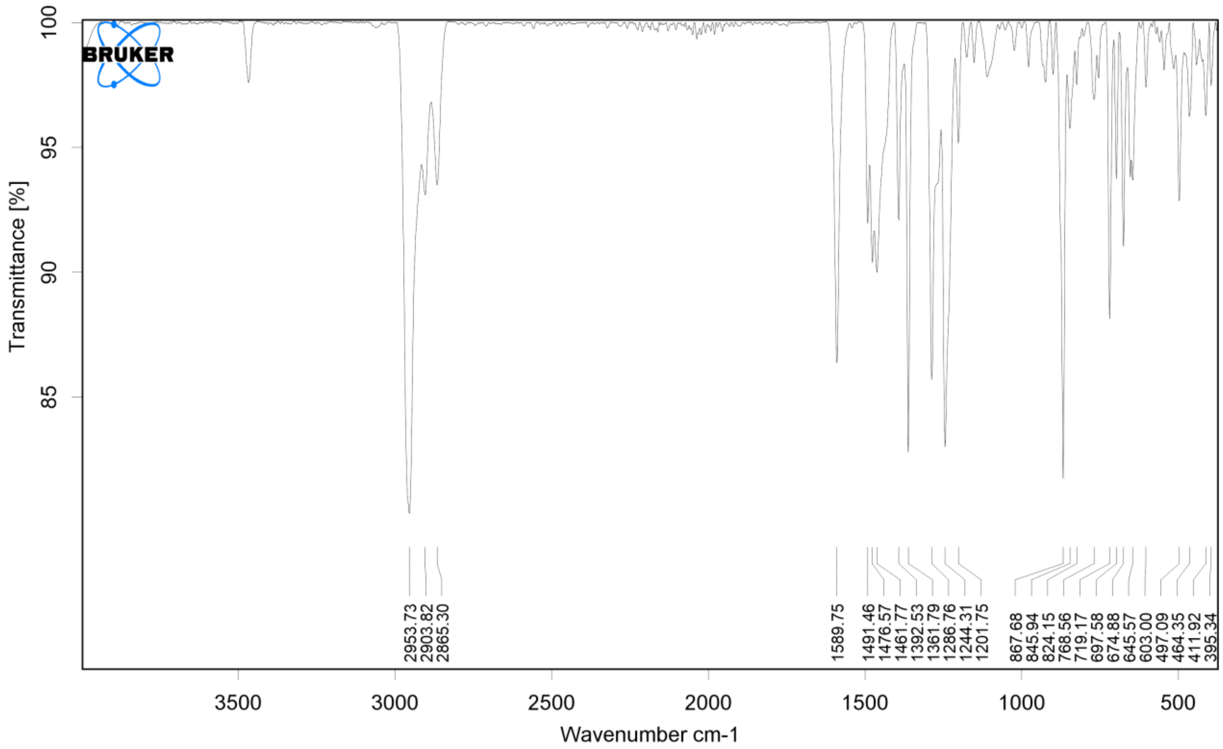

Figure S22: IR spectrum of **4**, *v*(N-H) not assigned.

2.5 [^{dtbp}CbzSr(PhNNPh)]$_2$ (**5**)

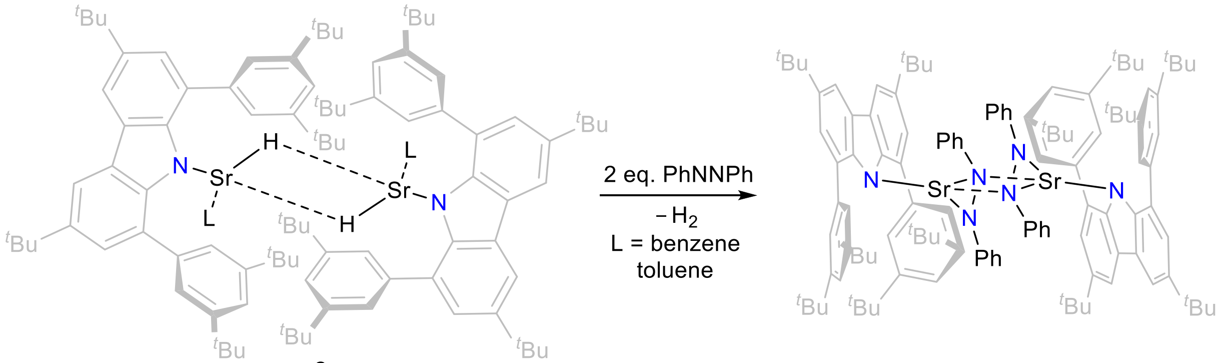

2 **5**

Freshly prepared hydride **2** (139 mg, 0.094 mmol) was suspended in 8.0 mL toluene. To that suspension, azobenzene (36.1 mg, 0.198 mmol, 2.11 eq.) was added. The reaction mixture immediately turned dark green and was stirred for one hour at room temperature. Afterwards, the mixture was filtered via syringe filter and concentrated until incipient crystallization. Within three days, dark green crystals formed suitable for XRD analysis. The supernatant was removed, the crystals washed with *n*-hexane and dried briefly. Crystalline yield: 47 mg, 0.025 mmol, 27%. The characterisation data were obtained from the crystalline material.

^{1}H NMR (400 MHz, C_6D_6): δ (ppm) = 1.26 (s, 36 H, Ar-^{t}Bu), 1.54 (s, 18 H, Carb-^{t}Bu), 7.07-7.20 (m, 6 H, Ar*H*), 7.47 (br, 2 H, *p*-C*H*), 7.65 (br, 2 H, $C^{2,7}H$), 8.00-8.06 (m, 4 H, Ar*H*), 8.28 (br, 4 H, *o*-C*H*), 8.65 (br, 2 H, $C^{4,5}H$).

^{13}C{^{1}H} NMR (100.7 MHz, C_6D_6): δ (ppm) = 31.90 (s, Ar-C(CH_3)$_3$), 32.56 (s, Carb-C(CH_3)$_3$), 34.74 (s, Carb-*C*(CH_3)$_3$), 35.30 (s, Ar-*C*(CH_3)$_3$), 116.04 (s, $C^{4,5}$), 121.26 (s, *p*-CH), 123.35 (s, *C*H), 124.58 (s, *o*-CH), 125.47 (s, $C^{2,7}$), 126.63 (s, $C^{4a,4b}$), 129.30 (s, *C*H), 131.14 (s, *C*H), 139.51 (s, $C^{3,6}$), 143.58 (s, *i-C*), 147.04 (s, $C^{8a,9a}$), 152.28 (s, *m-C*), 153.24 (s, *i-C*).

^{15}N NMR (40.6 MHz, C_6D_6): δ (ppm) = 110.1 (s, *N*Ph).

IR (ATR): ṽ (cm^{-1}) = 3056 (vw), 2950 (s), 2902 (vw), 2863 (vw), 1587 (m), 1553 (vw), 1466 (vs), 1388 (w), 1360 (s), 1287 (m), 1242 (vs), 1225 (vs), 1177 (vw), 1158 (m), 1123 (vw), 1072 (vw), 1018 (vw), 980 (vw), 932 (vw), 898 (w), 882 (w), 864 (s), 852 (w), 840 (w), 822 (w), 795 (vw), 771 (vw), 750 (s), 726 (vs), 717 (m), 696 (vs), 676 (vw), 644 (w), 619 (vw), 565 (vw), 546 (vw), 535 (vw), 523 (m), 503 (vw), 464 (w), 431 (vw), 397 (vw).

EA found (calc. for $C_{60}H_{74}N_3Sr$): C 77.79 (77.92), H 7.49 (8.06), N 4.18 (4.54).

UV/VIS: Toluene, λ_{max} (nm) = 569 ($\varepsilon = 5.54 \cdot 10^2$ L mol^{-1} cm^{-1}), 732 ($\varepsilon = 4.00 \cdot 10^2$ L mol^{-1} cm^{-1}).

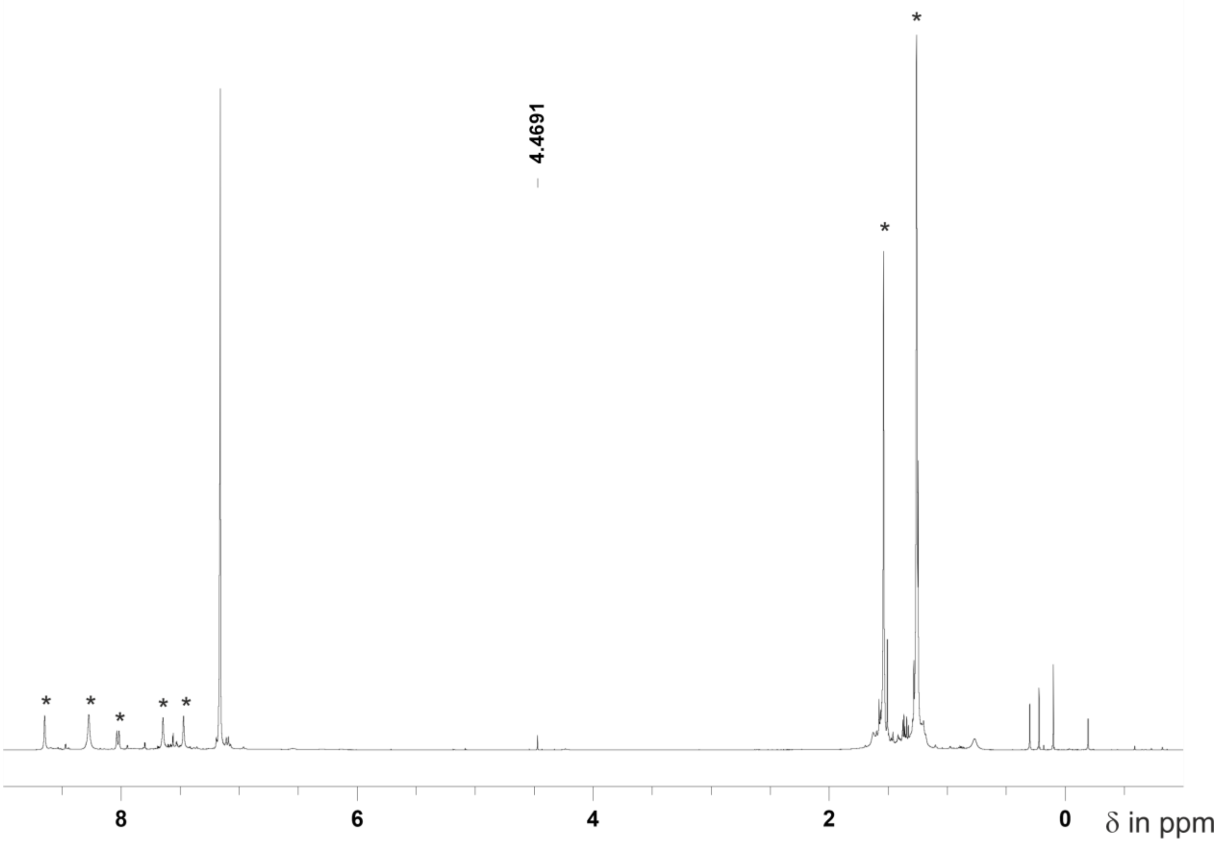

Figure S23: ^{1}H NMR spectrum of the reaction mixture after 5 minutes, H_2 assigned (4.47ppm) and **5** marked.

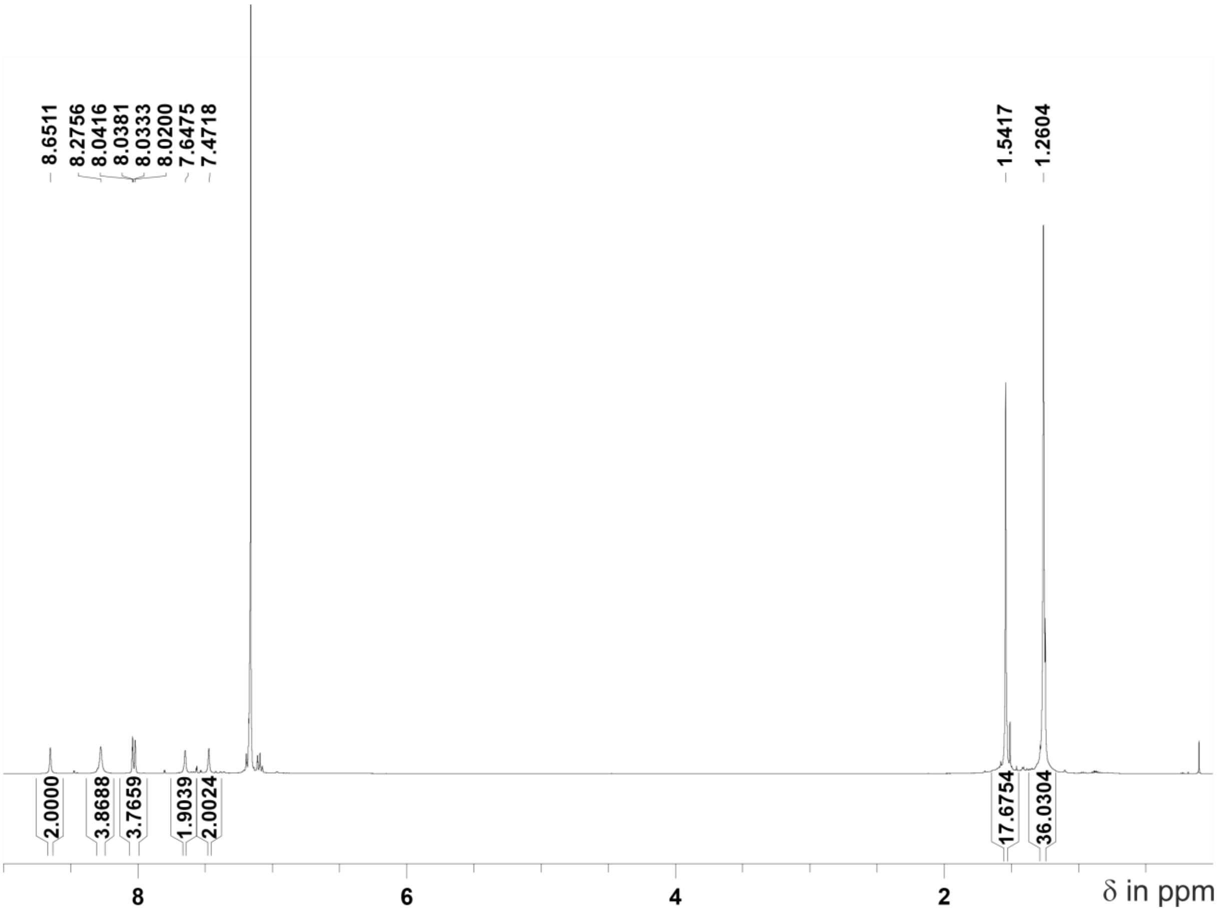

Figure S24: ^{1}H NMR spectrum of **5**.

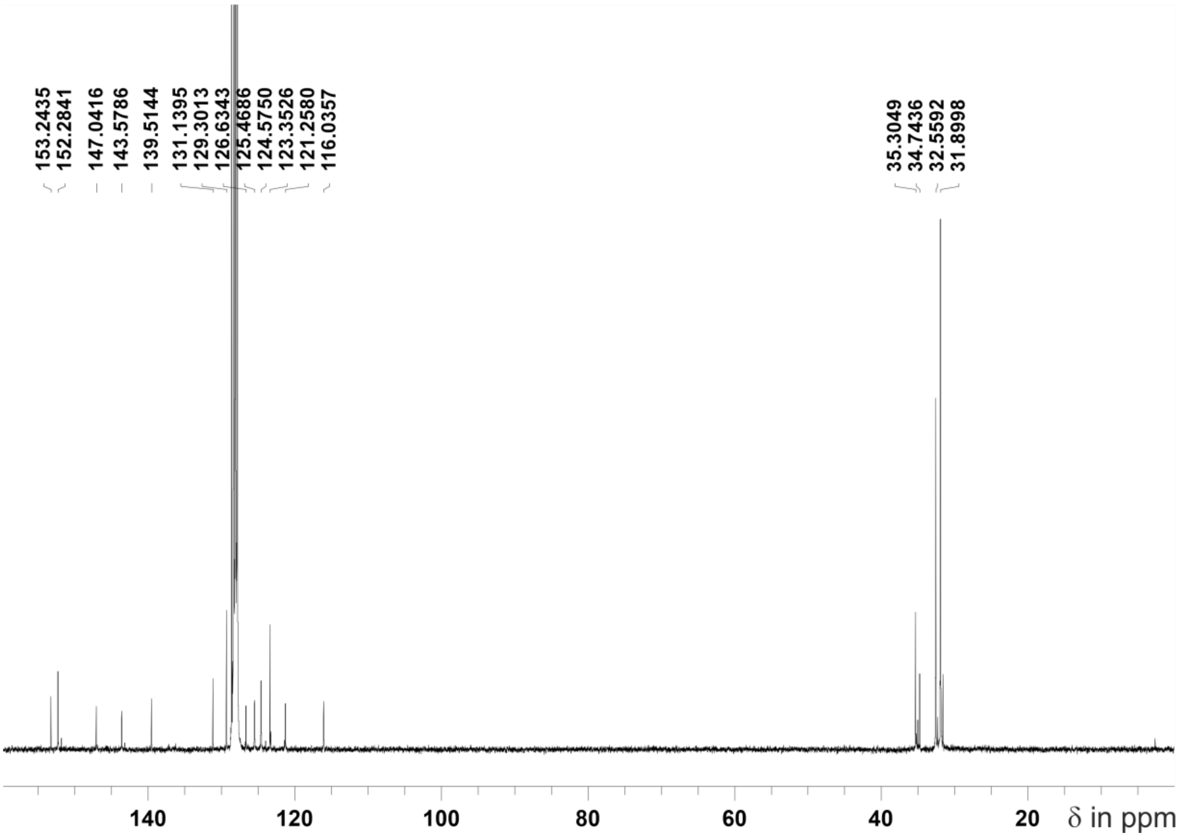

Figure S25: $^{13}C\{^{1}H\}$ NMR spectrum of **5**.

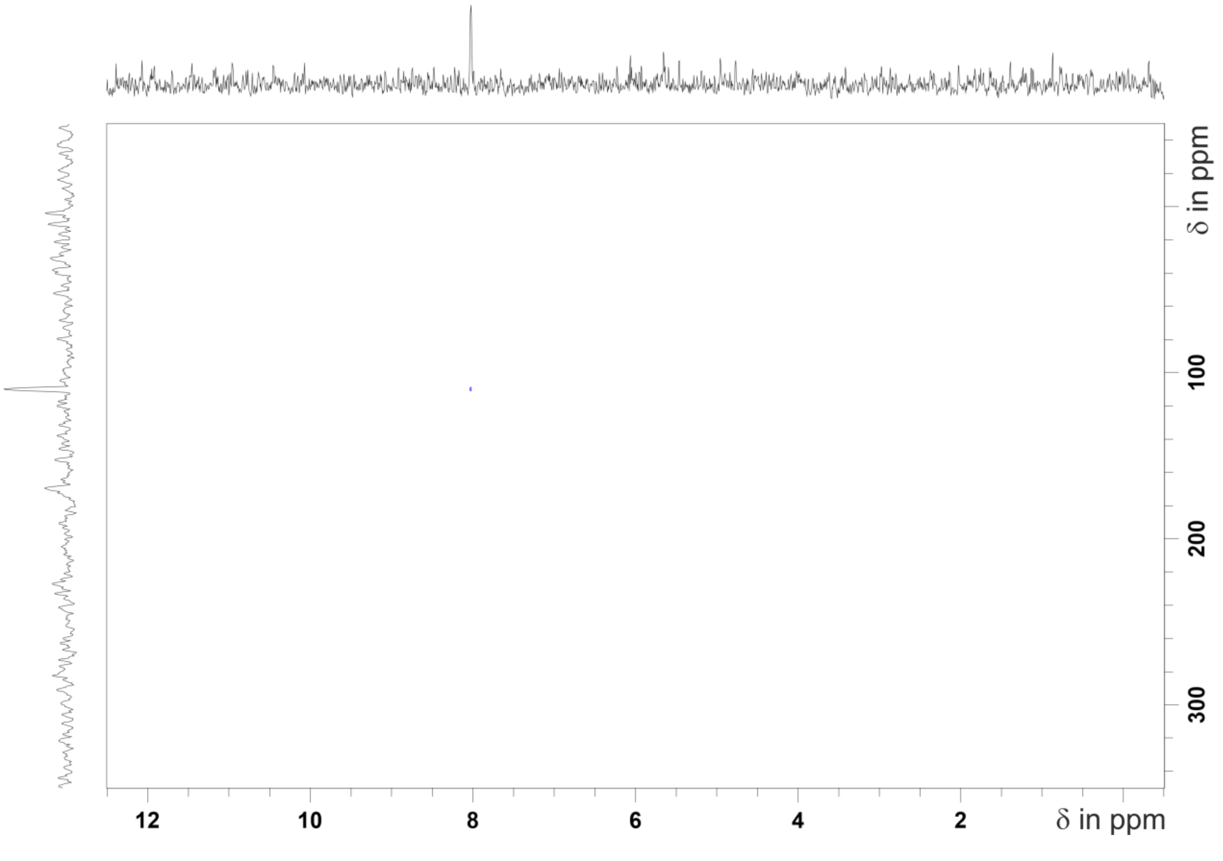

Figure S26: ^{1}H-^{15}N HMBC NMR spectrum of **5**.

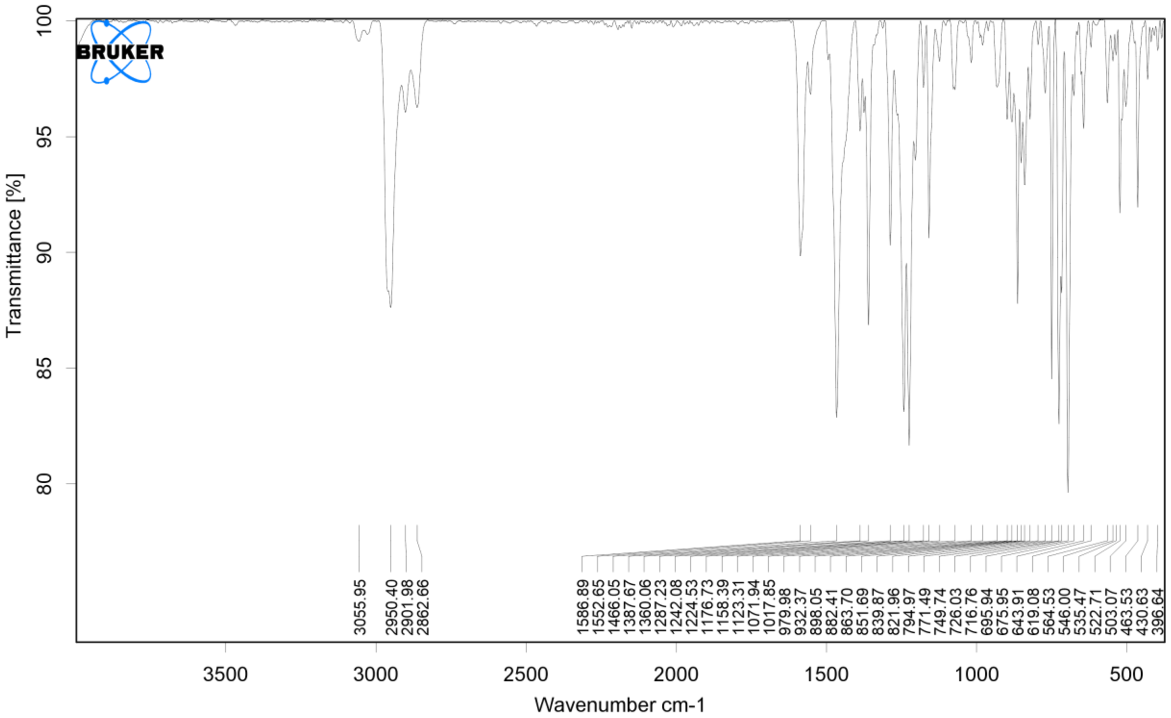

Figure S27: IR spectrum of **5**.

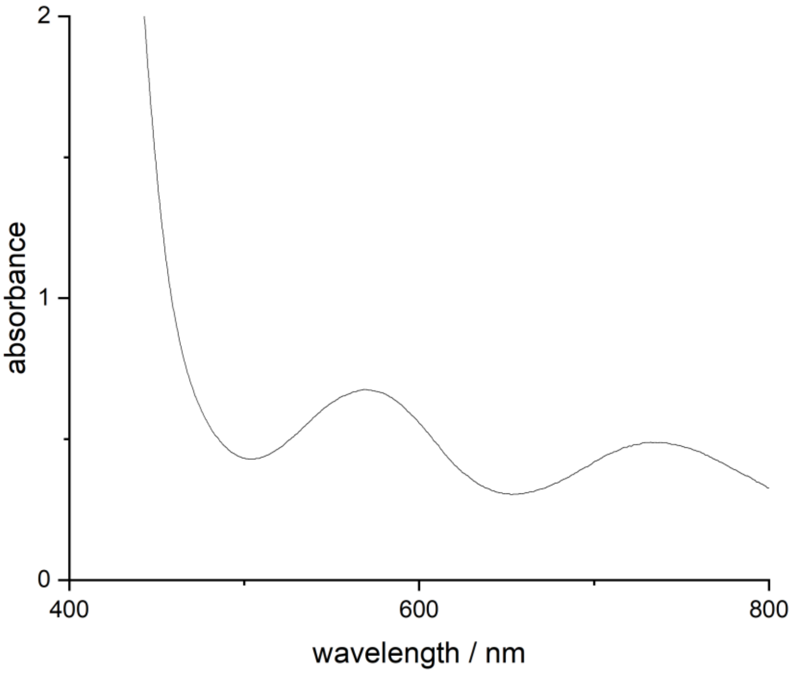

Figure S28: UV-VIS spectrum of **5**.

NMR study regarding the formation of complex **5**

To investigate the formation of complex **5**, the reaction was performed on a NMR scale with an approximate reagent ratio of 1:1: For full conversion to complex **5** 2.00 equivalents are necessary (see synthesis).

The hydride **2** (24 mg, 16.1 µmol, 1.00 eq.) was suspended in 0.6 mL of C_6D_6. To that suspension, azobenzene (2.6 mg, 14.3 µmol, 0.89 eq.) was added. The reaction mixture immediately turned intense yellow, but not dark green. Within less than one hour, ^{1}H and ^{1}H-^{1}H-COSY experiments of the reaction mixture were recorded.

The ^{1}H NMR measurement shows three different species (see Figure S29, S30, S32), indicating fast reactions to the intermediates as well as to final complex **5**. Complex **5** shows the lowest intensities compared to both other species (see integrals of selected usually lowest-field $C^{4,5}H$ protons, Figure S32).

Full assignment of all peaks as well as integration was not performed due to many signals overlapping, especially in the aromatic region. But the aliphatic region shows clearly three signal sets (see Figure S30).

A plausible reaction pathway in the formation of **5** proceeds via hydrometallation of azobenzene with one carbazolyl strontium hydride moiety (see Scheme S1, **I**) and subsequent hydrogen elimination, yielding a dinuclear carbazolyl strontium complex with a bridging and dianionic diphenylhydrazido ligand ($PhNNPh^{2-}$, see Scheme S1, **II**). Intermediate **II** may be able to reduce azobenzene to complex **5**.

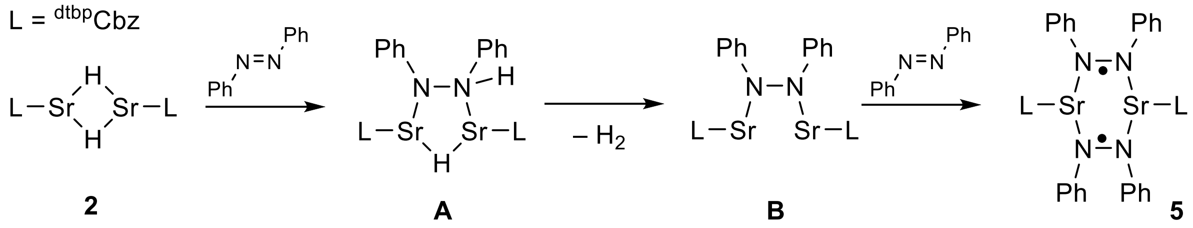

Scheme S1: Plausible pathway for the formation of complex **5**.

One of the observed intermediates features hydrazido moiety (-PhNN(H)Ph) with coupling ($^4J_{HH}$) of the N*H*-proton to the *ortho* protons of the adjacent phenyl substituent (6.55 ppm, see Figure S32-S34), thus assignable as **I**. The ^{1}H NMR measurement shows a broad proton signal at 4.23 ppm. Additionally, the formation of dihydrogen at 4.48 ppm was observed (see Figure S31). Another carbazole-containing intermediate was observed, but no functional ^{1}H signals of hydrides or NH groups could be identified. Therefore, the set of signals can be tentatively assigned to intermediate **II**. After several days, crystals of **II** formed from the reaction solution, allowing unambiguous identification of **II** via scXRD. Lastly, already compound **5** is present in the reaction mixture.

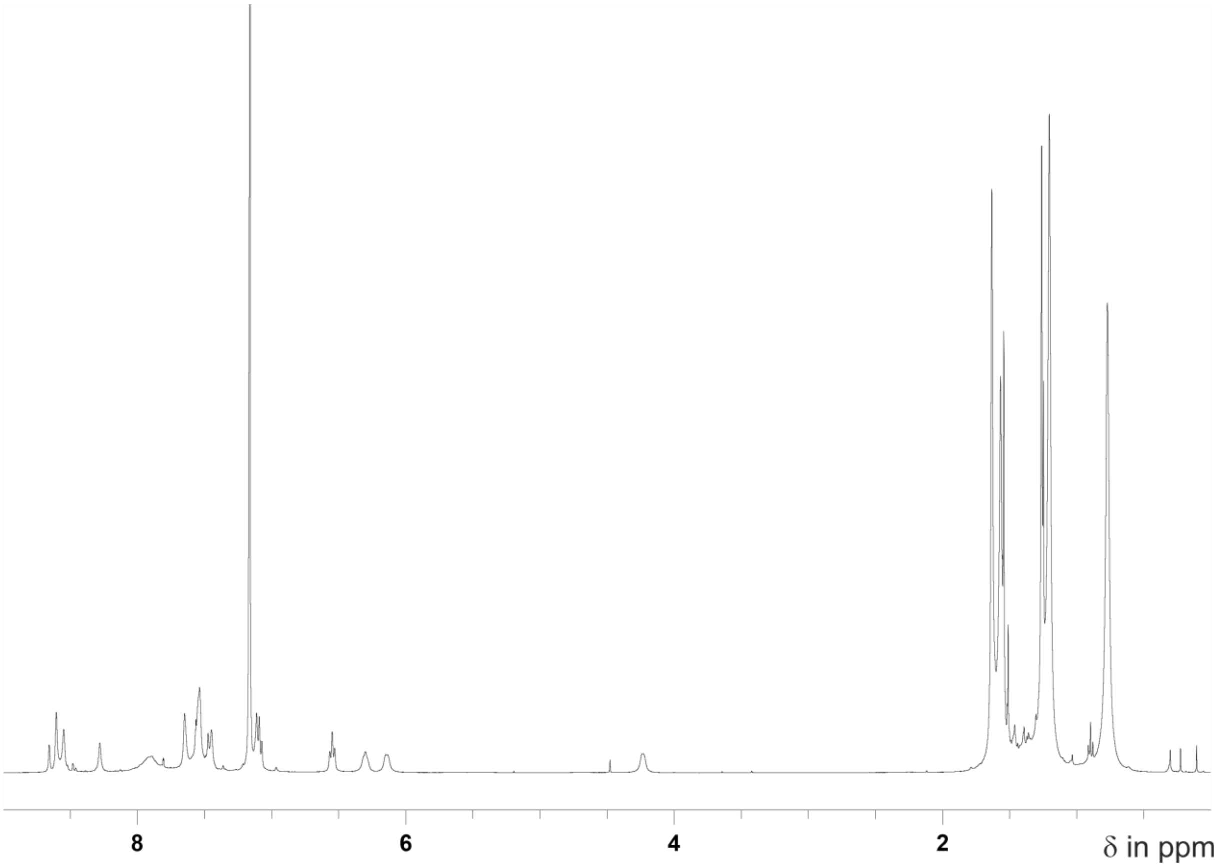

Figure S29: Full ^{1}H NMR spectrum of the reaction mixture with 0.89 eq. of azobenzene.

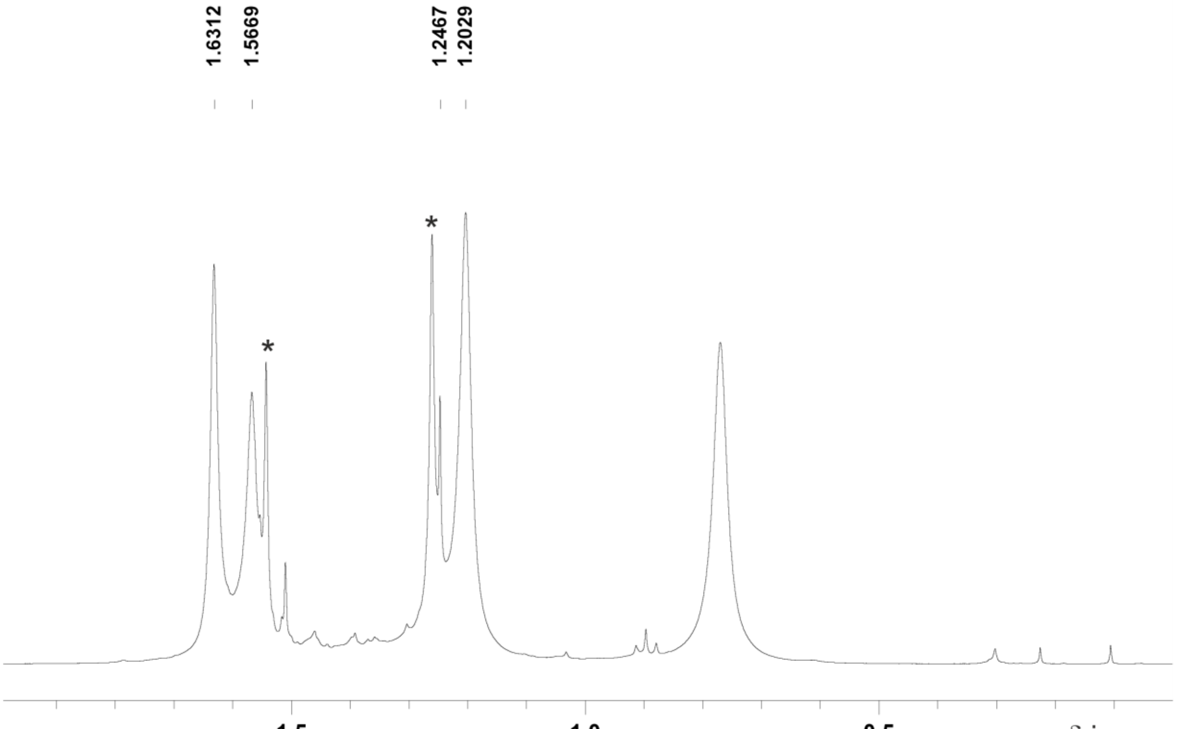

Figure S30: Section (0-2 ppm) of the ^{1}H NMR spectrum of the reaction mixture of **5** with 0.89 eq. of azobenzene, complex **5** marked.

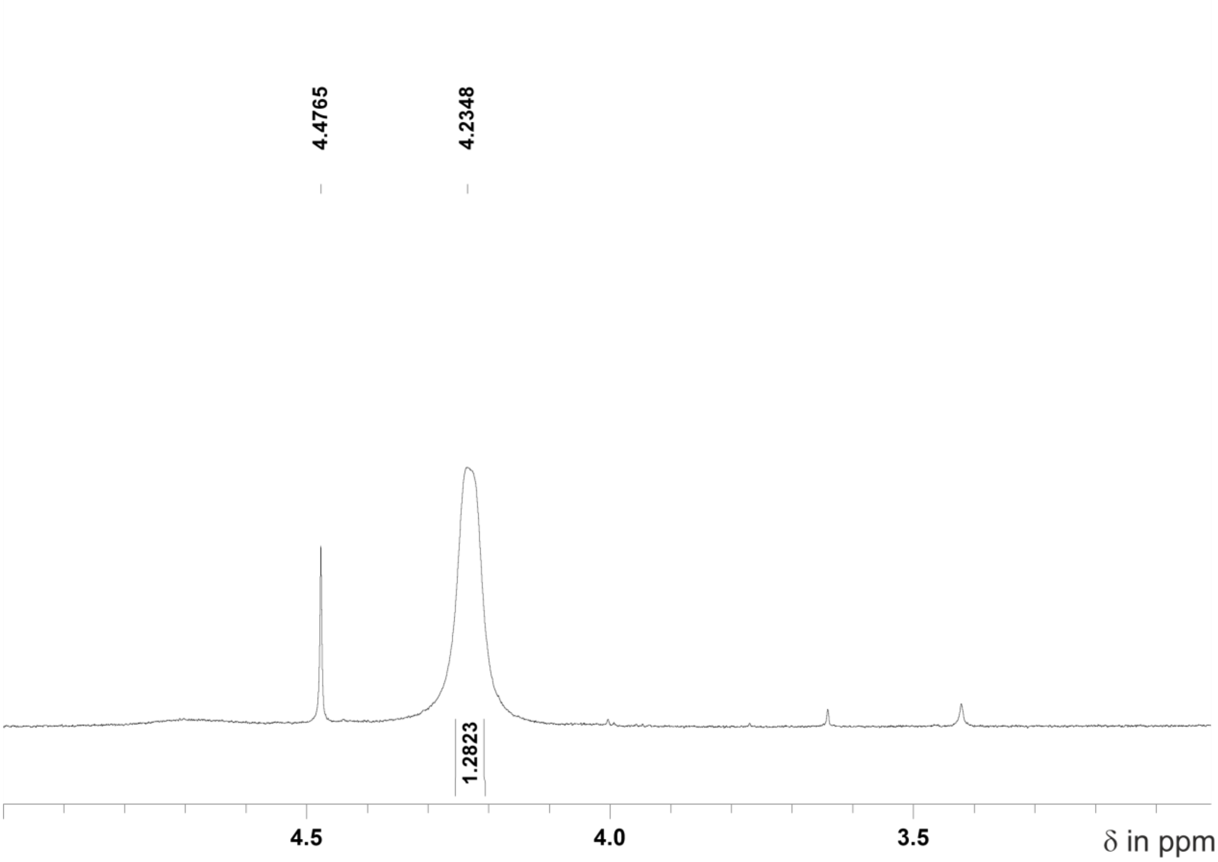

Figure S31: Section (3-5 ppm) of the ^{1}H NMR spectrum of the reaction mixture of **5** with 0.89 eq. of azobenzene.

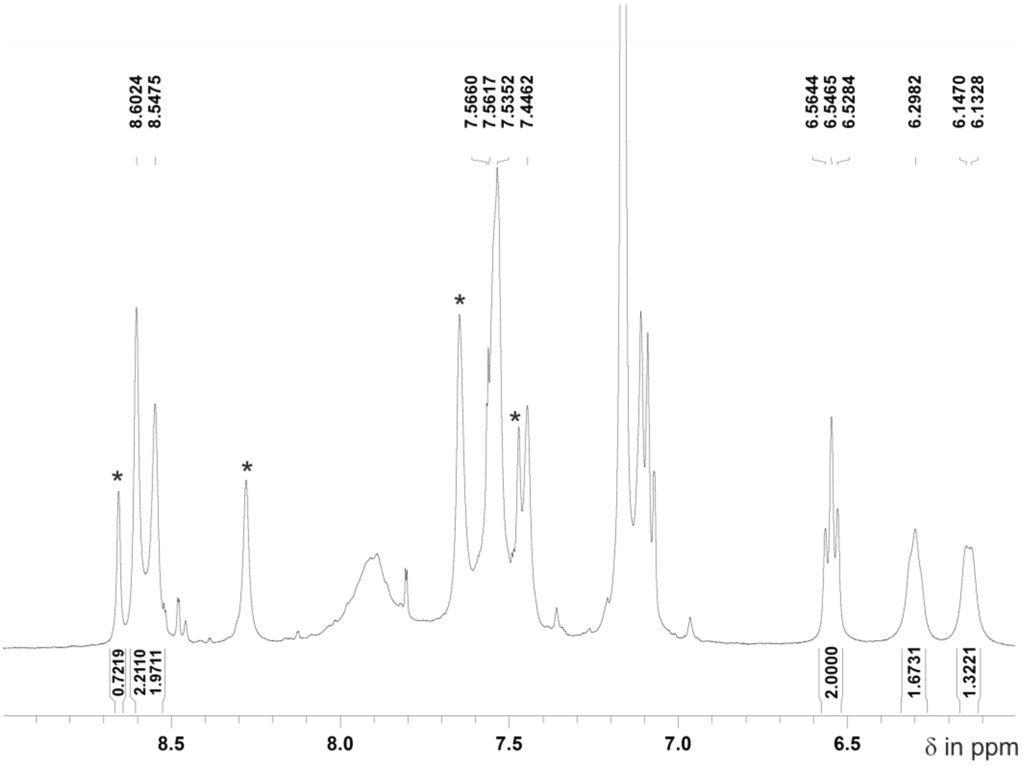

Figure S32: Section (6-9 ppm) of the ^{1}H NMR spectrum of the reaction mixture of **5** with 0.89 eq. of azobenzene, complex **5** marked. Peaks of intermediate species picked. The integrals of the phenyl protons (6.0-6.6 ppm) are in accordance with the N*H* proton within the hydrazido moiety. Lowest-field integrals (8.5-8.7 ppm) show the ratio between the three species.

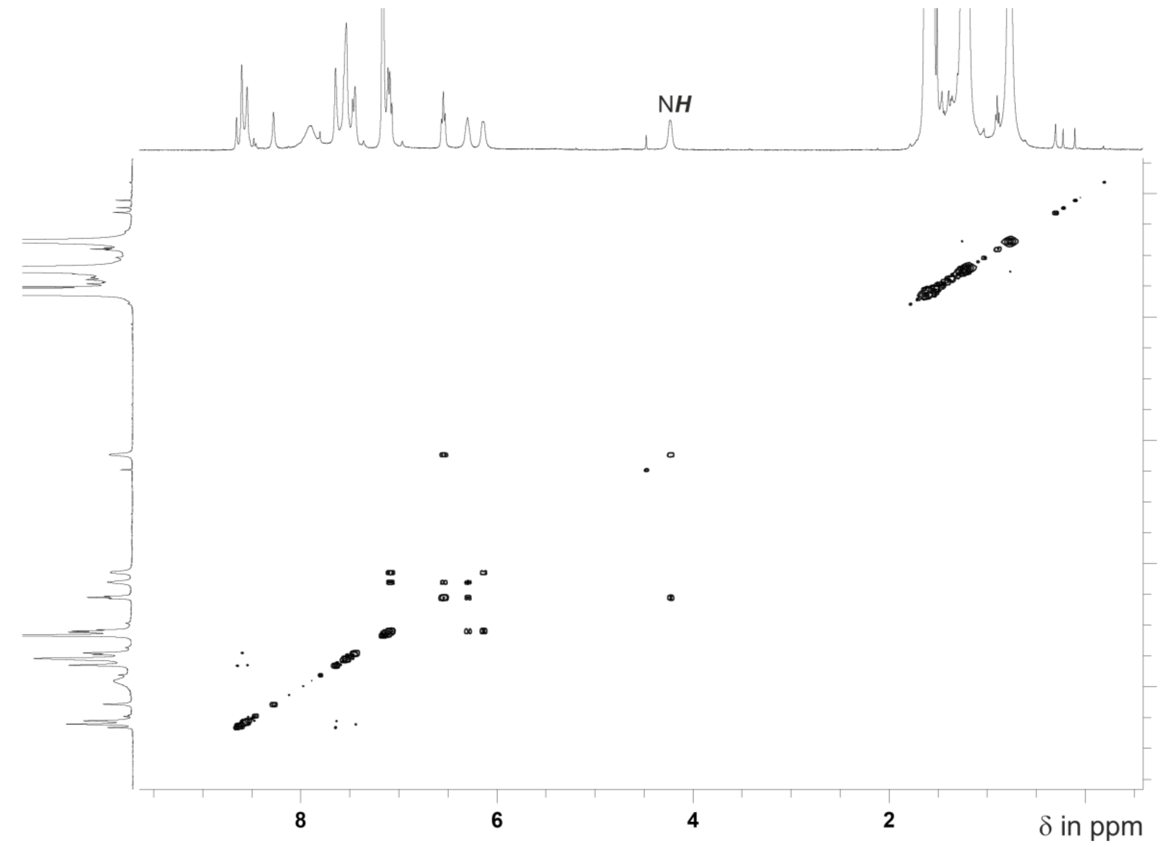

Figure S33: ^{1}H-^{1}H COSY NMR spectrum of reaction mixture with 0.89 eq. of azobenzene.

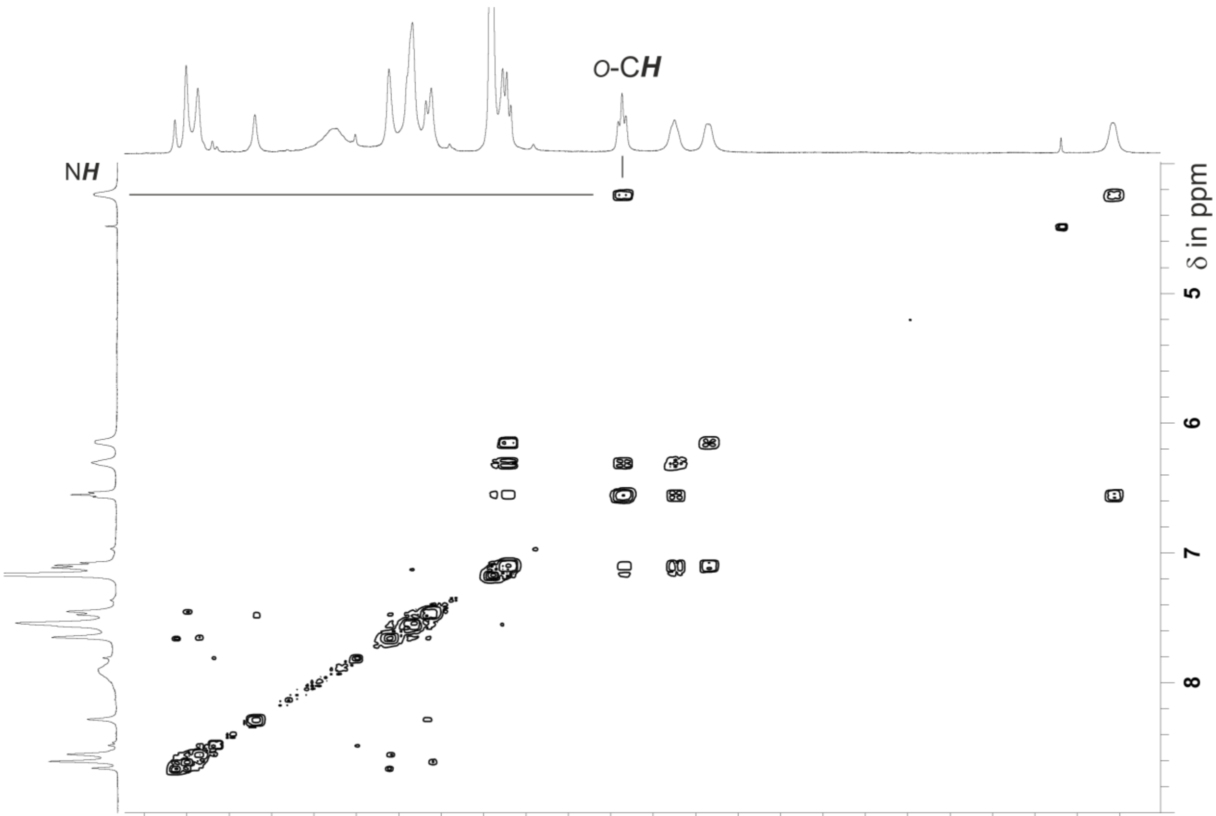

Figure S34: Section of the ^{1}H-^{1}H COSY NMR spectrum of reaction mixture with 0.89 eq. of azobenzene., important cross signal shown.

CW EPR measurements of azobenzenyl complex **5** were conducted in a toluene solution at ambient temperature. The EPR spectrum was obtained by the following experimental setup:

Sample: $1.22 \cdot 10^{-3}$ M solution in toluene

Temperature: ambient (ca. 293 K)

Frequency: 9.426323 GHz

Modulation amplitude: 0.75 G

Number of scans: 30

Determined *g*-value: 2.0023

The obtained experimental spectrum is shown in **Figure S35**. The simulated spectrum was obtained by iteratively simulate 4 hyperfine coupling constants to both adjacent nitrogen atoms and 2x2 and 2x1 hydrogen atoms of the phenyl rings as well as the line widths. The simulated hyperfine coupling constants in MHz of the azobenzenyl anion are visualized in **Figure S36**.

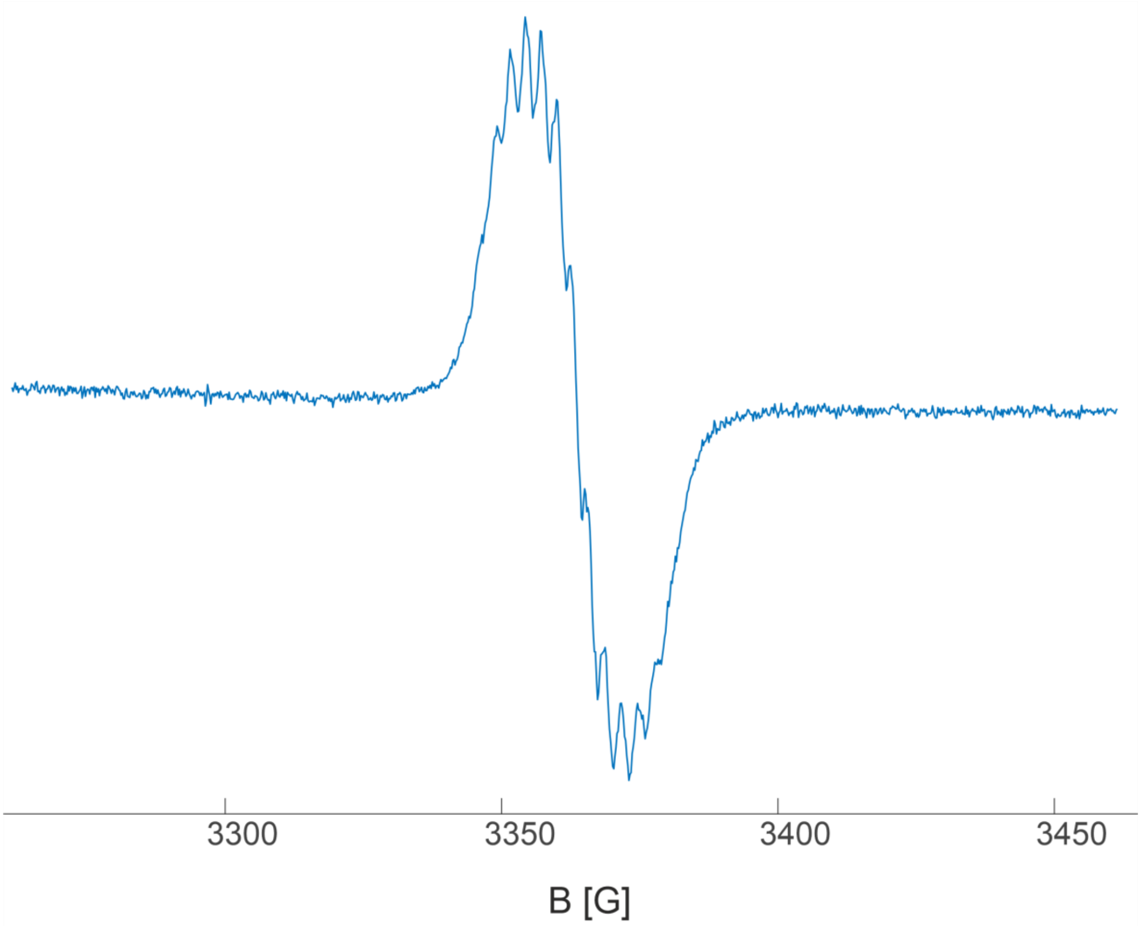

Figure S35: Experimental CW-EPR spectrum of **5**.

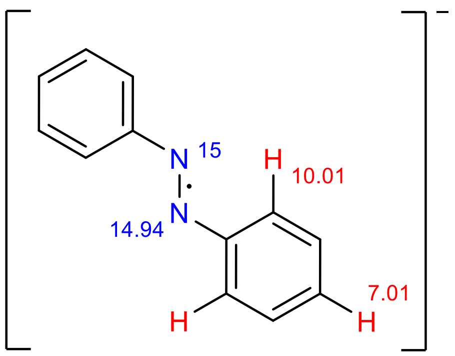

Figure S36: Chemical structure of the azobenzenyl radical anion in **5** with added hyperfine coupling constants to hydrogen atoms (red) and nitrogen atoms (blue) from the simulation.

2.6 [^{dtbp}CbzSr(CCSiMe$_3$)]$_2$ (6)

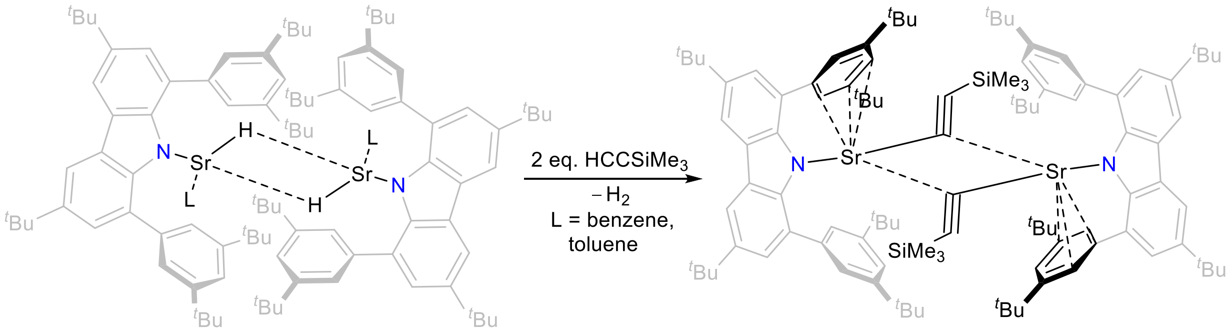

2 **6**

Freshly prepared hydride **2** (97 mg, 0.065 mmol) was suspended in toluene. To that suspension, a trimethylsilylacetylene solution in toluene (18 µL, 0.130 mmol, 2.00 eq.) was added dropwise. The formation of a clear solution is observed and the mixture was stirred at ambient temperature for an additional hour. The resulting toluene solution was concentrated at elevated temperatures and left undisturbed overnight. Within that night, yellow crystals were obtained. The supernatant was removed and the crystals dried briefly. Crystalline yield: 27 mg, 0.016 mmol, 25%.

^{1}H NMR (400 MHz, C_6D_6): δ(ppm) = -0.38 (s, 9 H, Si(C*H*$_3$)$_3$), 1.35 (s, 36 H, Ar-C(C*H*$_3$)$_3$), 1.58 (s, 18 H, Carb-C(C*H*$_3$)$_3$), 7.53 (t, 2 H, *p*-C*H*, $^4J_{HH}$ = 1.8 Hz), 7.66 (d, 2 H, $^4J_{HH}$ = 1.9 Hz, C^{2,7}*H*), 8.00 (d, 4 H, $^4J_{HH}$ = 1.8 Hz, *o*-C*H*), 8.53 (d, 2 H, $^4J_{HH}$ = 1.9 Hz, C^{4,5}*H*).

^{13}C{^{1}H} NMR (100.7 MHz, C_6D_6): δ(ppm) = 0.31 (s, Si(CH$_3$)$_3$), 31.95 (s, Ar-C(*C*H$_3$)$_3$), 32.63 (s, Carb-C(*C*H$_3$)$_3$), 35.01 (s, Carb-*C*(CH$_3$)$_3$), 35.48 (s, Ar-*C*(CH$_3$)$_3$), 116.63 (s, *C*^{4,5}), 122.14 (s, *p*-*C*H), 123.43 (s, *C*^{2,7}), 124.28 (s, *o*-*C*H), 126.93 (s, *C*^{4a,4b}), 127.05 (s, *C*^{1,8}), 130.50 (s, C*C*$_\beta$(SiMe$_3$)), 138.90 (s, *C*^{3,6}), 144.05 (s, *i*-*C*), 148.54 (s, *C*^{8a,9a}), 152.39 (s, *m*-*C*), 174.23 (s, *C*$_\alpha$C(SiMe$_3$)).

EA: no satisfactory analysis could be obtained.

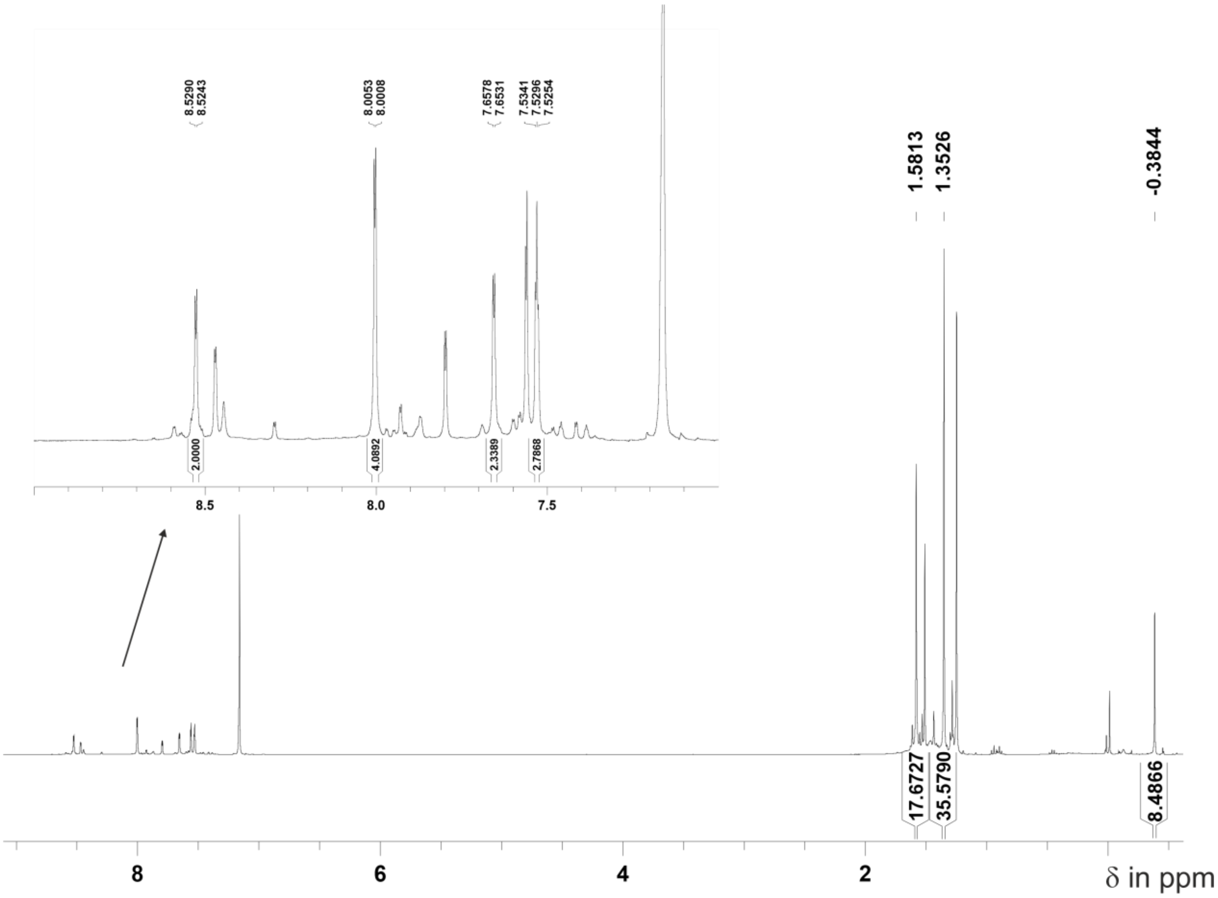

Figure S37: ^{1}H NMR spectrum of **6**.

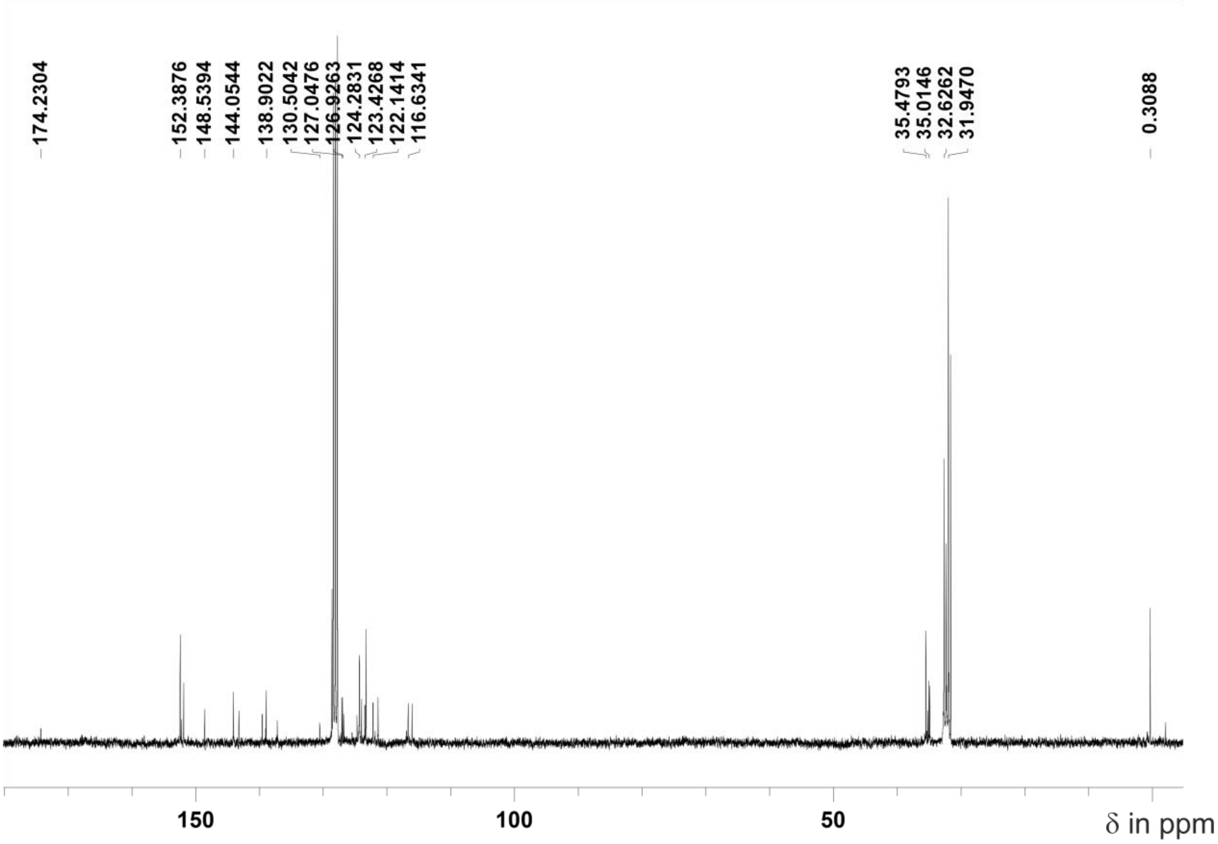

Figure S38: $^{13}C\{^{1}H\}$ NMR spectrum of **6**.

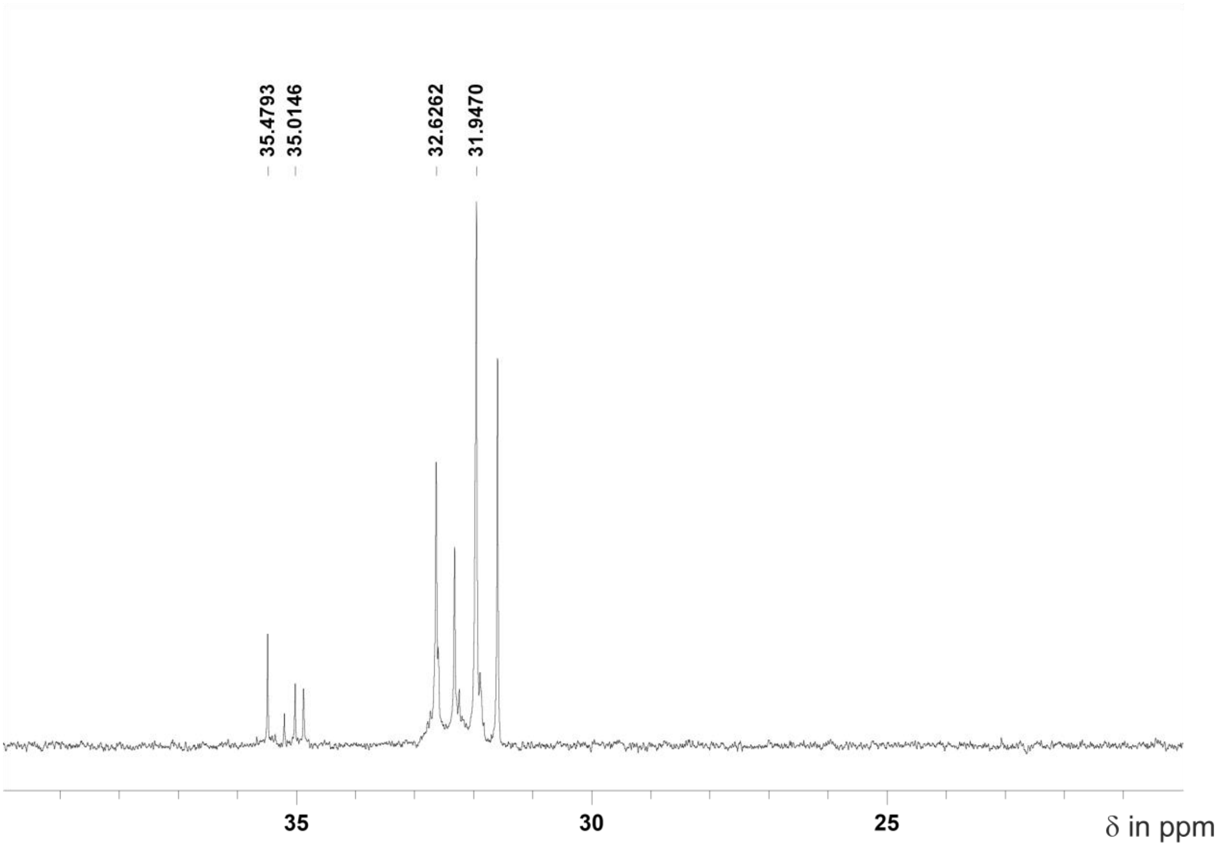

Figure S39: $^{13}C\{^{1}H\}$ NMR spectrum of **6** (20-40ppm).

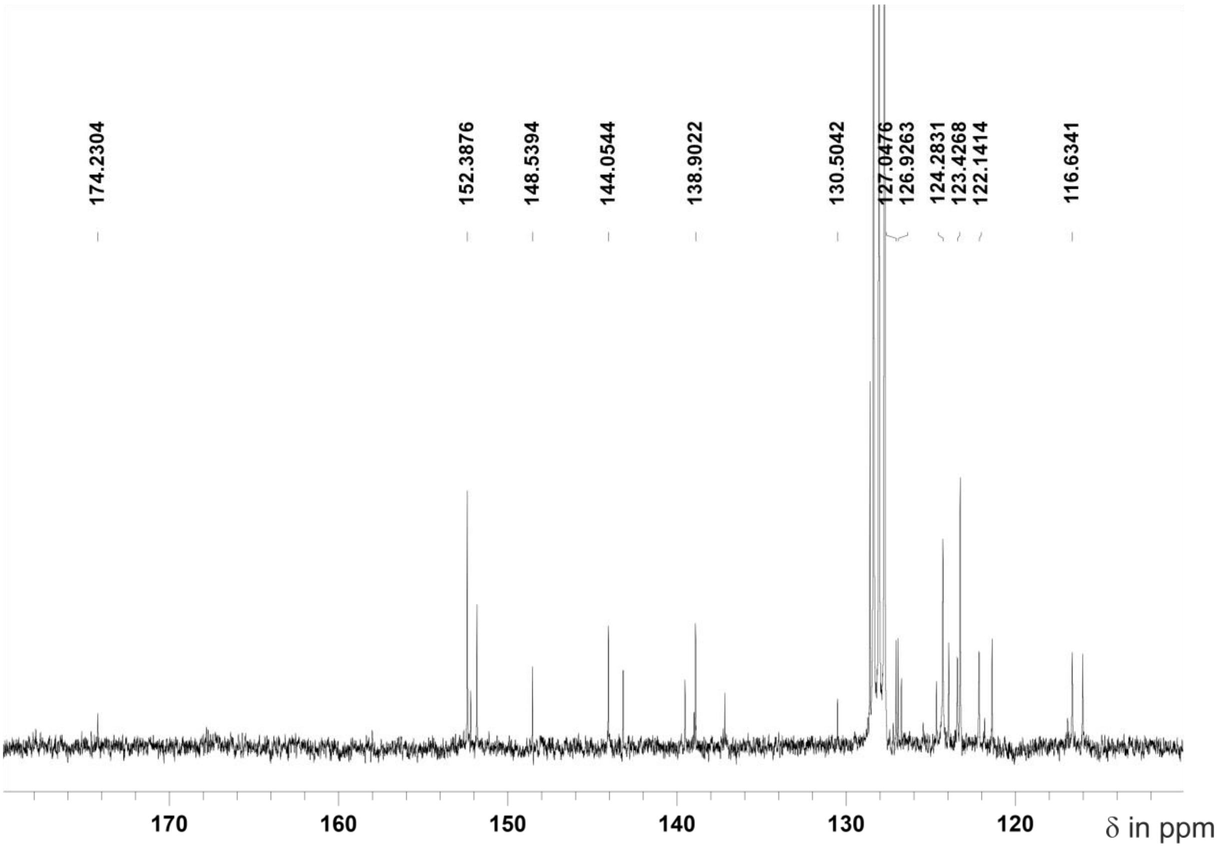

Figure S40: $^{13}C\{^{1}H\}$ NMR spectrum of **6** (110-180ppm).

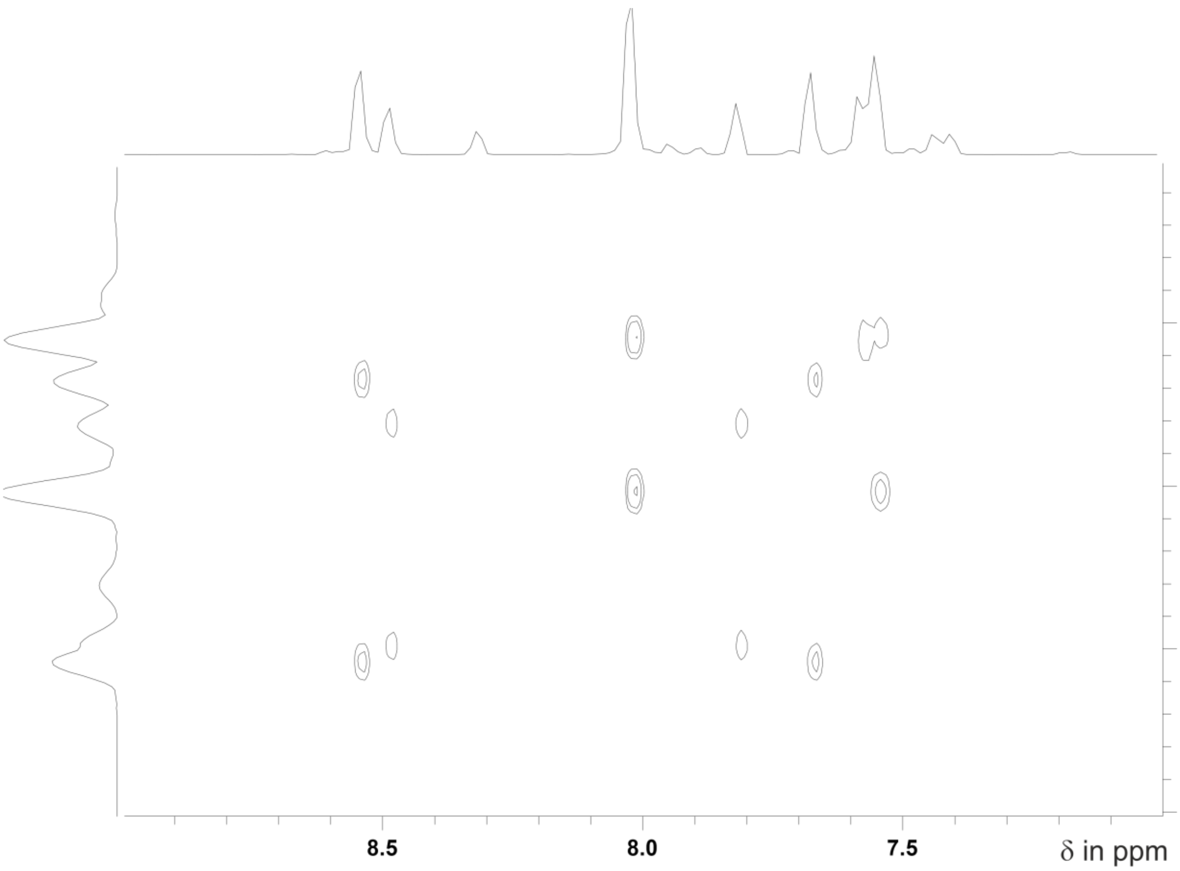

Figure S41: ^{1}H-^{1}H COSY NMR spectrum of **6** (relevant aromatic region).

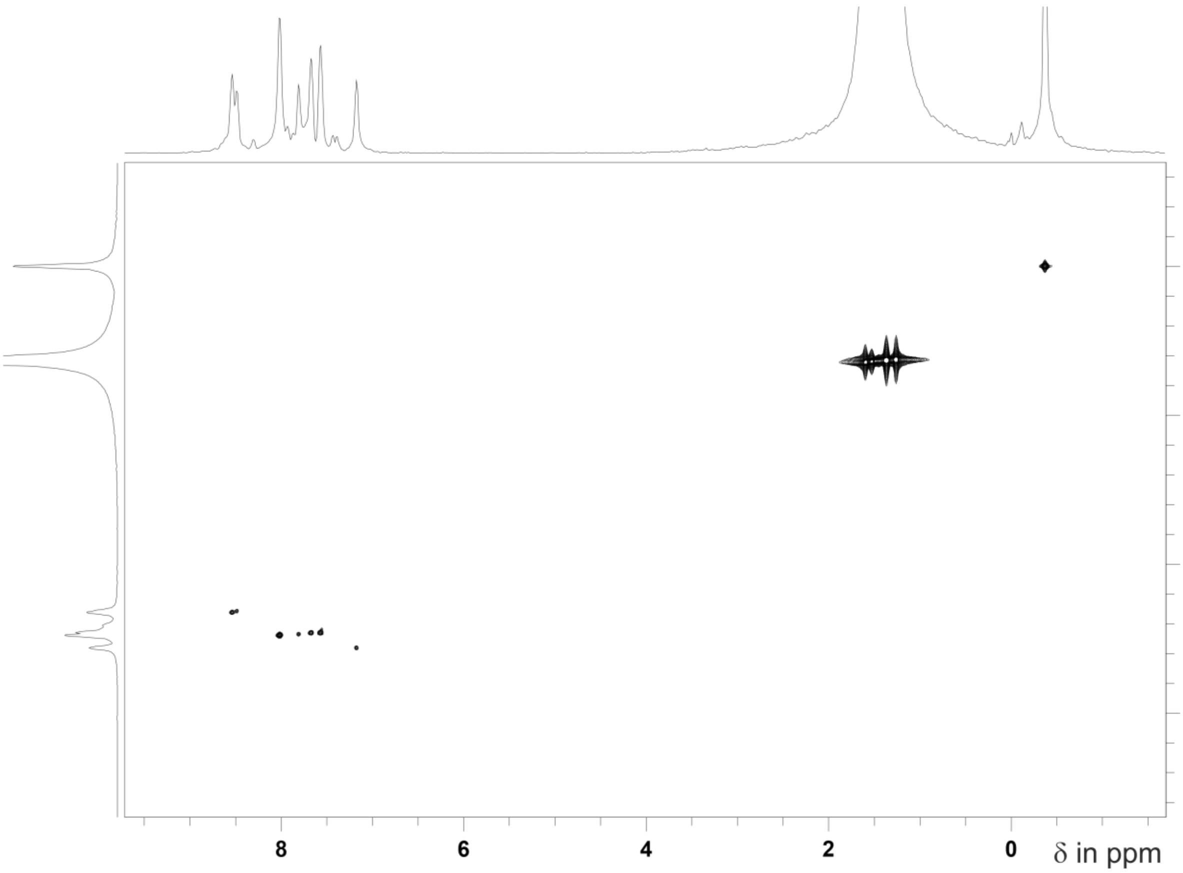

Figure S42: ^{1}H-^{13}C HMQC NMR spectrum of **6**.

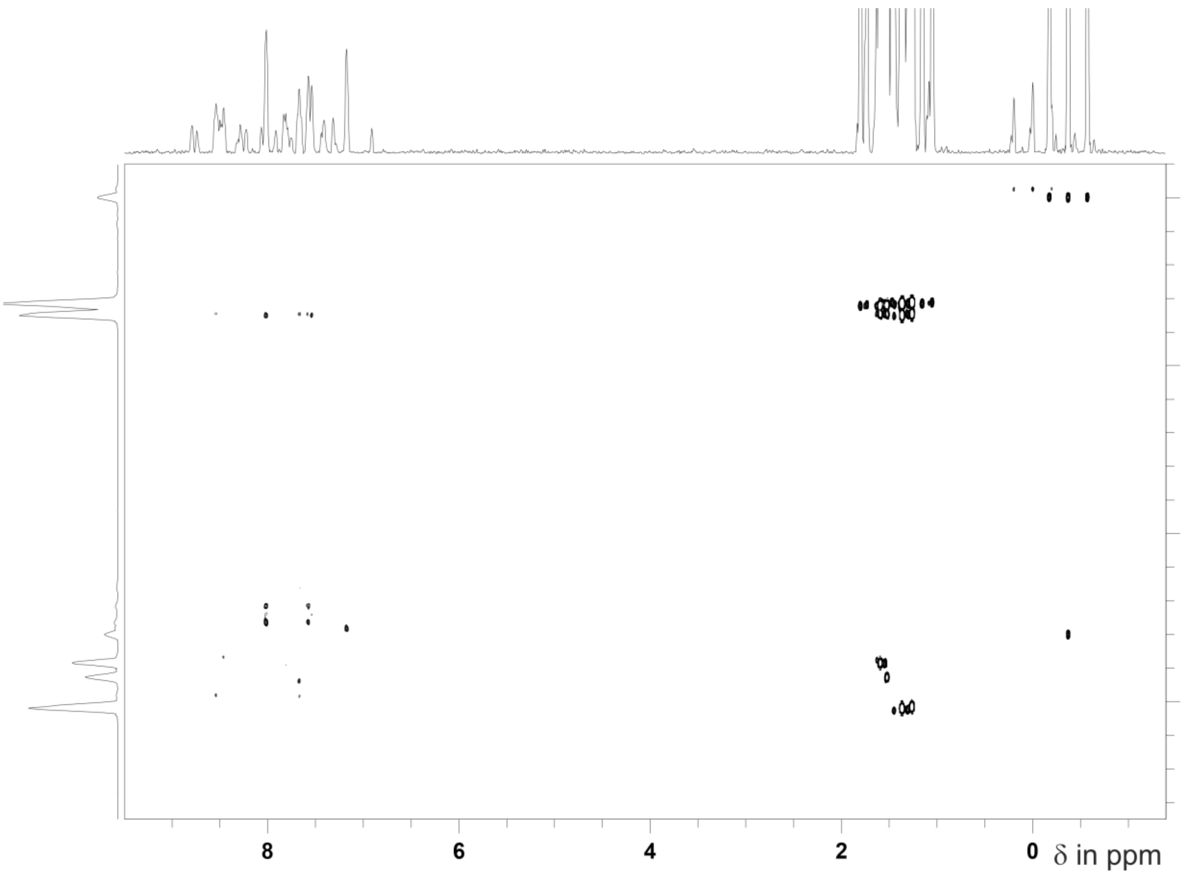

Figure S43: ^{1}H-^{13}C HMBC NMR spectrum of **6**.

2.7 $[^{dtbp}CbzSrD]_2$ (2-D)

The amide **1** (98 mg, 0.109 mmol) was dissolved in 0.9 mL of benzene. To that solution, phenylsilane-*d₃* (32 µL, 0.252 mmol, 2.31 eq.) was added via microsyringe. The mixture was shaken and then left undisturbed overnight. The deuteride **2-D** crystallized within one night. Afterwards, the supernatant was removed via syringe, the crystals were washed multiple times with *n*-hexane and briefly dried. Crystalline yield: 32 mg, 0.021 mmol, 39%. The characterisation data were obtained from the crystalline material.

The **^{1}H NMR** analysis shows a second species in solution, indicating insufficient stability in solution. This is recognized by a second signal set in addition to the expected signal set which matches the resonances of the hydride **2**. In some cases (e.g. 7.54-7.56ppm or 1.46ppm) the signals are overlapping which is why integration was not done. Additionally, the hydride signal is slightly observed, but with significantly less intensity.

^{2}H(D) NMR (400 MHz, C_6D_6): δ (ppm) = 1.30 (s, 1 H, Sr*D*).

IR (ATR): ῦ (cm^{-1}) = 378 (m), 398 (w), 407 (w), 417 (w), 427 (w), 438 (w), 448 (w), 456 (w), 465 (w), 485 (w), 502 (m), 519 (m), 544 (w), 560 (w), 580 (w), 590 (w), 598 (w), 608 (w), 618 (w), 645 (m), 676 (vs), 700 (vs), 714 (s), 730 (m), 761 (w), 774 (w), 824 (w), 846 (m), 868 (m), 899 (w), 924 (w), 934 (w), 965 (w), 985 (w), 1022 (w), 1035 (w), 1073 (w), 1135 (w), 1151 (w), 1182 (w), 1201 (w), 1233 (s), 1266 (m), 1284 (m), 1337 (w), 1361 (m), 1382 (w), 1392 (w), 1432 (w), 1461 (m), 1476 (m), 1588 (w), 2865 (w), 2903 (w), 2953 (m), 3034 (vw), 3065 (vw), 3090 (vw).

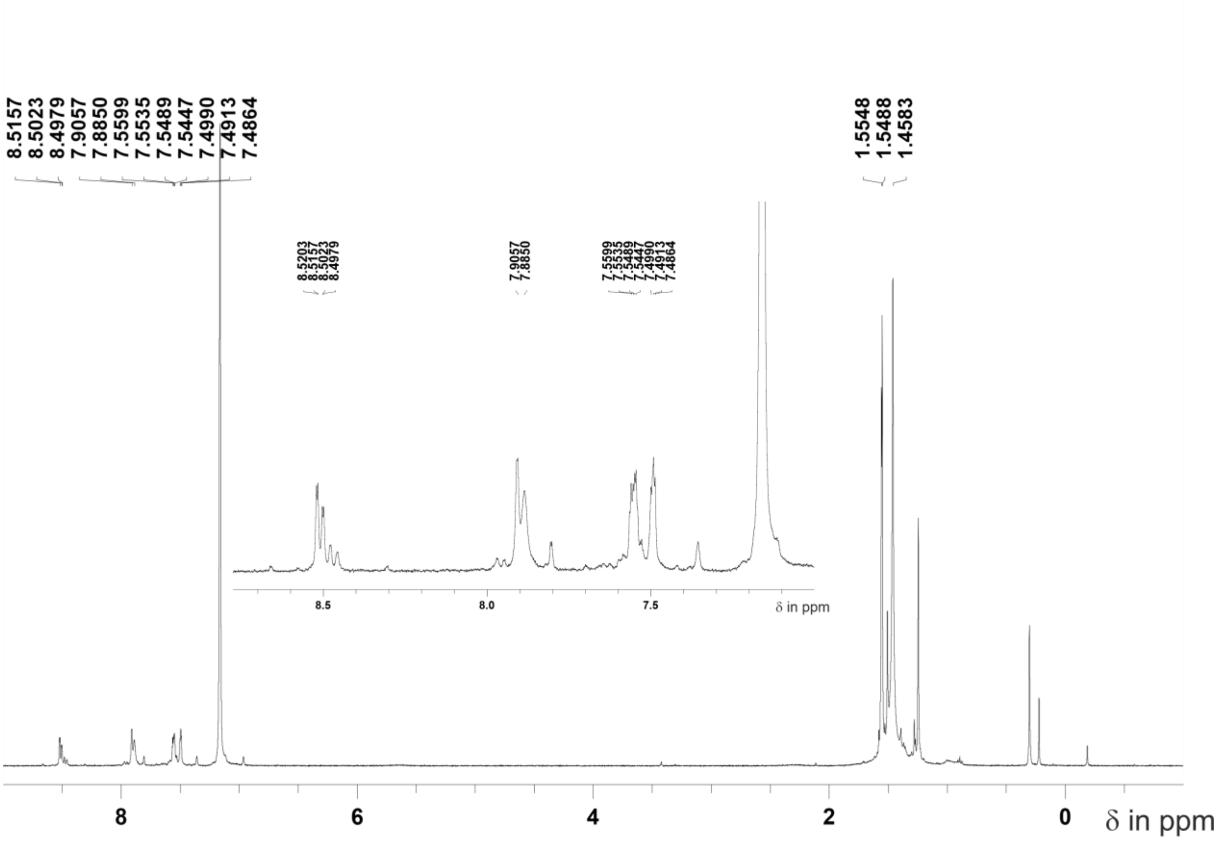

Figure S44: ^{1}H NMR spectrum of **2-D**, peaks picked from both species, integration was not performed due to overlapping signals.

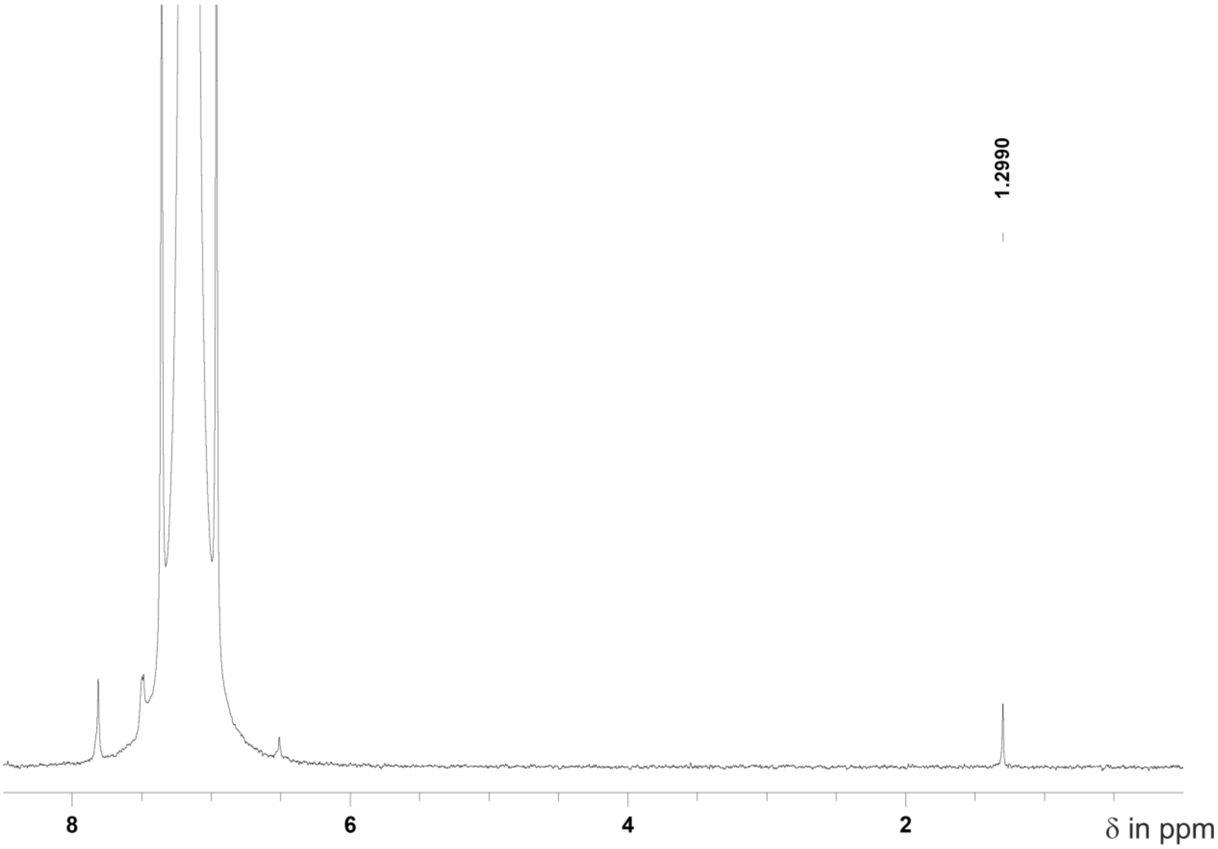

Figure S45: ^{2}H NMR spectrum of **2-D**.

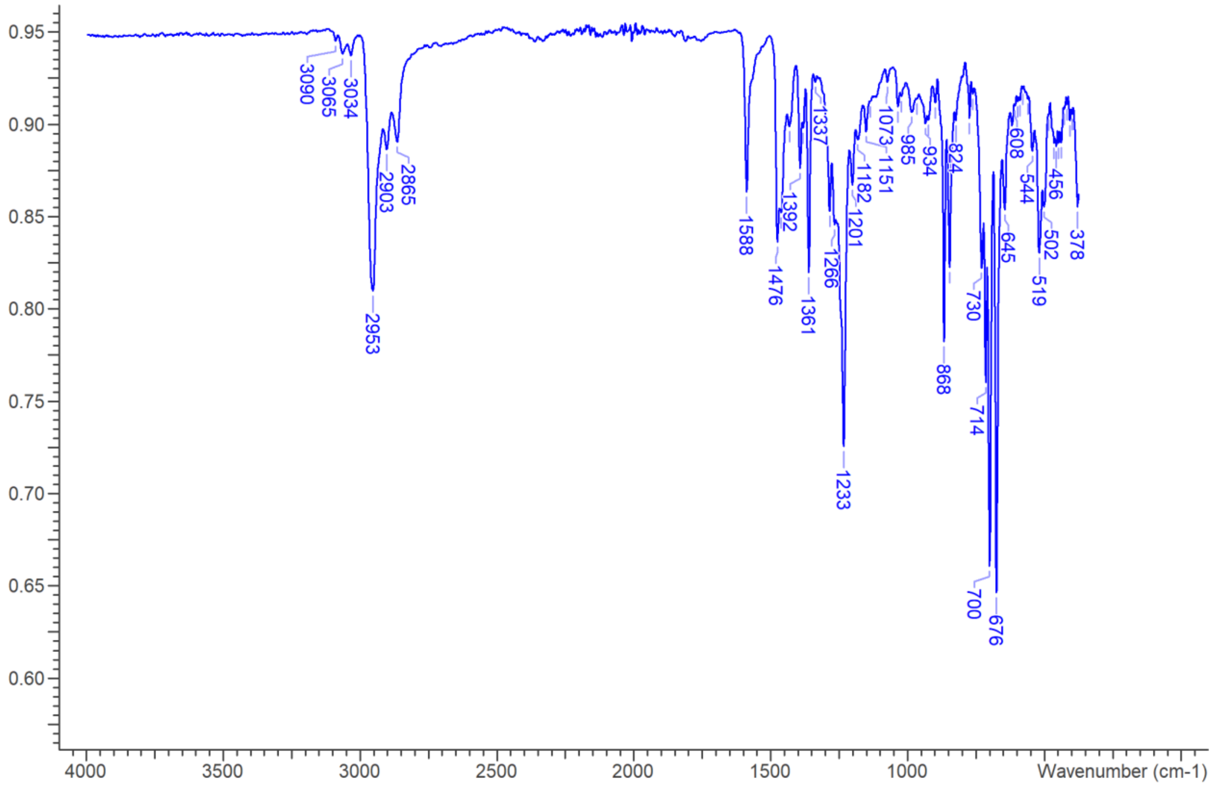

Figure S46: IR spectrum of **2-D**.

3 Crystallographic Details

Details of structure refinement:

1a: Three tertbutyl groups and four of the five co-crystallised solvent molecules were found to be disordered and modelled with split positions. The tertbutyl groups were split over two positions each (C41 0.64/0.36, C31 0.65/0.35, C27 0.95/0.05), the benzene molecules over two or three positions (C55 0.63/0.37, C61 0.77/0.23, C67 0.57/0.32/0.11, C73 0.77/0.23). For the disordered benzene molecules AFIX 66 was used to constrain the geometry as well as SIMU 0.01 0.02 and DELU 0.01 0.01 restraints were used. For the disordered tertbutyl groups, the first atoms (C27/C27B, C31/C31B, C41/C41B) were constrained with EADP, then for all other atoms SIMU 0.01 0.02 and DELU 0.01 0.01 restraints were used. The geometry of both parts was restrained with SAME 0.01.

1b: The N(SiMe3)2 moiety was found to be disordered and modelled with two split positions (0.42/0.58). One tertbutyl group was disordered and modelled with two split positions (0.57/0.43). For both, the first atoms (N2/N2B, C27/C27B) were constrained with EADP, then for all other atoms SIMU 0.01 0.02 and DELU 0.01 0.01 restraints were used. The geometry of both parts was restrained with SAME 0.01.

2a: Eight tertbutyl groups were found to be disordered and modelled with split positions. The tertbutyl groups were split over two or three positions each (C31 0.88/0.12, C41 0.89/0.11, C45 0.75/0.25, C67 0.78/0.22, C71 0.67/0.33, C75 0.70/0.30, C89 0.54/0.46, C93 0.50/0.50). For the disordered tertbutyl groups, the first atoms (C31/C31B, …) were constrained with EADP, then for all other atoms SIMU 0.01 0.02 and DELU 0.01 0.01 restraints were used. The geometry of both parts was restrained with SAME 0.01. The strontium atoms were split over two positions each (0.65/0.35). One coordinated benzene molecule was split over two positions (0.71/0.29), the geometry of both parts was restrained with SAME 0.01 and SIMU 0.01 0.02 and DELU 0.01 0.01 restraints were used.

2b: Seven tertbutyl groups were found to be disordered and modelled with split positions. The tertbutyl groups were split over two or three positions each (C19 0.42/0.32/0.25, C27 0.57/0.43, C31 0.76/0.24 C41 0.66/0.34, C78 0.79/0.21, C82 0.81/0.19, C86 0.54/0.46, C100 0.57/0.43). For the disordered tertbutyl groups, the first atoms (C19/C19B/C19C, …) were constrained with EADP, then for all other atoms SIMU 0.01 0.02 and DELU 0.01 0.01 restraints were used. The geometry of both parts was restrained with SAME 0.01. The strontium atoms were split over two positions each (0.96/0.04) and constrained with EADP. SADI 0.001 was used to restrain the Sr-H contacts for the split Sr_2H_2 unit. SQUEEZE was used to remove disordered solvent density of 205 e per cell.

3: Four tertbutyl groups were found to be disordered and modelled with split positions. The tertbutyl groups were split over two positions each (C19 0.70/0.30, C41 0.55/0.45, C71 0.76/0.24 C89 0.62/0.38). For the disordered tertbutyl groups, the first atoms (C19/C19B, …) were constrained with EADP, then for all other atoms SIMU 0.01 0.02 and DELU 0.01 0.01 restraints were used. The geometry of both parts was restrained with SAME 0.01. SQUEEZE was used to remove disordered solvent density of 124 e per cell.

4: Three tertbutyl groups and two of the five co-crystallised solvent molecules were found to be disordered and modelled with split positions. The tertbutyl groups were split over two positions each (C23 0.63/0.37, C45 0.58/0.42, C73 0.94/0.06), the toluene molecules over two positions (C99 0.51/0.49, C120 0.57/0.43). For the disordered toluene molecules AFIX 66 and SAME 0.01 were used to constrain the geometry as well as SIMU 0.01 0.02 and DELU 0.01 0.01 restraints for the ellipsoids. For the disordered tertbutyl groups, the first atoms (C23/C23B, C45/C45B, C73/C73B) were constrained with EADP, then for all other atoms SIMU 0.01 0.02 and DELU 0.01 0.01 restraints were used. The geometry of both parts was restrained with SAME 0.01.

5: One tertbutyl group was found to be disordered and modelled with split positions (C41 0.73/0.27). The first atoms (C41/C41B) were constrained with EADP, then for all other atoms SIMU 0.01 0.02 and DELU 0.01 0.01 restraints were used. The geometry of both parts was restrained with SAME 0.01.

6: Two tertbutyl groups and the co-crystallised toluene molecules were found to be disordered and modelled with split positions. The tertbutyl groups were split over two positions each (C24 0.71/0.29, C46 0.66/0.34), the toluene molecules over two positions (0.69/0.31). For the disordered toluene molecules AFIX 66 and SAME 0.01 were used to constrain the geometry as well as SIMU 0.01 0.02 and DELU 0.01 0.01 restraints for the ellipsoids. For the disordered tertbutyl groups, the first atoms (C24/C24B, C46/C46B) were constrained with EADP, then for all other atoms SIMU 0.01 0.02 and DELU 0.01 0.01 restraints were used. The geometry of both parts was restrained with SAME 0.01.

B: Three tertbutyl groups and three of the eight the co-crystallised benzene molecules were found to be disordered and modelled with split positions. The tertbutyl groups were split over two positions each (C35 0.64/0.36, C53 0.83/0.17, C101 0.70/0.30), the benzene molecules were split over two positions (C127 0.57/0.43, C133 0.74/0.26, C151 0.53/0.47). For the disordered benzene molecules AFIX 66 and SAME 0.01 0.02 were used to constrain the geometry as well as SIMU 0.01 0.02 restraints for the ellipsoids. For three of the non-disordered benzene molecules, AFIX 66 was used. For the disordered tertbutyl groups, the first atoms (C35/C35BB, …) were constrained with EADP, then for all other atoms SIMU 0.01 0.02 restraints were used. The geometry of both parts was restrained with SAME 0.01. For C54B, ISOR 0.005 0.001 was used. The crystal was refined as non-merohedral two-domain twin (0.53/0.47) vs. HKL5 data.

Table S1: Crystallographic details for **1a**, **1b** and **2**·benzene.

	[^{dtbp}CbzSrN(SiMe$_3$)$_2$] (**1a**)	[^{dtbp}CbzSrN(SiMe$_3$)$_2$]$_2$ (**1b**)	^{dtbp}CbzSrH·benzene (**2a**)
CCDC#	2145433	2145434	2145440
Empirical formula	$C_{81}H_{109}N_2Si_2Sr$	$C_{54}H_{82}N_2Si_2Sr$	$C_{63}H_{80}NSr$
FW [g mol^{-1}]	1254.50	903.01	938.90
Wavelength [Å]	0.71073	1.34143	1.34143
Temperature [K]	200(2)	150(2)	150(2)
Crystal system	monoclinic	triclinic	monoclinic
Space group	$P2_1/n$	$P\bar{1}$	$P2_1/c$
a [Å]	13.8836(3)	12.5734(6)	15.9544(7)
b [Å]	19.8053(3)	14.6523(9)	28.6490(6)
c [Å]	27.8348(6)	16.0992(7)	25.2050(11)
α [°]	90	64.730(4)	90
β [°]	101.851(2)	79.464(3)	104.834(3)
γ [°]	90	79.177(4)	90
V [Å^3]	7490.6(3)	2616.7(2)	11136.7(7)
Z	4	2	8
ρ_{calc} [g cm^{-3}]	1.112	1.146	1.120
μ	0.793	1.393	1.062
F(000)	2700	972	4024
reflections collected	64650	31538	69974
independent reflections	19883	10740	24380
reflectionsGT(I>2σ(I))	13569	7937	17679
R_{int}	0.0377	0.0320	0.0230
Parameters	1086	672	1602
Restraints	1801	490	1852
GooF	1.058	0.988	1.026
R1	0.0538	0.0448	0.0490
R1 (all)	0.0943	0.0631	0.0695
wR2	0.1110	0.1211	0.1357
wR2 (all)	0.1323	0.1272	0.1447
weight factors	0.0454 / 5.5638	0.0843 / 0	0.0761 / 4.8885

Table S2: Crystallographic details for **2**·toluene,**3** and **4**.

	^{dtbp}CbzSrH·toluene (**2b**)	(^{dtbp}Cbz)$_2$Sr (**3**)	[^{dtbp}CbzSr(OCH)]$_2$ (**4**)
CCDC#	2145439	2145441	2385469
Empirical formula	$C_{127.50}H_{166}N_2Sr_2$	$C_{102}H_{142}N_2Sr$	$C_{63}H_{81}NOSr$
FW [g mol^{-1}]	1901.85	1483.79	955.90
Wavelength [Å]	0.71073	0.71073	1.34143
Temperature [K]	200(2)	200(2)	150(2)
Crystal system	triclinic	monoclinic	triclinic
Space group	$P\bar{1}$	$P2_1$/n	$P\bar{1}$
a [Å]	15.9716(9)	23.7938(8)	15.7030(8)
b [Å]	16.8511(9)	16.4259(4)	16.3140(10)
c [Å]	21.9341(10)	25.5830(8)	22.8463(14)
α [°]	83.745(4)	90	82.007(5)
β [°]	75.838(4)	110.783(3)	82.007(4)
γ [°]	82.605(4)	90	70.494(5)
V [Å^3]	5657.8(5)	9348.1(5)	5436.3(6)
Z	2	4	4
ρ_{calc} [g cm^{-3}]	1.116	1.054	1.168
μ	0.990	0.620	1.102
F(000)	2042	3224	2048
reflections collected	41309	52887	60407
independent reflections	22028	18356	21802
reflectionsGT(I>2σ(I))	12583	12354	16950
R_{int}	0.0541	0.0448	0.0283
Parameters	1439	1064	1451
Restraints	1340	636	1488
GooF	1.018	1.052	1.063
R1	0.0594	0.0612	0.0471
R1 (all)	0.1193	0.1013	0.0614
wR2	0.1364	0.1363	0.1225
wR2 (all)	0.1663	0.1563	0.1264
weight factors	0.0748 / 1.8255	0.0476 / 13.2074	0.0436 / 8.169203

Table S3: Crystallographic details for **5** and **6**.

	[^{dtbp}CbzSr(PhNNPh)]$_2$ (**5**)	[^{dtbp}CbzSr(CCSiMe$_3$)]$_2$ (**6**)	[(^{dtbp}CbzSr)$_2$(N$_2$Ph$_2$)] (**B**)
CCDC#	2385470	2385471	2403487
Empirical formula	$C_{67}H_{82}N_3Sr$	$C_{60}H_{81}NSiSr$	$C_{156}H_{186}N_4Sr_2$
FW [g mol^{-1}]	1016.97	931.96	2292.32
Wavelength [Å]	0.71073	0.71073	0.71073
Temperature [K]	100(2)	100(2)	100(2)
Crystal system	monoclinic	triclinic	monoclinic
Space group	*Cc*	$P\bar{1}$	P 2$_1$
a [Å]	22.5852(6)	13.455(3)	15.3524(3)
b [Å]	18.8182(4)	14.395(3)	27.5497(5)
c [Å]	27.9700(8)	15.382(4)	15.8014(3)
α [°]	90	115.702(16)	90
β [°]	105.864(2)	90.42(2)	93.272(2)
γ [°]	90	101.619(18)	90
V [Å^3]	11434.8(5)	2614.3(11)	6672.4(2)
Z	8	2	2
ρ_{calc} [g cm^{-3}]	1.181	1.184	1.141
μ	0.985	1.092	0.851
F(000)	4344	1000	2452
reflections collected	49542	24599	133223
independent reflections	19956	10162	*
reflectionsGT(I>2σ(I))	16581	7616	83894
R_{int}	0.0324	0.0442	0.1220
Parameters	1352	699	1655
Restraints	158	741	1357
GooF	1.059	1.015	0.938
R1	0.0409	0.0571	0.0554
R1 (all)	0.0584	0.0866	0.1075
wR2	0.0912	0.1253	0.0930
wR2 (all)	0.1001	0.1420	0.1057
weight factors	0.0449 / 15.5106	0.0566 / 5.0562	0.0432 / 0

*twin data processing

4 Computational Details

All computations were performed using Gaussian16[10] utilizing the PBE1PBE level of theory, Def2SVP basis sets and empirical dispersion correction (GD3BJ). No solvent corrections were applied. All optimized molecular structures where checked to be minima on the energy hypersurface and possess no imaginary vibrational frequencies. The vibrational frequencies are not scaled.

4.1 Vibrations of the Sr_2H_2 core

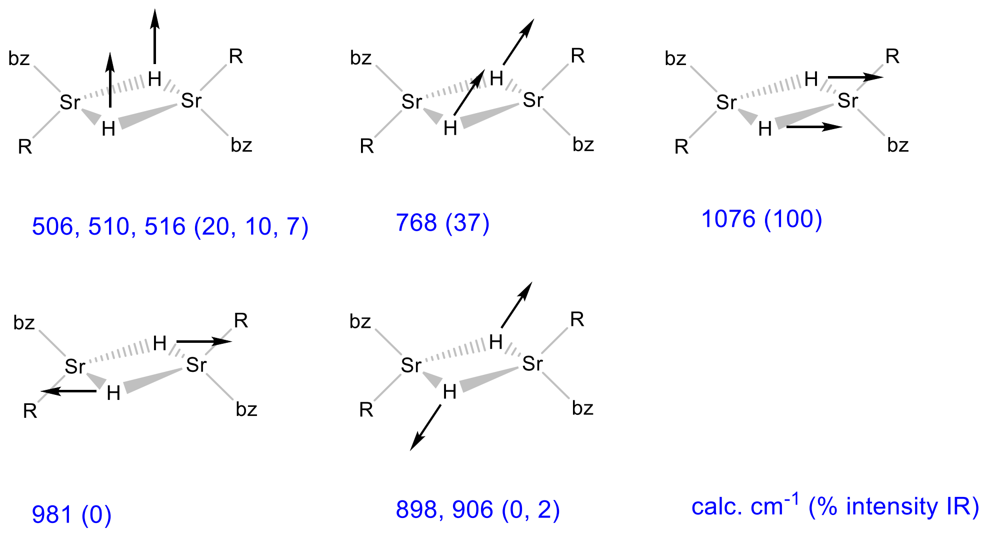

Figure S47: Vibrational modes involving the Sr_2H_2 core of **2**, their calculated unscaled frequencies and intensities.

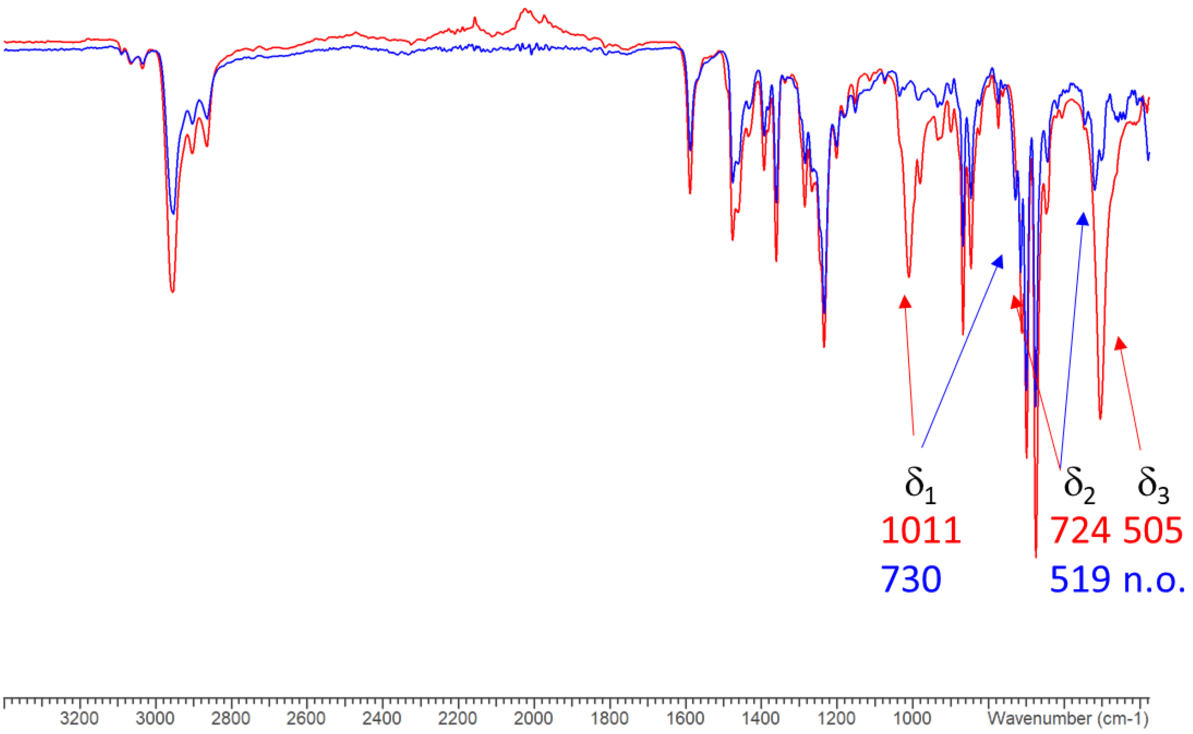

Figure S48: Superimposition of IR spectra of $[CbzSr(C_6H_6)H]_2$ (red) and $[CbzSr(C_6H_6)D]_2$ (blue).

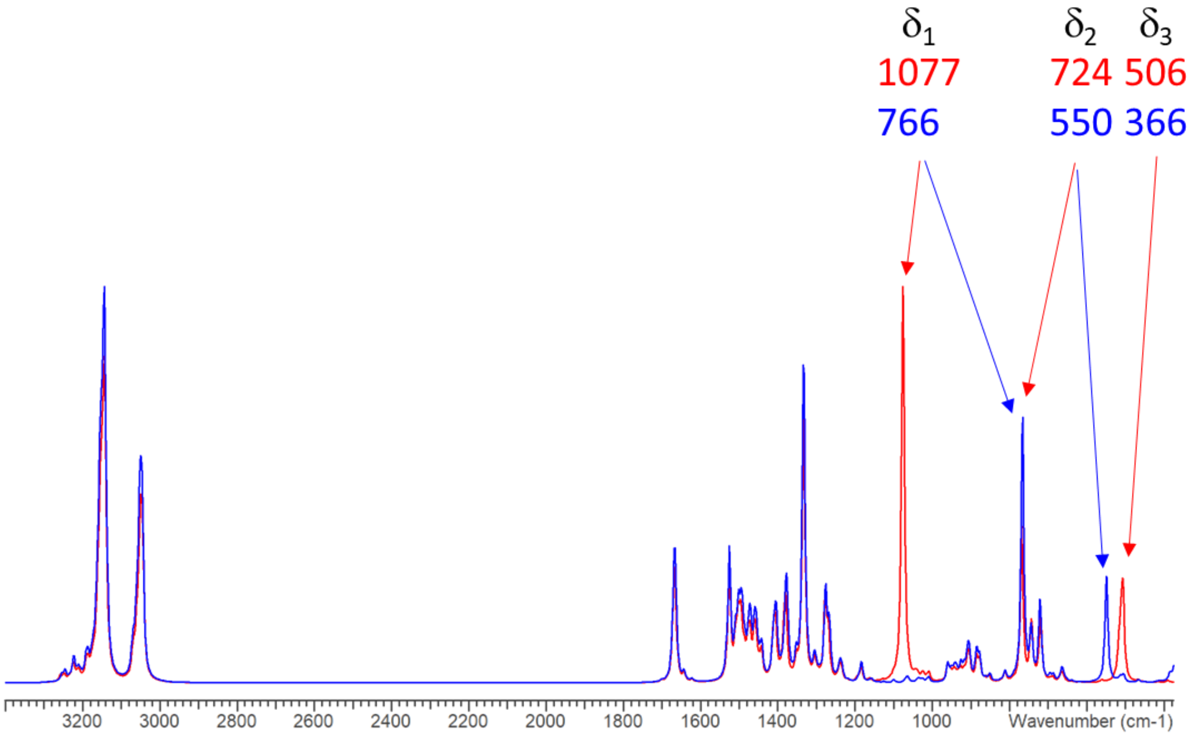

Figure S49: Superimposition of predicted IR spectra of $[CbzSr(C_6H_6)H]_2$ (red) and $[CbzSr(C_6H_6)D]_2$ (blue).

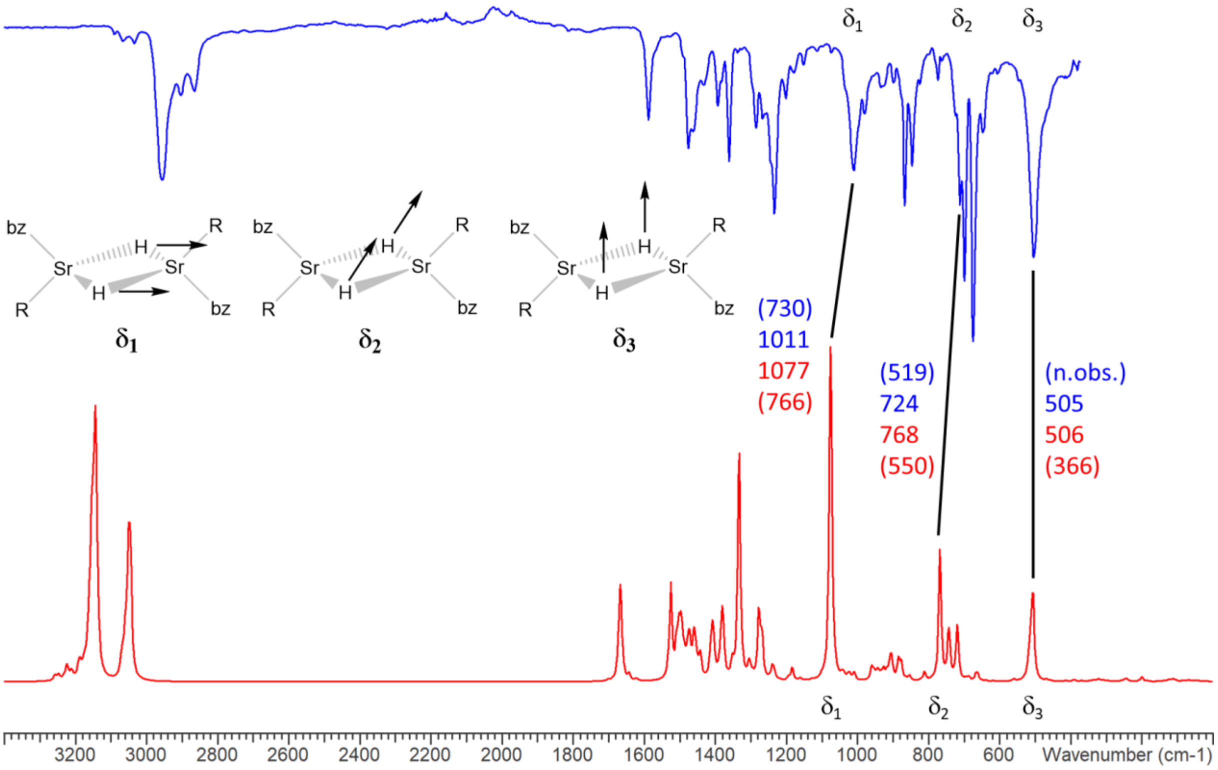

Figure S50: Superimposition of measured (blue) and predicted (red) IR spectra of $[CbzSr(C_6H_6)H]_2$, values for $[CbzSr(C_6H_6)D]_2$ in brackets.

4.2 Radical Ligand

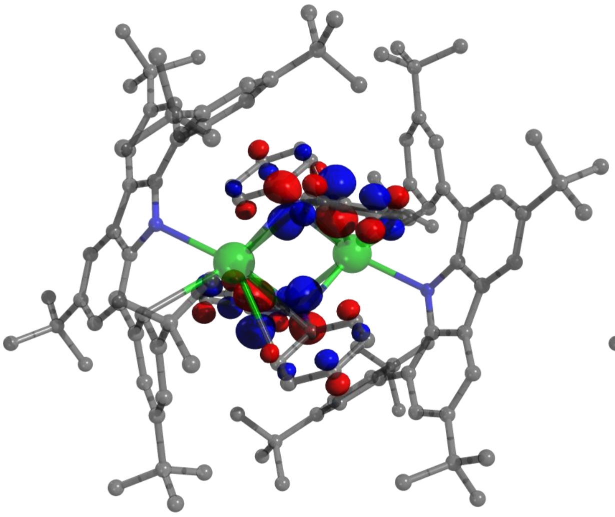

-4.649 eV

SOMO2

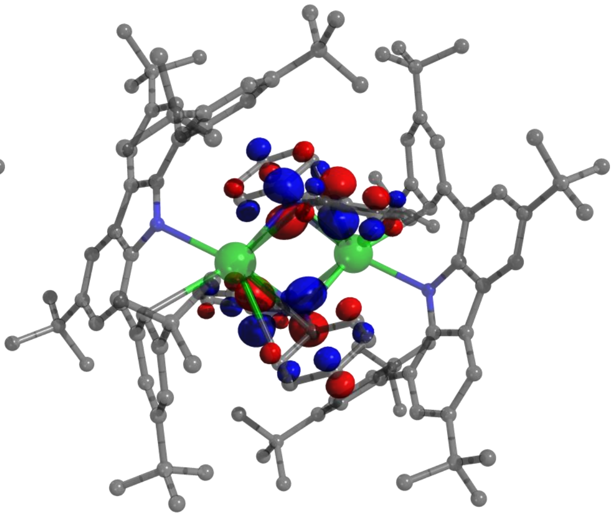

-4.865 eV

SOMO1

Figure S51: SOMOs of dimeric **5**.

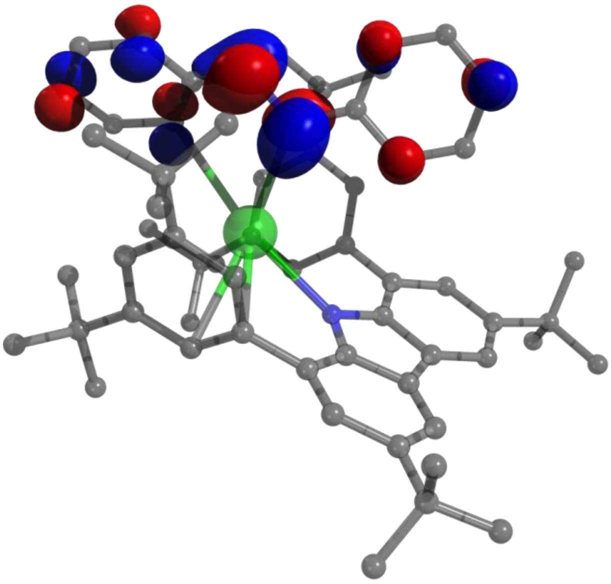

Figure S52: SOMO of monomeric **5**.

Table S4: Computed EPR parameters for monomeric and dimeric **5** (A in MHz).

Monomer

g_{iso}	1.99969		
A_{iso}(N1)	29.0	A_{iso}(H5)	-7.5
A_{iso}(N2)	33.1	A_{iso}(H6)	+3.9
A_{iso}(H1)	-8.9	A_{iso}(H7)	-9.0
A_{iso}(H2)	-9.8	A_{iso}(H8)	+4.1
A_{iso}(H3)	-5.9	A_{iso}(H9)	-7.7
A_{iso}(H4)	+3.1	A_{iso}(H10)	-10.5

Dimer

g_{iso}	1.99410		
A_{iso}(N1)	14.8	A_{iso}(H5)	-4.5
A_{iso}(N2)	16.3	A_{iso}(H6)	-4.0
A_{iso}(H1)	-4.8	A_{iso}(H7)	-1.7
A_{iso}(H2)	2.5	A_{iso}(H8)	-4.6
A_{iso}(H3)	-5.2	A_{iso}(H9)	2.2
A_{iso}(H4)	2.1	A_{iso}(H10)	-4.7

4.3 Optimised Structures

4.3.1 Compound 2

0 1

Sr	1.41779200	-1.10874800	0.22277500
H	0.75237500	1.10251600	0.02427700
N	2.88056300	-1.49198000	-1.77067400
C	3.39162300	-0.40774900	-2.44042200
C	4.21631300	0.62257700	-1.93825200
C	4.51156200	1.69088800	-2.79037200
H	5.15459400	2.47468900	-2.38734000
C	4.05186900	1.78513300	-4.12024300
C	3.25732400	0.74610700	-4.61001000
H	2.87548800	0.77503600	-5.63478700
C	2.92401300	-0.33277400	-3.78888400
C	2.05579000	-1.46969000	-3.95007700
C	1.23678800	-1.92821900	-4.98172200
H	1.24172600	-1.40184900	-5.94031900
C	0.39519500	-3.02930800	-4.78482700
C	0.40929600	-3.65370800	-3.52063000
H	-0.22756600	-4.52075800	-3.33961900
C	1.23559400	-3.24496100	-2.46744800
C	2.07535000	-2.12761100	-2.68166300
C	4.79797500	0.60887600	-0.57109300
C	5.65544000	-0.42839300	-0.17114200
H	5.79479400	-1.26360700	-0.85751100
C	6.36212700	-0.35597000	1.03262000
C	6.13536100	0.75123300	1.86475200
H	6.68575900	0.81774000	2.80514300
C	5.26186200	1.78716000	1.52227400
C	4.60355400	1.69698900	0.28877400
H	3.93163900	2.48692000	-0.04174700
C	7.40841900	-1.39709700	1.44977900
C	7.61703800	-2.46250200	0.37260500
H	6.69201300	-3.01156700	0.15272800
H	8.36662400	-3.19484600	0.71019200
H	7.98120500	-2.01935400	-0.56663400
C	6.96480200	-2.08275000	2.75079300
H	6.81817700	-1.35711700	3.56524800
H	7.72230900	-2.81076100	3.08313400
H	6.01939900	-2.62701000	2.60871900
C	8.75487400	-0.69255500	1.68317400
H	8.70120400	0.04627700	2.49633600
H	9.08407500	-0.16748400	0.77337700
H	9.52889200	-1.42858600	1.95382300
C	5.13390700	3.02385200	2.42180400
C	5.10257800	2.64147900	3.90829400
H	4.98099500	3.54430300	4.52693400
H	6.03104200	2.14982300	4.23275300
H	4.26649100	1.96467700	4.13982400
C	6.35906500	3.91698800	2.16237600
H	6.31524300	4.82813800	2.78126000
H	6.40517700	4.22009600	1.10502300
H	7.29350300	3.38557700	2.39919600
C	3.87075500	3.83023300	2.10540000
H	2.95433000	3.22840000	2.21399500
H	3.88884900	4.23374100	1.08388900
H	3.79029700	4.68754300	2.79111400
C	4.39409900	2.97231600	-5.02981500
C	5.29251000	3.99880200	-4.33586100
H	4.80996900	4.42989200	-3.44536500
H	5.51471400	4.82803300	-5.02546800
H	6.25167900	3.55757100	-4.02470000
C	3.09572800	3.67928300	-5.45117100
H	2.55786500	4.06306000	-4.57085600
H	2.41764000	2.99807700	-5.98691700
H	3.31173200	4.53075800	-6.11752700
C	5.12843000	2.46637600	-6.28098300
H	5.38075500	3.30511100	-6.95058100

H	4.51539100	1.75447300	-6.85339400
H	6.06255800	1.95379900	-6.00399900
C	-0.51760200	-3.50046600	-5.92492300
C	-1.55951700	-2.40954000	-6.22114000
H	-2.19685500	-2.22644800	-5.34193300
H	-2.21303600	-2.70811000	-7.05746200
H	-1.08186400	-1.45478400	-6.48833400
C	0.31904800	-3.76147700	-7.18662700
H	0.83841300	-2.85532500	-7.53135000
H	-0.32384700	-4.10938400	-8.01158800
H	1.08210300	-4.53191400	-6.99649900
C	-1.26104300	-4.79242600	-5.57795000
H	-1.92809200	-4.66110200	-4.71353100
H	-0.56409700	-5.61418300	-5.35249600
H	-1.88443600	-5.10511200	-6.43008900
C	1.22333600	-3.97088400	-1.17239800
C	0.01976500	-4.22512100	-0.50174400
H	-0.89726800	-3.82150500	-0.93217500
C	-0.01729500	-4.96039700	0.69235600
C	1.19263600	-5.44125700	1.20078600
H	1.18164400	-6.02633100	2.11833100
C	2.42119000	-5.20373600	0.56447300
C	2.42018800	-4.44713200	-0.61103900
H	3.34784700	-4.23594200	-1.14410000
C	-1.36647900	-5.23013800	1.36646100
C	-2.05841100	-3.89819600	1.68637900
H	-3.10002700	-4.06437100	1.99909900
H	-2.05765100	-3.20459100	0.83427600
H	-1.55790900	-3.36879100	2.50924000
C	-2.24025600	-6.04673300	0.39962000
H	-3.22047000	-6.25979400	0.85603700
H	-1.76050200	-7.00617400	0.15148700
H	-2.41997800	-5.50958100	-0.54181900
C	-1.22612300	-6.02494800	2.66618200
H	-0.60384300	-5.49835500	3.40696500
H	-0.78915300	-7.02131800	2.49740500
H	-2.21847700	-6.17016700	3.11997700
C	3.72222000	-5.83328900	1.07331200
C	3.99980700	-7.08936200	0.23050500
H	4.93705800	-7.57326700	0.55059100
H	4.09095500	-6.83577800	-0.83652300
H	3.18239400	-7.81961300	0.33318900
C	3.63317100	-6.24318700	2.54682900
H	3.37311700	-5.39289300	3.19686000
H	4.60435900	-6.63632300	2.88385300
H	2.88892700	-7.03565100	2.71431000
C	4.88938100	-4.85422600	0.91209300
H	4.67755600	-3.89394200	1.40556500
H	5.10336900	-4.63787600	-0.14376300
H	5.80887400	-5.26969600	1.35234700
C	2.44150000	-2.27798700	2.97589200
H	3.00946400	-3.20847500	2.92349900
C	1.06003100	-2.31965200	3.19152200
H	0.56294800	-3.28534100	3.29983100
C	0.31709300	-1.13438700	3.24437700
H	-0.76486300	-1.15787300	3.40113300
C	0.96328500	0.09543900	3.07840500
H	0.39088900	1.02321500	3.10063800
C	2.34466500	0.13490400	2.86757800
H	2.84259800	1.09073200	2.70581400
C	3.08932700	-1.04684600	2.81685900
H	4.16547700	-0.99902900	2.63483900
H	-0.88991900	-1.10925900	-0.11714600
Sr	-1.55227200	1.10620700	-0.28741400
N	-2.91081500	1.49111400	1.78754800
C	-3.07041900	1.44702400	-3.00672100
C	-3.44470700	0.42365500	2.46674700
C	-2.53899900	0.15701200	-3.09389900
H	-3.21396800	-0.69749800	-3.04272900

C	-2.09976900	2.12155400	2.69697900
C	-2.21545600	2.55432300	-3.04439400
H	-4.14904500	1.57684900	-2.90101000
C	-4.28097800	-0.59818800	1.97018900
C	-2.98520900	0.35312200	3.81803900
C	-1.15788200	-0.03471300	-3.21084300
C	-2.09635500	1.47476500	3.97181600
C	-1.23871500	3.22159000	2.47740200
H	-2.61578700	3.56596200	-2.96631400
C	-0.83622100	2.36461300	-3.16991400
C	-4.60482400	-1.65039500	2.83137700
C	-4.80539100	-0.61464600	0.58050400
C	-3.34208800	-0.71157900	4.64768600
C	-0.30353400	1.07376300	-3.24979100
H	-0.74143000	-1.04328100	-3.26517100
C	-1.25902600	1.91533400	4.99702700
C	-0.38732000	3.60455800	3.52035700
C	-1.23089400	3.96031600	1.19206600
H	-0.17310300	3.23057500	-3.18543200
H	-5.25392600	-2.42960700	2.42902000
C	-4.15516800	-1.73968100	4.16471200
C	-5.66325800	0.39522900	0.10874700
C	-4.54324800	-1.71247400	-0.24129400
H	-2.96511900	-0.73797700	5.67436000
H	0.77593600	0.92676000	-3.33415500
H	-1.27465300	1.39562600	5.95923800
C	-0.37399300	2.97847400	4.78305600
H	0.27199800	4.45305200	3.33259200
C	-0.02916400	4.25943700	0.53616700
C	-2.43408400	4.41957900	0.63204400
C	-4.52623500	-2.91135100	5.08293800
H	-5.87207000	1.23443200	0.77250000
C	-6.29070400	0.28375600	-1.13367900
C	-5.11213700	-1.83736500	-1.51702300
H	-3.89170400	-2.49323600	0.14993200
C	0.58021500	3.41095400	5.90329700
H	0.89782000	3.87579800	0.96535500
C	-0.00339100	5.02311200	-0.63835400
C	-2.44723500	5.20289200	-0.52621800
H	-3.36052000	4.17843800	1.15589500
C	-5.40989800	-3.94473700	4.38011900
C	-3.24453300	-3.61952700	5.55095400
C	-5.29095400	-2.38065900	6.30536500
C	-5.97484800	-0.82585600	-1.94009700
C	-7.38197700	1.25530800	-1.60483600
C	-4.86177500	-3.11057500	-2.33465700
C	1.47980500	2.22574000	6.28918200
C	-0.23356200	3.85594900	7.12771200
C	1.48412000	4.57097200	5.47945800
C	-1.22178800	5.48584200	-1.14747700
C	1.34482200	5.37355300	-1.27587300
C	-3.77179200	5.79105600	-1.02481600
H	-4.90737300	-4.38610300	3.50537500
H	-5.64884300	-4.76596300	5.07366300
H	-6.36149400	-3.50700800	4.04211400
H	-2.68145800	-4.01579800	4.69207600
H	-2.57937200	-2.93672600	6.10051100
H	-3.48589000	-4.46177600	6.22020400
H	-5.56339000	-3.20560400	6.98425600
H	-4.68943800	-1.65934700	6.87842500
H	-6.21578500	-1.87032200	5.99500500
H	-6.45591900	-0.91291800	-2.91514300
C	-7.68370900	2.33170200	-0.56096200
C	-6.96334200	1.94129200	-2.91280200
C	-8.67558700	0.45838300	-1.84644100
C	-5.36983000	-2.99057500	-3.77306100
C	-5.60688000	-4.26272700	-1.63841400
C	-3.36633600	-3.44889700	-2.38033900
H	2.07664300	1.88992400	5.42696000

H	2.17443900	2.51006500	7.09658800
H	0.89297500	1.36374700	6.63977500
H	-0.87636400	3.04626900	7.50420600
H	0.43436400	4.16367900	7.94904300
H	-0.88396000	4.70762100	6.87525600
H	2.11270600	4.30252700	4.61671800
H	0.90329200	5.46803400	5.21550600
H	2.15659400	4.84238400	6.30812500
H	-1.21690800	6.09264600	-2.05086500
C	2.17312500	4.10049900	-1.49168200
C	2.08934500	6.31489200	-0.31357300
C	1.19255800	6.08614200	-2.62132000
C	-4.22097200	6.87152200	-0.02699700
C	-3.64532500	6.43275300	-2.40882500
C	-4.84080600	4.69445000	-1.09241700
H	-6.79936400	2.94156700	-0.33260000
H	-8.46587800	3.00877100	-0.93777800
H	-8.04760000	1.89197900	0.37986000
H	-6.70653300	1.21254300	-3.69644400
H	-7.78203200	2.57028600	-3.29744700
H	-6.09317000	2.59509900	-2.75438100
H	-8.55767100	-0.28966000	-2.64425200
H	-8.98410400	-0.07152600	-0.93226000
H	-9.49211800	1.13634700	-2.14289500
H	-5.11541200	-3.90169300	-4.33607900
H	-6.46292200	-2.87145300	-3.81849500
H	-4.90956800	-2.13807200	-4.29781700
H	-5.47855500	-5.20285000	-2.19928500
H	-5.22705800	-4.42260600	-0.61766500
H	-6.68392100	-4.04683200	-1.56640000
H	-2.78774900	-2.71971900	-2.96486500
H	-2.92568700	-3.48126900	-1.37667700
H	-3.21564000	-4.43655300	-2.84307400
H	3.19719800	4.35444200	-1.80426300
H	2.23399700	3.48420000	-0.58605000
H	1.74652400	3.45407100	-2.27143900
H	3.07521900	6.58678300	-0.72448300
H	1.51663500	7.24102700	-0.15076300
H	2.24902000	5.84525700	0.66755500
H	0.62966600	5.47952500	-3.34822900
H	0.68454100	7.05758700	-2.52149800
H	2.18623300	6.27719600	-3.05458500
H	-5.17653400	7.31947200	-0.34482200
H	-4.35940000	6.45178100	0.98054700
H	-3.47112900	7.67432800	0.04455000
H	-3.29474400	5.71624900	-3.16855000
H	-4.62700500	6.80749300	-2.73628000
H	-2.95384100	7.28856000	-2.40565700
H	-4.54009500	3.88433900	-1.77326200
H	-5.02542900	4.24095600	-0.10869900
H	-5.79671100	5.10401400	-1.45425200

4.3.1 Compound 2-D

0 1

Sr	1.41779200	-1.10874800	0.22277500
H(iso=2)	0.75237500	1.10251600	0.02427700
N	2.88056300	-1.49198000	-1.77067400
C	3.39162300	-0.40774900	-2.44042200
C	4.21631300	0.62257700	-1.93825200
C	4.51156200	1.69088800	-2.79037200
H	5.15459400	2.47468900	-2.38734000
C	4.05186900	1.78513300	-4.12024300
C	3.25732400	0.74610700	-4.61001000
H	2.87548800	0.77503600	-5.63478700
C	2.92401300	-0.33277400	-3.78888400
C	2.05579000	-1.46969000	-3.95007700
C	1.23678800	-1.92821900	-4.98172200
H	1.24172600	-1.40184900	-5.94031900

C	0.39519500	-3.02930800	-4.78482700
C	0.40929600	-3.65370800	-3.52063000
H	-0.22756600	-4.52075800	-3.33961900
C	1.23559400	-3.24496100	-2.46744800
C	2.07535000	-2.12761100	-2.68166300
C	4.79797500	0.60887600	-0.57109300
C	5.65544000	-0.42839300	-0.17114200
H	5.79479400	-1.26360700	-0.85751100
C	6.36212700	-0.35597000	1.03262000
C	6.13536100	0.75123300	1.86475200
H	6.68575900	0.81774000	2.80514300
C	5.26186200	1.78716000	1.52227400
C	4.60355400	1.69698900	0.28877400
H	3.93163900	2.48692000	-0.04174700
C	7.40841900	-1.39709700	1.44977900
C	7.61703800	-2.46250200	0.37260500
H	6.69201300	-3.01156700	0.15272800
H	8.36662400	-3.19484600	0.71019200
H	7.98120500	-2.01935400	-0.56663400
C	6.96480200	-2.08275000	2.75079300
H	6.81817700	-1.35711700	3.56524800
H	7.72230900	-2.81076100	3.08313400
H	6.01939900	-2.62701000	2.60871900
C	8.75487400	-0.69255500	1.68317400
H	8.70120400	0.04627700	2.49633600
H	9.08407500	-0.16748400	0.77337700
H	9.52889200	-1.42858600	1.95382300
C	5.13390700	3.02385200	2.42180400
C	5.10257800	2.64147900	3.90829400
H	4.98099500	3.54430300	4.52693400
H	6.03104200	2.14982300	4.23275300
H	4.26649100	1.96467700	4.13982400
C	6.35906500	3.91698800	2.16237600
H	6.31524300	4.82813800	2.78126000
H	6.40517700	4.22009600	1.10502300
H	7.29350300	3.38557700	2.39919600
C	3.87075500	3.83023300	2.10540000
H	2.95433000	3.22840000	2.21399500
H	3.88884900	4.23374100	1.08388900
H	3.79029700	4.68754300	2.79111400
C	4.39409900	2.97231600	-5.02981500
C	5.29251000	3.99880200	-4.33586100
H	4.80996900	4.42989200	-3.44536500
H	5.51471400	4.82803300	-5.02546800
H	6.25167900	3.55757100	-4.02470000
C	3.09572800	3.67928300	-5.45117100
H	2.55786500	4.06306000	-4.57085600
H	2.41764000	2.99807700	-5.98691700
H	3.31173200	4.53075800	-6.11752700
C	5.12843000	2.46637600	-6.28098300
H	5.38075500	3.30511100	-6.95058100
H	4.51539100	1.75447300	-6.85339400
H	6.06255800	1.95379900	-6.00399900
C	-0.51760200	-3.50046600	-5.92492300
C	-1.55951700	-2.40954000	-6.22114000
H	-2.19685500	-2.22644800	-5.34193300
H	-2.21303600	-2.70811000	-7.05746200
H	-1.08186400	-1.45478400	-6.48833400
C	0.31904800	-3.76147700	-7.18662700
H	0.83841300	-2.85532500	-7.53135000
H	-0.32384700	-4.10938400	-8.01158800
H	1.08210300	-4.53191400	-6.99649900
C	-1.26104300	-4.79242600	-5.57795000
H	-1.92809200	-4.66110200	-4.71353100
H	-0.56409700	-5.61418300	-5.35249600
H	-1.88443600	-5.10511200	-6.43008900
C	1.22333600	-3.97088400	-1.17239800
C	0.01976500	-4.22512100	-0.50174400
H	-0.89726800	-3.82150500	-0.93217500

C	-0.01729500	-4.96039700	0.69235600
C	1.19263600	-5.44125700	1.20078600
H	1.18164400	-6.02633100	2.11833100
C	2.42119000	-5.20373600	0.56447300
C	2.42018800	-4.44713200	-0.61103900
H	3.34784700	-4.23594200	-1.14410000
C	-1.36647900	-5.23013800	1.36646100
C	-2.05841100	-3.89819600	1.68637900
H	-3.10002700	-4.06437100	1.99909900
H	-2.05765100	-3.20459100	0.83427600
H	-1.55790900	-3.36879100	2.50924000
C	-2.24025600	-6.04673300	0.39962000
H	-3.22047000	-6.25979400	0.85603700
H	-1.76050200	-7.00617400	0.15148700
H	-2.41997800	-5.50958100	-0.54181900
C	-1.22612300	-6.02494800	2.66618200
H	-0.60384300	-5.49835500	3.40696500
H	-0.78915300	-7.02131800	2.49740500
H	-2.21847700	-6.17016700	3.11997700
C	3.72222000	-5.83328900	1.07331200
C	3.99980700	-7.08936200	0.23050500
H	4.93705800	-7.57326700	0.55059100
H	4.09095500	-6.83577800	-0.83652300
H	3.18239400	-7.81961300	0.33318900
C	3.63317100	-6.24318700	2.54682900
H	3.37311700	-5.39289300	3.19686000
H	4.60435900	-6.63632300	2.88385300
H	2.88892700	-7.03565100	2.71431000
C	4.88938100	-4.85422600	0.91209300
H	4.67755600	-3.89394200	1.40556500
H	5.10336900	-4.63787600	-0.14376300
H	5.80887400	-5.26969600	1.35234700
C	2.44150000	-2.27798700	2.97589200
H	3.00946400	-3.20847500	2.92349900
C	1.06003100	-2.31965200	3.19152200
H	0.56294800	-3.28534100	3.29983100
C	0.31709300	-1.13438700	3.24437700
H	-0.76486300	-1.15787300	3.40113300
C	0.96328500	0.09543900	3.07840500
H	0.39088900	1.02321500	3.10063800
C	2.34466500	0.13490400	2.86757800
H	2.84259800	1.09073200	2.70581400
C	3.08932700	-1.04684600	2.81685900
H	4.16547700	-0.99902900	2.63483900
H(iso=2)	-0.88991900	-1.10925900	-0.11714600
Sr	-1.55227200	1.10620700	-0.28741400
N	-2.91081500	1.49111400	1.78754800
C	-3.07041900	1.44702400	-3.00672100
C	-3.44470700	0.42365500	2.46674700
C	-2.53899900	0.15701200	-3.09389900
H	-3.21396800	-0.69749800	-3.04272900
C	-2.09976900	2.12155400	2.69697900
C	-2.21545600	2.55432300	-3.04439400
H	-4.14904500	1.57684900	-2.90101000
C	-4.28097800	-0.59818800	1.97018900
C	-2.98520900	0.35312200	3.81803900
C	-1.15788200	-0.03471300	-3.21084300
C	-2.09635500	1.47476500	3.97181600
C	-1.23871500	3.22159000	2.47740200
H	-2.61578700	3.56596200	-2.96631400
C	-0.83622100	2.36461300	-3.16991400
C	-4.60482400	-1.65039500	2.83137700
C	-4.80539100	-0.61464600	0.58050400
C	-3.34208800	-0.71157900	4.64768600
C	-0.30353400	1.07376300	-3.24979100
H	-0.74143000	-1.04328100	-3.26517100
C	-1.25902600	1.91533400	4.99702700
C	-0.38732000	3.60455800	3.52035700
C	-1.23089400	3.96031600	1.19206600

H	-0.17310300	3.23057500	-3.18543200
H	-5.25392600	-2.42960700	2.42902000
C	-4.15516800	-1.73968100	4.16471200
C	-5.66325800	0.39522900	0.10874700
C	-4.54324800	-1.71247400	-0.24129400
H	-2.96511900	-0.73797700	5.67436000
H	0.77593600	0.92676000	-3.33415500
H	-1.27465300	1.39562600	5.95923800
C	-0.37399300	2.97847400	4.78305600
H	0.27199800	4.45305200	3.33259200
C	-0.02916400	4.25943700	0.53616700
C	-2.43408400	4.41957900	0.63204400
C	-4.52623500	-2.91135100	5.08293800
H	-5.87207000	1.23443200	0.77250000
C	-6.29070400	0.28375600	-1.13367900
C	-5.11213700	-1.83736500	-1.51702300
H	-3.89170400	-2.49323600	0.14993200
C	0.58021500	3.41095400	5.90329700
H	0.89782000	3.87579800	0.96535500
C	-0.00339100	5.02311200	-0.63835400
C	-2.44723500	5.20289200	-0.52621800
H	-3.36052000	4.17843800	1.15589500
C	-5.40989800	-3.94473700	4.38011900
C	-3.24453300	-3.61952700	5.55095400
C	-5.29095400	-2.38065900	6.30536500
C	-5.97484800	-0.82585600	-1.94009700
C	-7.38197700	1.25530800	-1.60483600
C	-4.86177500	-3.11057500	-2.33465700
C	1.47980500	2.22574000	6.28918200
C	-0.23356200	3.85594900	7.12771200
C	1.48412000	4.57097200	5.47945800
C	-1.22178800	5.48584200	-1.14747700
C	1.34482200	5.37355300	-1.27587300
C	-3.77179200	5.79105600	-1.02481600
H	-4.90737300	-4.38610300	3.50537500
H	-5.64884300	-4.76596300	5.07366300
H	-6.36149400	-3.50700800	4.04211400
H	-2.68145800	-4.01579800	4.69207600
H	-2.57937200	-2.93672600	6.10051100
H	-3.48589000	-4.46177600	6.22020400
H	-5.56339000	-3.20560400	6.98425600
H	-4.68943800	-1.65934700	6.87842500
H	-6.21578500	-1.87032200	5.99500500
H	-6.45591900	-0.91291800	-2.91514300
C	-7.68370900	2.33170200	-0.56096200
C	-6.96334200	1.94129200	-2.91280200
C	-8.67558700	0.45838300	-1.84644100
C	-5.36983000	-2.99057500	-3.77306100
C	-5.60688000	-4.26272700	-1.63841400
C	-3.36633600	-3.44889700	-2.38033900
H	2.07664300	1.88992400	5.42696000
H	2.17443900	2.51006500	7.09658800
H	0.89297500	1.36374700	6.63977500
H	-0.87636400	3.04626900	7.50420600
H	0.43436400	4.16367900	7.94904300
H	-0.88396000	4.70762100	6.87525600
H	2.11270600	4.30252700	4.61671800
H	0.90329200	5.46803400	5.21550600
H	2.15659400	4.84238400	6.30812500
H	-1.21690800	6.09264600	-2.05086500
C	2.17312500	4.10049900	-1.49168200
C	2.08934500	6.31489200	-0.31357300
C	1.19255800	6.08614200	-2.62132000
C	-4.22097200	6.87152200	-0.02699700
C	-3.64532500	6.43275300	-2.40882500
C	-4.84080600	4.69445000	-1.09241700
H	-6.79936400	2.94156700	-0.33260000
H	-8.46587800	3.00877100	-0.93777800
H	-8.04760000	1.89197900	0.37986000

H	-6.70653300	1.21254300	-3.69644400
H	-7.78203200	2.57028600	-3.29744700
H	-6.09317000	2.59509900	-2.75438100
H	-8.55767100	-0.28966000	-2.64425200
H	-8.98410400	-0.07152600	-0.93226000
H	-9.49211800	1.13634700	-2.14289500
H	-5.11541200	-3.90169300	-4.33607900
H	-6.46292200	-2.87145300	-3.81849500
H	-4.90956800	-2.13807200	-4.29781700
H	-5.47855500	-5.20285000	-2.19928500
H	-5.22705800	-4.42260600	-0.61766500
H	-6.68392100	-4.04683200	-1.56640000
H	-2.78774900	-2.71971900	-2.96486500
H	-2.92568700	-3.48126900	-1.37667700
H	-3.21564000	-4.43655300	-2.84307400
H	3.19719800	4.35444200	-1.80426300
H	2.23399700	3.48420000	-0.58605000
H	1.74652400	3.45407100	-2.27143900
H	3.07521900	6.58678300	-0.72448300
H	1.51663500	7.24102700	-0.15076300
H	2.24902000	5.84525700	0.66755500
H	0.62966600	5.47952500	-3.34822900
H	0.68454100	7.05758700	-2.52149800
H	2.18623300	6.27719600	-3.05458500
H	-5.17653400	7.31947200	-0.34482200
H	-4.35940000	6.45178100	0.98054700
H	-3.47112900	7.67432800	0.04455000
H	-3.29474400	5.71624900	-3.16855000
H	-4.62700500	6.80749300	-2.73628000
H	-2.95384100	7.28856000	-2.40565700
H	-4.54009500	3.88433900	-1.77326200
H	-5.02542900	4.24095600	-0.10869900
H	-5.79671100	5.10401400	-1.45425200

4.3.1 Compound 4

0 1

Sr	15.20765000	11.33305000	15.76519000
O	16.40934000	11.70938000	17.84131000
O	17.45652000	10.21543000	15.85344000
N	12.96440000	12.42731000	16.12742000
C	16.47184000	10.37280000	17.95590000
C	16.99383000	9.63617000	16.97007000
C	14.47999000	13.58252000	13.80231000
C	15.82763000	13.86577000	14.04590000
C	12.94242000	9.35473000	16.84332000
C	14.55210000	8.34194000	14.49142000
C	14.15677000	12.59163000	12.87906000
C	16.85350000	13.16536000	13.39866000
Sr	18.82829000	11.59607000	17.40051000
N	21.01192000	12.73851000	16.98133000
C	21.15958000	9.54595000	16.95206000
C	19.39055000	14.39004000	18.93948000
C	19.56346000	13.47023000	19.98902000
C	12.70628000	13.66790000	15.57766000
C	12.02177000	12.30703000	17.14342000
C	21.26863000	14.06073000	17.29020000
C	21.98277000	12.41784000	16.04322000
C	11.85129000	11.25163000	18.06847000
C	11.17781000	13.43930000	17.23144000
C	10.81880000	11.38866000	19.00769000
C	12.68473000	10.02009000	18.04280000
C	9.96425000	12.48322000	19.08919000
C	10.14741000	13.52712000	18.18306000
C	8.83031000	12.49073000	20.11915000
C	11.63845000	14.34365000	16.21815000
C	11.27245000	15.62681000	15.83160000

C	11.96276000	16.28337000	14.81921000
C	13.02082000	15.59731000	14.18480000
C	11.51399000	17.68565000	14.37503000
C	13.39186000	14.30377000	14.52902000
C	13.15429000	9.46467000	19.23507000
C	13.62569000	8.13766000	16.81631000
C	14.06019000	7.60690000	18.03537000
C	13.84055000	7.40672000	15.48356000
C	13.84091000	8.25172000	19.25398000
C	14.41731000	7.58835000	20.52032000
C	14.71986000	6.16805000	15.63777000
C	12.48673000	6.99206000	14.91061000
C	13.74255000	6.23514000	20.73645000
C	14.19933000	8.45040000	21.76303000
C	15.93320000	7.35056000	20.37174000
C	7.91027000	11.30789000	19.85395000
C	7.99080000	13.77370000	20.06219000
C	9.42275000	12.39243000	21.53902000
C	10.19435000	17.55338000	13.61455000
C	12.54482000	18.36649000	13.47205000
C	11.31001000	18.58829000	15.60378000
C	16.47807000	12.19741000	12.45718000
C	18.32600000	13.47594000	13.63256000
C	15.14569000	11.91127000	12.15528000
C	14.79055000	11.04426000	10.90290000
C	18.84823000	14.32333000	12.46596000
C	19.15922000	12.18701000	13.69605000
C	18.53528000	14.25650000	14.94168000
C	15.97706000	10.23582000	10.38286000
C	13.63270000	10.09305000	11.21808000
C	14.32657000	11.99112000	9.78852000
C	22.15494000	11.19858000	15.35276000
C	22.83620000	13.50698000	15.73728000
C	23.17297000	11.14779000	14.39777000
C	21.32044000	10.00362000	15.63687000
C	24.02139000	12.21172000	14.08574000
C	23.85147000	13.39514000	14.78386000
C	25.15364000	11.98600000	13.07583000
C	22.38341000	14.58675000	16.56957000
C	22.80494000	15.90046000	16.76385000
C	22.12900000	16.72647000	17.66054000
C	21.00390000	16.20393000	18.33434000
C	22.66978000	18.13538000	17.95365000
C	20.56783000	14.89255000	18.17585000
C	20.74648000	9.26721000	14.60241000
C	20.48511000	8.36219000	17.23594000
C	19.91371000	7.66072000	16.17402000
C	20.46022000	7.83336000	18.67946000
C	20.01226000	8.10403000	14.85523000
C	19.34941000	7.36240000	13.68975000
C	19.60931000	6.58208000	18.83367000
C	21.88726000	7.51934000	19.11891000
C	19.88630000	8.90827000	19.60023000
C	18.49526000	8.33184000	12.87884000
C	18.44049000	6.23093000	14.17107000
C	20.43488000	6.75216000	12.79779000
C	26.14614000	11.00021000	13.69965000
C	25.89623000	13.28393000	12.74489000
C	24.61296000	11.41869000	11.75861000
C	21.70025000	18.96811000	18.79585000
C	24.00613000	17.99728000	18.69837000
C	22.90842000	18.90738000	16.64476000
C	18.11214000	14.85676000	18.65740000
C	16.99963000	14.44593000	19.39672000
C	17.22727000	13.57582000	20.45729000
C	15.60008000	14.98316000	19.03674000
C	18.49304000	13.08472000	20.78170000
C	18.66037000	12.18977000	22.02034000
C	15.35951000	14.85025000	17.53424000

C	15.53057000	16.46490000	19.40009000
C	14.49143000	14.21384000	19.75714000
C	17.71382000	10.99154000	21.90936000
C	20.09979000	11.69337000	22.19302000
C	18.29358000	13.00409000	23.27790000
H	10.69335000	10.68382000	19.63238000
H	9.58142000	14.28971000	18.20845000
H	10.54446000	16.05907000	16.26254000
H	13.49945000	16.03899000	13.49323000
H	12.64607000	9.73885000	16.02665000
H	14.52167000	6.77647000	18.03407000
H	13.00225000	9.92433000	20.05244000
H	14.70306000	7.86830000	13.64677000
H	15.41349000	8.62170000	14.86691000
H	13.99507000	9.13114000	14.32872000
H	14.89834000	5.78276000	14.75469000
H	14.25780000	5.50734000	16.19514000
H	15.56609000	6.41938000	16.06231000
H	12.62258000	6.51922000	14.06280000
H	11.93927000	7.78932000	14.75633000
H	12.03140000	6.39995000	15.54515000
H	16.27407000	6.90083000	21.17267000
H	16.38967000	8.21172000	20.26201000
H	16.10149000	6.79031000	19.58553000
H	14.12122000	5.80579000	21.53153000
H	13.89551000	5.66601000	19.95336000
H	12.78035000	6.36713000	20.86218000
H	14.59896000	8.00765000	22.54028000
H	13.23872000	8.56985000	21.91199000
H	14.62178000	9.32432000	21.63092000
H	8.69620000	12.38407000	22.19635000
H	10.00442000	13.16366000	21.70456000
H	9.94485000	11.56675000	21.61852000
H	7.20065000	11.28721000	20.53009000
H	8.42609000	10.47572000	19.89695000
H	7.50968000	11.39795000	18.96377000
H	7.29445000	13.73933000	20.75146000
H	7.57185000	13.85111000	19.17931000
H	8.56767000	14.54971000	20.21922000
H	12.23399000	19.26750000	13.24395000
H	12.65675000	17.84215000	12.65171000
H	13.40217000	18.42550000	13.94211000
H	11.02372000	19.47886000	15.31107000
H	12.15364000	18.66306000	16.09773000
H	10.62448000	18.19928000	16.18535000
H	9.87270000	18.44293000	13.36084000
H	9.53005000	17.11590000	14.18705000
H	10.33536000	17.01421000	12.80786000
H	16.05283000	14.54986000	14.66538000
H	17.16004000	11.71511000	12.00476000
H	13.24381000	12.37138000	12.73613000
H	19.79874000	14.51827000	12.60376000
H	18.34543000	15.16303000	12.42138000
H	18.73453000	13.82934000	11.62706000
H	20.07537000	12.40398000	13.96904000
H	19.17349000	11.76368000	12.81245000
H	18.76122000	11.57256000	14.34691000
H	19.49402000	14.31084000	15.13981000
H	18.07622000	13.79574000	15.67422000
H	18.17071000	15.16103000	14.84469000
H	13.42370000	9.55767000	10.42408000
H	12.84386000	10.61247000	11.47871000
H	13.88955000	9.49813000	11.95395000
H	14.19509000	11.48310000	8.96134000
H	15.00713000	12.68221000	9.64237000
H	13.48281000	12.41505000	10.05001000
H	15.68486000	9.66835000	9.63877000
H	16.32981000	9.67269000	11.10338000
H	16.67828000	10.84579000	10.07214000

H	16.14431000	9.94963000	18.74058000
H	17.03595000	8.69162000	17.06174000
H	23.29666000	10.33165000	13.92735000
H	24.42622000	14.13238000	14.61439000
H	23.55426000	16.23376000	16.28456000
H	20.52276000	16.77382000	18.92262000
H	21.52001000	10.05658000	17.66740000
H	19.44275000	6.85573000	16.35476000
H	20.85627000	9.56158000	13.70591000
H	19.89865000	8.58577000	20.52566000
H	18.96333000	9.10519000	19.33550000
H	20.42725000	9.72177000	19.53008000
H	19.63822000	6.27972000	19.76570000
H	19.95860000	5.87510000	18.25089000
H	18.68366000	6.78319000	18.58401000
H	21.88140000	7.20378000	20.04657000
H	22.43496000	8.32993000	19.05337000
H	22.26243000	6.82427000	18.53932000
H	18.04584000	5.77756000	13.39699000
H	17.72606000	6.60205000	14.73080000
H	18.96497000	5.58973000	14.69352000
H	20.01379000	6.26380000	12.05947000
H	20.98334000	6.13545000	13.32617000
H	21.00083000	7.46568000	12.43664000
H	18.03600000	7.84396000	12.16460000
H	19.06999000	9.02255000	12.48527000
H	17.83380000	8.75389000	13.46545000
H	26.88314000	10.83886000	13.07407000
H	25.68984000	10.15493000	13.89220000
H	26.50115000	11.37702000	14.53199000
H	25.35820000	11.26213000	11.14099000
H	23.98808000	12.05964000	11.35964000
H	24.14940000	10.57409000	11.93252000
H	26.57608000	13.10660000	12.06129000
H	26.32967000	13.62815000	13.55404000
H	25.25982000	13.94831000	12.40774000
H	24.36140000	18.88813000	18.90031000
H	24.64491000	17.51096000	18.13607000
H	23.86546000	17.50470000	19.53375000
H	23.25246000	19.80143000	16.85080000
H	22.06338000	18.98763000	16.15446000
H	23.56030000	18.42481000	16.09498000
H	22.08136000	19.85871000	18.94669000
H	21.55377000	18.52565000	19.65779000
H	20.84703000	19.05371000	18.32259000
H	17.99142000	15.47126000	17.94300000
H	16.48650000	13.30316000	20.98602000
H	20.42542000	13.10800000	20.15749000
H	14.66924000	16.83238000	19.11326000
H	16.25712000	16.94597000	18.94957000
H	15.62411000	16.56864000	20.37014000
H	13.61908000	14.51011000	19.42456000
H	14.54893000	14.38584000	20.72109000
H	14.59733000	13.25449000	19.59028000
H	14.46803000	15.19264000	17.31476000
H	15.41952000	13.90647000	17.27595000
H	16.03654000	15.36542000	17.04830000
H	20.16337000	11.14907000	23.00534000
H	20.70395000	12.46224000	22.26751000
H	20.35504000	11.15261000	21.41653000
H	18.39633000	12.44173000	24.07366000
H	17.36393000	13.30791000	23.21057000
H	18.88634000	13.78152000	23.34846000
H	17.81760000	10.41962000	22.69834000
H	17.93031000	10.47840000	21.10307000
H	16.78918000	11.31043000	21.85580000

4.3.2 Compound 5 - monomer

0 2

Sr	-0.96351700	-0.25007700	-0.24358700
N	1.46152400	-0.33150800	-0.74815200
N	-1.46488000	0.66190100	2.05499700
C	-3.79382000	0.57806300	0.46745700
C	-0.38498600	-2.97273300	-1.54936700
C	2.26745200	-1.43485100	-0.60008000
C	-3.67906800	0.16610100	1.82800800
N	-2.62295400	0.41997500	2.64505800
C	0.40594900	-3.05182800	-0.38628200
C	-0.79171400	1.78328200	4.13801400
C	-0.49439800	1.21147000	2.88327100
C	2.33279800	0.73193400	-0.76774700
C	1.85922800	-2.77095600	-0.43606100
C	3.64804000	-1.10556400	-0.52715900
C	2.83328700	-3.74089200	-0.23325100
C	4.21540500	-3.44049100	-0.19639500
H	2.50353600	-4.77443900	-0.09726200
C	4.60588400	-2.10909800	-0.33746600
C	5.21577000	-4.58109000	0.01226200
H	5.66008800	-1.83269300	-0.28752200
C	3.68987500	0.33159700	-0.61865600
C	4.70657800	1.28831800	-0.51063400
C	4.39718300	2.64768700	-0.52793700
H	5.73801300	0.95431200	-0.38859800
C	3.04265800	3.02278000	-0.67876100
C	5.45567600	3.74333100	-0.37523400
C	2.00863300	2.10217900	-0.81562900
H	2.78001800	4.08370900	-0.70398400
C	0.60202000	2.53300200	-0.96882100
C	-0.21272900	-3.32638300	0.83578600
C	-1.77139300	-3.15563000	-1.49535100
H	0.12382700	-2.78652900	-2.49563000
C	-2.36046200	-3.39554000	-0.23579200
C	-2.64955900	-3.15666300	-2.75005700
C	-1.60346000	-3.51958600	0.93478600
H	-3.44150100	-3.52276600	-0.17929400
C	-2.21100700	-3.92254900	2.27915200
H	0.41822900	-3.38904100	1.72491700
C	-1.83312100	-2.92575100	-4.02176800
C	-3.70589700	-2.05244400	-2.63549200
C	-3.34573400	-4.52142100	-2.86300100
H	-2.50125300	-2.92408600	-4.89588400
H	-1.08387700	-3.71620000	-4.17648600
H	-1.30792500	-1.95842600	-4.00862500
H	-4.36776900	-2.05469600	-3.51559500
H	-3.23328100	-1.05771700	-2.59075200
H	-4.33318400	-2.15880500	-1.73913700
H	-3.96763900	-4.55713600	-3.77109700
H	-3.99954600	-4.72022200	-2.00142900
H	-2.60659700	-5.33467800	-2.92145100
C	-1.82456800	-2.90984600	3.36637400
C	-3.73422800	-4.02279200	2.21017000
C	-1.64680000	-5.30581400	2.64558800
H	-2.23755900	-3.23038900	4.33537200
H	-2.21068000	-1.90179500	3.15294600
H	-0.73418600	-2.83057600	3.48826700
H	-4.12983700	-4.29105200	3.20118500
H	-4.06231200	-4.80229900	1.50493200
H	-4.19364500	-3.06735400	1.91890600
H	-2.07314600	-5.64386600	3.60298400
H	-0.55223800	-5.28471300	2.75469600
H	-1.89643300	-6.05293500	1.87623700
C	6.65973700	-4.07891300	0.03703100
C	5.07996800	-5.59545700	-1.13279300
C	4.92794300	-5.27833200	1.35015100
H	5.63884400	-6.10352000	1.51914800

H	3.91288600	-5.70185100	1.38012300
H	5.01913800	-4.56896000	2.18700400
H	5.78948700	-6.42860700	-1.00065600
H	5.28690800	-5.11808700	-2.10295400
H	4.06714100	-6.02270300	-1.18032500
H	7.34744600	-4.92594600	0.18429500
H	6.82927000	-3.36612100	0.85833800
H	6.93551000	-3.58594900	-0.90759400
C	5.44189900	4.65026500	-1.61426000
C	5.14174800	4.57870300	0.87506000
C	6.86207300	3.16343400	-0.22268600
H	7.59352100	3.97942800	-0.11677900
H	7.15350500	2.56626500	-1.10016300
H	6.94480000	2.52469000	0.66982400
H	6.19528000	5.44919200	-1.51920300
H	4.46235200	5.13003800	-1.75783700
H	5.66703800	4.07170300	-2.52339000
H	5.89064900	5.37629800	1.00889000
H	5.14684700	3.94724600	1.77702200
H	4.15251400	5.05562600	0.80920200
C	-0.00453600	3.34857400	-0.00670100
C	-0.13904500	2.14111800	-2.09840800
C	-1.32815800	3.78139200	-0.14488000
H	0.58106600	3.62223900	0.86956700
C	-2.02833200	3.38868100	-1.29399400
C	-2.00163700	4.67655800	0.89840500
C	-1.45283800	2.59188000	-2.29696000
H	-3.05174100	3.74505300	-1.42698900
C	-2.18631700	2.37006300	-3.62413300
H	0.37021300	1.53980000	-2.85327500
C	-1.04968600	5.03109900	2.04216300
C	-3.21832200	3.95114400	1.48920000
C	-2.45397400	5.97724400	0.21821400
H	-1.57100000	5.67593900	2.76559300
H	-0.16663000	5.58221300	1.68404800
H	-0.70688100	4.13781900	2.58496100
H	-2.92545300	6.64546500	0.95578800
H	-3.18947200	5.79278800	-0.57928300
H	-1.59856600	6.50867500	-0.22686600
H	-3.72103900	4.59798300	2.22514800
H	-2.91833100	3.02537900	2.00120300
H	-3.95844800	3.68853600	0.71827500
C	-1.52524400	1.28086700	-4.47113000
C	-3.64870500	1.97632200	-3.39808900
C	-2.13464000	3.69545500	-4.40209200
H	-2.62921400	3.58865500	-5.38081600
H	-1.09371800	4.00839900	-4.57362300
H	-2.64026600	4.50097500	-3.84889600
H	-4.16677400	1.86962300	-4.36339600
H	-4.20120600	2.72190600	-2.80912300
H	-3.72068200	1.01201400	-2.87475000
H	-2.11116900	1.11198300	-5.38698900
H	-1.47063700	0.32019800	-3.93428800
H	-0.50579000	1.55299000	-4.77941000
C	0.84184400	1.24639200	2.43430300
C	1.83473900	1.86186600	3.18645000
H	1.11913700	0.78404300	1.48273500
C	1.52646000	2.44549600	4.41804200
H	2.85697200	1.88073800	2.79970500
C	0.21066900	2.38975800	4.88648100
H	2.30434900	2.93336800	5.00949600
H	-0.04020000	2.83733100	5.85218900
H	-1.82347100	1.74481500	4.48818000
C	-4.78557800	-0.52896800	2.38595400
C	-4.93424700	0.26823000	-0.27680000
H	-3.08419200	1.31569900	0.07286900
C	-5.97602000	-0.47229400	0.27431700
H	-5.02335800	0.64227400	-1.29893600
C	-5.89325300	-0.85135400	1.62425400

H	-6.86031500	-0.71339400	-0.31882000
H	-6.71833100	-1.40195900	2.08417300
H	-4.72151300	-0.80781600	3.43971500

4.3.3 Compound 5 - dimer

0 3

Sr	-2.00200000	-0.37149400	-0.99074400
N	-3.90042800	-1.46160300	0.22111100
N	-0.04144100	-1.58851500	0.22547000
N	-1.12108500	1.62021600	0.42853700
C	-0.11088100	-2.25671600	-2.46673200
C	-1.23562200	2.32982600	-2.19417700
C	-4.82125600	0.46537200	-1.99038800
N	0.04224500	1.58641600	-0.22541400
C	-0.08001900	1.77110700	-1.59199400
C	-4.62942100	-0.52133500	0.91878400
C	1.05112600	-1.81835600	-1.78578300
N	1.12189100	-1.62222900	-0.42848600
C	-4.91384200	1.29979800	-0.86246700
Sr	2.00300400	0.36977200	0.99034800
N	3.90001300	1.46256500	-0.22118000
C	0.11167300	2.25459700	2.46686500
C	1.23677700	-2.33117900	2.19425400
C	0.08095700	-1.77289100	1.59207500
C	4.62923500	0.52271700	-0.91917900
C	4.82243800	-0.46449100	1.98992200
C	-1.05032100	1.81630400	1.78584600
C	4.91485600	-1.29859600	0.86173300
C	-3.67488200	-2.48816800	1.11694400
C	3.67387700	2.48919000	-1.11678800
C	-5.10431200	0.75275800	0.49851300
C	-4.84401300	-0.91735600	2.27450900
C	-5.76038700	1.53455200	1.45508500
C	-5.95463500	1.16275700	2.79854000
H	-6.16125200	2.49446100	1.12571000
C	-5.48573500	-0.08732700	3.19658500
C	-6.66851800	2.13184300	3.74396100
H	-5.60666400	-0.43217900	4.22485200
C	-4.21461500	-2.19974700	2.40257800
C	-4.00476000	-3.03948400	3.50180100
C	-3.24615000	-4.19523800	3.35304000
H	-4.42711400	-2.76366600	4.46960000
C	-2.74778500	-4.48793900	2.06719100
C	-2.96520000	-5.16768800	4.50056900
C	-2.93816500	-3.68525700	0.94181800
H	-2.20152500	-5.42182600	1.91820500
C	-2.41779100	-4.18487000	-0.35085200
C	-4.83720200	2.68785100	-1.06800400
C	-4.68214000	0.99200500	-3.28189800
H	-4.90587000	-0.61360600	-1.83309000
C	-4.69054900	2.37982500	-3.44000600
C	-4.52289400	0.03856400	-4.46659000
C	-4.76353800	3.24813300	-2.34425200
H	-4.62004600	2.79688300	-4.44160500
C	-4.75441600	4.77165800	-2.50525800
H	-4.83953900	3.34185000	-0.19484900
C	-5.79202600	-0.81129600	-4.60897800
C	-3.30549400	-0.86058600	-4.20547000
C	-4.28040000	0.77734300	-5.78342300
H	-5.67912800	-1.54924500	-5.41884800
H	-6.65779000	-0.17343700	-4.84263400
H	-6.02345100	-1.35721400	-3.68340100
H	-3.11877600	-1.52061100	-5.06570500
H	-3.45606000	-1.52187900	-3.33504500
H	-2.39349900	-0.25493800	-4.06611900
H	-4.16033100	0.04884200	-6.59948500
H	-3.36579100	1.38836000	-5.74597700

H	-5.12481600	1.43115700	-6.04764800
C	-3.51553700	5.35448200	-1.81220000
C	-4.73084500	5.19728300	-3.97389600
C	-6.02168600	5.34768100	-1.85601200
H	-3.51998800	6.45397600	-1.87880700
H	-2.58819400	4.99232400	-2.28137000
H	-3.47756900	5.08564600	-0.74765600
H	-4.74368900	6.29554000	-4.04066400
H	-5.60548700	4.81986000	-4.52534800
H	-3.82151000	4.85018100	-4.48831500
H	-6.04671100	6.44286400	-1.97119800
H	-6.06634800	5.12440500	-0.77990800
H	-6.92762300	4.93202300	-2.32321200
C	-6.69964400	1.60514000	5.17898100
C	-8.11483300	2.33295400	3.26907900
C	-5.93851900	3.48372400	3.74533800
H	-6.43177900	4.18901800	4.43357700
H	-5.93102800	3.94592100	2.74696100
H	-4.89087200	3.36809900	4.06283900
H	-8.64854200	3.03495200	3.93039800
H	-8.66133800	1.37748700	3.26759900
H	-8.15198500	2.73933500	2.24717300
H	-7.20512300	2.33110100	5.83414500
H	-5.68486900	1.44773200	5.57665800
H	-7.24706200	0.65342400	5.25326700
C	-3.71566000	-6.48224700	4.24170800
C	-1.45810900	-5.44912700	4.58882800
C	-3.42186700	-4.60184500	5.84567600
H	-3.17853900	-5.30950700	6.65325000
H	-4.50900100	-4.43296500	5.87191900
H	-2.92087200	-3.64755500	6.07147600
H	-3.52340900	-7.20827800	5.04885200
H	-3.40307300	-6.94165300	3.29189000
H	-4.80110000	-6.30748200	4.18606900
H	-1.24272300	-6.13039500	5.42795600
H	-0.89359800	-4.51713700	4.74097300
H	-1.07397100	-5.92162500	3.67277100
C	-1.15416300	-4.78286100	-0.41280300
C	-3.24709300	-4.23117600	-1.48592500
C	-0.71939200	-5.47149300	-1.55246100
H	-0.50951000	-4.70964000	0.46525400
C	-1.58915300	-5.53203200	-2.64189100
C	0.65658400	-6.14553400	-1.56523600
C	-2.85752700	-4.93028800	-2.63058300
H	-1.28598700	-6.08608600	-3.52989100
C	-3.80321300	-5.17415400	-3.81158900
H	-4.23607700	-3.77834500	-1.41087000
C	0.81566200	-7.00795800	-0.30481100
C	1.75068500	-5.07086500	-1.59399600
C	0.84046400	-7.04769100	-2.78674300
H	1.79092000	-7.52002300	-0.31545200
H	0.02612200	-7.77313000	-0.24709200
H	0.77131300	-6.40662000	0.61456000
H	1.82397000	-7.53953100	-2.73853200
H	0.80784500	-6.47487200	-3.72580700
H	0.07275600	-7.83553200	-2.83378200
H	2.74766500	-5.53973400	-1.55785800
H	1.66581200	-4.37899400	-0.74360900
H	1.69118700	-4.47139200	-2.51343700
C	-5.13128600	-4.43808500	-3.64433800
C	-3.15317900	-4.72075500	-5.12486200
C	-4.09734200	-6.68087200	-3.88953100
H	-4.79801100	-6.89382400	-4.71296000
H	-4.54916800	-7.03843500	-2.95187100
H	-3.18220300	-7.26545400	-4.06482200
H	-3.81230400	-4.94668500	-5.97800900
H	-2.19131800	-5.22280800	-5.30429800
H	-2.97135500	-3.63641300	-5.11955300
H	-5.77134800	-4.61341400	-4.52262000

H	-4.98685400	-3.35327400	-3.55134000
H	-5.68165100	-4.78484900	-2.75735800
C	-2.22417500	1.60916800	2.54521400
C	0.10422200	2.40807100	3.85249100
H	0.95971600	2.64193500	1.89301400
C	-1.04477600	2.14120000	4.59396800
H	1.00086100	2.78582800	4.34672600
C	-2.21320600	1.75889100	3.92167600
H	-1.04304500	2.26222300	5.67907600
H	-3.13518600	1.57182100	4.47740800
H	-3.14327100	1.32507800	2.03072400
C	0.97976100	1.37487800	-2.43772200
C	0.88303000	1.49819500	-3.81255200
H	1.88840200	0.96012800	-2.00568900
C	-0.26815100	2.03358100	-4.40142200
H	1.72192200	1.17128100	-4.42879500
C	-1.31214100	2.45595600	-3.58176100
H	-0.34018200	2.13377800	-5.48644800
H	-2.20543800	2.90476900	-4.01806800
H	-2.04207900	2.70153400	-1.55859900
C	-0.97878500	-1.37667200	2.43784300
C	-0.88174600	-1.49941900	3.81270500
H	-1.88763900	-0.96241100	2.00579500
C	0.26972900	-2.03418100	4.40157800
H	-1.72062300	-1.17252000	4.42897600
C	1.31365900	-2.45664300	3.58187900
H	0.34203000	-2.13384900	5.48663400
H	2.20717100	-2.90504700	4.01818500
H	2.04314300	-2.70308100	1.55868900
C	2.22490600	-1.61103000	-2.54522900
C	-0.10355600	-2.41005800	-3.85237400
H	-0.95884100	-2.64410100	-1.89279700
C	1.04535300	-2.14299900	-4.59392700
H	-1.00021400	-2.78784800	-4.34656100
C	2.21381400	-1.76065200	-3.92170500
H	1.04353400	-2.26390500	-5.67904700
H	3.13571100	-1.57341800	-4.47752200
H	3.14402200	-1.32680800	-2.03081300
C	4.21355100	2.20127900	-2.40255600
C	2.93656200	3.68585700	-0.94132100
C	2.74561600	4.48867700	-2.06648900
C	2.41613800	4.18484400	0.35156200
C	3.24398100	4.19649900	-3.35246400
H	2.19891100	5.42225900	-1.91723800
C	4.00314900	3.04116000	-3.50156700
C	2.96255300	5.16912100	-4.49973400
H	4.42548400	2.76574600	-4.46949000
C	4.84341700	0.91907600	-2.27488100
C	5.48538500	0.08950100	-3.19718800
C	5.95501800	-1.16040300	-2.79940800
H	5.60601100	0.43459100	-4.22541000
C	5.76115700	-1.53252700	-1.45599200
C	6.66949500	-2.12883900	-3.74504900
C	5.10482100	-0.75121400	-0.49919300
H	6.16259500	-2.49226300	-1.12681200
C	3.24558600	4.23133900	1.48650200
C	1.15214100	4.78203700	0.41387900
C	2.85575000	4.92985200	2.63143900
H	4.23483100	3.77912700	1.41118000
C	1.58697200	5.53071200	2.64316100
C	3.80153700	5.17410700	3.81228100
C	0.71708700	5.46998300	1.55382800
H	1.28359600	6.08431500	3.53136900
C	-0.65929600	6.14318700	1.56704800
H	0.50742400	4.70867300	-0.46411900
C	3.15208300	4.72026200	5.12567000
C	4.09482100	6.68100200	3.89024400
C	5.12999900	4.43880600	3.64468600
H	5.77002100	4.61416700	4.52299200

H	5.68014300	4.78616200	2.75780100
H	4.98612200	3.35395100	3.55131700
H	3.81136300	4.94639700	5.97864300
H	2.97076200	3.63582700	5.12031600
H	2.19004200	5.22183300	5.30546400
H	4.79548800	6.89433400	4.71357600
H	3.17935500	7.26502400	4.06569400
H	4.54628300	7.03885500	2.95252400
C	-0.84386000	7.04423300	2.78927100
C	-0.81872400	7.00655900	0.30732100
C	-1.75275400	5.06783400	1.59481900
H	-1.82761200	7.53560400	2.74128300
H	-0.07657600	7.83243800	2.83710400
H	-0.81111600	6.47064700	3.72786400
H	-1.79445200	7.51773600	0.31804300
H	-0.77348200	6.40606600	-0.61255000
H	-0.02985100	7.77248800	0.25056000
H	-2.75003400	5.53611300	1.55904700
H	-1.69295400	4.46761200	2.51375000
H	-1.66742800	4.37673200	0.74385700
C	3.41839300	4.60333800	-5.84514700
C	3.71336900	6.48352000	-4.24108200
C	1.45547000	5.45085900	-4.58718800
H	1.23976300	6.13196900	-5.42636100
H	1.07194500	5.92371100	-3.67105500
H	0.89068000	4.51895200	-4.73878900
H	3.52062500	7.20979800	-5.04788900
H	4.79882700	6.30860300	-4.18627500
H	3.40152100	6.94267300	-3.29090000
H	3.17461800	5.31105500	-6.65253800
H	2.91722000	3.64907400	-6.07067500
H	4.50550200	4.43441900	-5.87206800
C	8.11612300	-2.32860900	-3.27054200
C	6.69974900	-1.60208100	-5.18007100
C	5.94078400	-3.48140500	-3.74627500
H	7.20560900	-2.32762200	-5.83540500
H	7.24638800	-0.64993200	-5.25452400
H	5.68472000	-1.44547100	-5.57741800
H	8.65032000	-3.03010900	-3.93199900
H	8.15389500	-2.73494900	-2.24864100
H	8.66173700	-1.37263400	-3.26919300
H	6.43449300	-4.18617200	-4.43473100
H	4.89292300	-3.36676800	-4.06342900
H	5.93407700	-3.94370300	-2.74794100
C	4.83856900	-2.68672900	1.06691500
C	4.76544600	-3.24739200	2.34303300
H	4.84079500	-3.34046300	0.19356000
C	4.69255700	-2.37939700	3.43903800
C	4.75686000	-4.77097000	2.50363100
C	4.68382700	-0.99153300	3.28131900
H	4.62241200	-2.79673400	4.44054700
C	4.52469200	-0.03844400	4.46630800
H	4.90673100	0.61455700	1.83292200
C	3.51796700	-5.35405100	1.81080400
C	6.02412400	-5.34637900	1.85383200
C	4.73389700	-5.19698300	3.97216300
H	3.52293300	-6.45357500	1.87694000
H	3.47946100	-5.08476900	0.74639000
H	2.59065600	-4.99252100	2.28050600
H	6.04955100	-6.44158500	1.96870200
H	6.93006900	-4.93055000	2.32086100
H	6.06837600	-5.12279600	0.77777000
H	4.74706000	-6.29525200	4.03865300
H	3.82464100	-4.85025400	4.48697300
H	5.60862100	-4.81945700	4.52341500
C	4.28317500	-0.77765100	5.78308100
C	5.79349900	0.81198200	4.60830200
C	3.30673600	0.86015900	4.20596400
H	5.68068500	1.54956200	5.41851900

H	6.02413800	1.35839000	3.68281600
H	6.65970200	0.17445400	4.84123900
H	4.16312200	-0.04939800	6.59936600
H	5.12803300	-1.43111400	6.04677100
H	3.36884900	-1.38910800	5.74591500
H	3.12022500	1.52003700	5.06635900
H	2.39494500	0.25412100	4.06701000
H	3.45643600	1.52154900	3.33548000

4.3.4 Compound 6

0 1

Sr	0.37226000	3.87689000	6.18784000
N	2.25716000	4.73479000	4.76242000
C	-0.91345000	4.01315000	8.50925000
C	-2.09849000	3.25310000	5.27091000
C	2.14055000	1.75529000	5.47072000
C	0.99899000	0.98768000	5.61404000
C	-1.75872000	3.30480000	4.11751000
C	2.89996000	2.06417000	6.59932000
C	2.57623000	3.68576000	3.90943000
C	2.52936000	2.30563000	4.15058000
C	0.59007000	0.52992000	6.85839000
Sr	-3.38421000	3.38936000	7.59232000
Si	-1.59007000	3.83354000	11.46675000
C	-2.49747000	5.31487000	12.03146000
C	0.00440000	3.72560000	12.37872000
C	-1.25322000	3.96146000	9.66265000
C	-2.56563000	2.28591000	11.74483000
C	2.55375000	5.87513000	4.02105000
H	-2.67441000	2.13634000	12.70715000
H	-2.09104000	1.52488000	11.34811000
H	-3.44673000	2.37496000	11.32604000
H	-0.16805000	3.74902000	13.34249000
H	0.57418000	4.48437000	12.13025000
H	0.45731000	2.88834000	12.14693000
H	-2.58513000	5.29257000	13.00775000
H	-3.38921000	5.32733000	11.62458000
H	-2.00662000	6.12056000	11.76640000
C	2.99725000	5.55798000	2.72434000
C	2.48785000	7.23453000	4.39036000
C	2.87609000	8.16640000	3.43815000
C	2.15790000	7.78216000	5.72841000
C	3.28063000	7.85989000	2.14557000
H	2.86347000	9.08306000	3.68962000
C	3.33725000	6.53241000	1.78866000
C	3.76206000	9.00408000	1.24573000
H	3.60459000	6.28302000	0.91259000
C	3.01249000	4.12920000	2.65130000
C	3.35352000	3.24212000	1.65086000
C	3.27182000	1.87087000	1.87410000
H	3.64460000	3.56786000	0.80684000
C	2.85479000	1.44008000	3.12947000
C	3.65308000	0.89596000	0.75791000
H	2.79168000	0.50603000	3.28870000
C	2.56989000	7.16655000	6.88732000
C	1.58327000	9.04340000	5.81798000
C	2.48901000	7.81752000	8.11651000
H	2.91629000	6.28258000	6.84951000
C	1.94641000	9.08490000	8.14821000
C	3.02551000	7.19651000	9.42563000
C	1.49083000	9.72979000	7.01272000
H	1.88201000	9.53292000	8.98362000
C	1.02594000	11.17715000	7.10229000
H	1.24278000	9.44766000	5.02936000
C	1.87372000	6.55382000	10.16149000
C	4.10900000	6.15434000	9.14451000
C	3.64465000	8.27662000	10.32685000

H	4.46201000	5.81343000	9.99270000
H	4.83434000	6.56805000	8.63141000
H	3.72522000	5.41442000	8.62930000
H	2.20450000	6.12887000	10.97944000
H	1.45603000	5.87755000	9.58748000
H	1.21169000	7.23861000	10.39308000
H	4.07124000	7.85097000	11.10054000
H	2.94321000	8.88657000	10.63571000
H	4.31601000	8.77944000	9.82021000
C	0.39600000	11.68019000	5.82350000
C	0.02677000	11.35080000	8.23916000
C	2.25416000	12.03825000	7.39030000
H	-0.26189000	12.28643000	8.28048000
H	0.44993000	11.10176000	9.08730000
H	-0.75128000	10.77579000	8.08148000
H	1.99017000	12.98163000	7.42828000
H	2.91572000	11.91252000	6.67893000
H	2.64420000	11.77411000	8.24999000
H	0.10761000	12.60897000	5.94499000
H	-0.38031000	11.12411000	5.60114000
H	1.05046000	11.63320000	5.09643000
C	3.86057000	8.57526000	-0.20395000
C	2.81294000	10.18931000	1.29809000
C	5.14028000	9.43423000	1.74595000
H	3.14049000	10.89600000	0.70312000
H	2.76502000	10.52963000	2.21506000
H	1.92067000	9.90575000	1.00726000
H	5.46054000	10.18961000	1.20946000
H	5.76637000	8.68566000	1.66307000
H	5.07639000	9.70526000	2.68560000
H	4.17347000	9.32801000	-0.74701000
H	2.97871000	8.28773000	-0.52031000
H	4.49367000	7.83052000	-0.28205000
C	5.11219000	1.08356000	0.40238000
C	3.43659000	-0.54985000	1.17131000
C	2.80133000	1.18106000	-0.48231000
H	3.06832000	0.57688000	-1.20720000
H	2.93641000	2.10930000	-0.76505000
H	1.85605000	1.03769000	-0.26954000
H	5.36338000	0.44138000	-0.29330000
H	5.66341000	0.93548000	1.19897000
H	5.25243000	1.99542000	0.07259000
H	3.69074000	-1.14301000	0.43337000
H	2.49262000	-0.68976000	1.39265000
H	3.98902000	-0.75216000	1.95630000
H	0.60209000	0.56657000	4.79782000
C	1.38194000	0.84114000	7.95666000
C	-0.69679000	-0.25955000	7.09127000
C	2.55170000	1.58684000	7.85469000
H	1.11344000	0.52978000	8.81322000
C	3.38252000	1.88916000	9.10455000
H	3.80611000	2.46762000	6.47349000
C	-1.53764000	0.45808000	8.12065000
C	-0.37355000	-1.65895000	7.58736000
C	-1.49965000	-0.39422000	5.78767000
H	-2.34213000	-0.86108000	5.97082000
H	-0.97821000	-0.90628000	5.13489000
H	-1.69076000	0.49635000	5.42832000
H	-1.21095000	-2.14317000	7.75863000
H	0.14394000	-1.60110000	8.41534000
H	0.14375000	-2.13499000	6.90667000
H	-2.41874000	0.03271000	8.17730000
H	-1.64449000	1.39667000	7.85905000
H	-1.09611000	0.41139000	8.99464000
C	3.75756000	0.62004000	9.81561000
C	4.65985000	2.61328000	8.75040000
C	2.54056000	2.73004000	10.03609000
H	4.25195000	0.83864000	10.63281000
H	4.31984000	0.07204000	9.22965000

H	2.94517000	0.12421000	10.04736000
H	3.07060000	2.97528000	10.82342000
H	1.75473000	2.21730000	10.31860000
H	2.25143000	3.54234000	9.57108000
H	5.19493000	2.74777000	9.56061000
H	4.44333000	3.48310000	8.35424000
H	5.17110000	2.07946000	8.10667000
H	-1.36679000	5.86930000	5.92123000
C	-1.47467000	6.80755000	5.65967000
C	-2.31509000	7.52552000	6.68915000
H	-0.59318000	7.23352000	5.60333000
H	-1.91582000	6.85494000	4.78580000
C	-3.60198000	6.73571000	6.92214000
C	-1.51199000	7.66001000	7.99283000
C	-2.63819000	8.92524000	6.19293000
C	-4.01087000	6.27846000	8.16610000
C	-4.39407000	6.42544000	5.82371000
H	-0.66938000	8.12745000	7.80948000
H	-2.03377000	8.17241000	8.64551000
H	-1.32130000	6.76976000	8.35198000
H	-1.80029000	9.40922000	6.02136000
H	-3.15553000	8.86707000	5.36532000
H	-3.15560000	9.40150000	6.87337000
N	-5.26968000	2.53134000	9.01795000
C	-5.54169000	4.96051000	9.62941000
C	-5.58828000	3.58002000	9.87080000
C	-5.15307000	5.51080000	8.30976000
C	-5.91216000	5.20221000	7.18104000
H	-3.61456000	6.69920000	8.98279000
H	-4.12589000	6.73644000	4.96666000
C	-5.56377000	5.67918000	5.92549000
C	-5.56576000	1.39149000	9.75895000
C	-5.86712000	5.82637000	10.65048000
C	-6.02470000	3.13720000	11.12905000
H	-6.81826000	4.79884000	7.30637000
C	-6.39420000	5.37686000	4.67566000
C	-5.49974000	0.03198000	9.38972000
C	-6.00907000	1.70832000	11.05589000
Si	-1.42257000	3.43312000	2.31342000
C	-6.28347000	5.39512000	11.90622000
H	-5.80308000	6.76021000	10.49153000
C	-6.36523000	4.02382000	12.12910000
C	-6.76935000	6.64542000	3.96496000
C	-5.55255000	4.53608000	3.74460000
C	-7.67178000	4.65263000	5.02976000
C	-5.88824000	-0.89931000	10.34183000
C	-5.16955000	-0.51570000	8.05159000
C	-6.34921000	0.73460000	11.99118000
C	-0.44662000	4.98041000	2.03503000
C	-3.01670000	3.54100000	1.40086000
C	-0.51407000	1.95134000	1.74822000
C	-6.66489000	6.37048000	13.02233000
H	-6.65703000	3.69842000	12.97355000
H	-7.26396000	6.42734000	3.14745000
H	-7.33153000	7.19463000	4.55036000
H	-5.95698000	7.14221000	3.73284000
H	-6.08270000	4.29135000	2.95653000
H	-4.76674000	5.04929000	3.46201000
H	-5.26316000	3.72451000	4.20889000
H	-8.20667000	4.51858000	4.21982000
H	-7.45505000	3.78362000	5.42592000
H	-8.18307000	5.18642000	5.67404000
H	-5.87526000	-1.81634000	10.09036000
C	-6.29190000	-0.59330000	11.63457000
C	-5.58116000	0.09994000	6.89260000
C	-4.59523000	-1.77745000	7.96244000
H	-6.61630000	0.98287000	12.86759000
H	-0.33819000	5.13011000	1.07285000
H	-0.92164000	5.74181000	2.43203000

H	0.43410000	4.89189000	2.45390000
H	-2.84458000	3.51770000	0.43700000
H	-3.58653000	2.78199000	1.64983000
H	-3.46941000	4.37814000	1.63301000
H	-0.42718000	1.97388000	0.77175000
H	0.37688000	1.93922000	2.15531000
H	-1.00562000	1.14654000	2.01317000
C	-5.81288000	6.08572000	14.26209000
C	-8.12353000	6.18243000	13.37812000
C	-6.44809000	7.81625000	12.60854000
C	-6.77467000	-1.73711000	12.53453000
H	-5.92858000	0.98437000	6.93045000
C	-5.50128000	-0.55102000	5.66380000
C	-4.50287000	-2.46316000	6.76729000
H	-4.25463000	-2.18075000	8.75054000
H	-6.08058000	6.68962000	14.98711000
H	-5.94845000	5.15728000	14.54554000
H	-4.86846000	6.22954000	14.04931000
H	-8.37541000	6.82503000	14.07370000
H	-8.67527000	6.33128000	12.58079000
H	-8.26451000	5.27150000	13.70783000
H	-6.70270000	8.40917000	13.34701000
H	-5.50448000	7.95650000	12.38734000
H	-7.00083000	8.01908000	11.82382000
C	-5.82493000	-2.92340000	12.48240000
C	-8.15222000	-2.16838000	12.03410000
C	-6.87247000	-1.30903000	13.98371000
C	-4.95831000	-1.81875000	5.63185000
C	-6.03694000	0.06958000	4.35425000
C	-4.03734000	-3.91079000	6.67814000
H	-6.15307000	-3.62922000	13.07705000
H	-5.77681000	-3.26310000	11.56479000
H	-4.93277000	-2.63915000	12.77258000
H	-8.47299000	-2.92287000	12.57072000
H	-8.77822000	-1.41921000	12.11738000
H	-8.08885000	-2.43871000	11.09463000
H	-7.18532000	-2.06170000	14.52706000
H	-5.99063000	-1.02091000	14.30081000
H	-7.50622000	-0.56338000	14.06194000
H	-4.89377000	-2.26646000	4.79667000
C	-7.12120000	1.11188000	4.63602000
C	-4.88550000	0.71294000	3.61832000
C	-6.65648000	-1.00998000	3.45347000
C	-3.03926000	-4.08398000	5.54102000
C	-5.26615000	-4.77162000	6.38966000
C	-3.40838000	-4.41355000	7.95659000
H	-7.47362000	1.45313000	3.78739000
H	-7.84607000	0.69781000	5.14909000
H	-6.73674000	1.85190000	5.15077000
H	-5.21664000	1.13773000	2.80081000
H	-4.46817000	1.38892000	4.19279000
H	-4.22354000	0.02772000	3.38704000
H	-7.08292000	-0.58450000	2.67970000
H	-5.95452000	-1.62068000	3.14481000
H	-7.32788000	-1.51280000	3.95981000
H	-2.74987000	-5.02009000	5.49982000
H	-3.46127000	-3.83609000	4.69323000
H	-2.26035000	-3.50921000	5.69831000
H	-5.00183000	-5.71542000	6.35183000
H	-5.92750000	-4.64642000	7.10122000
H	-5.65570000	-4.50756000	5.53010000
H	-3.11901000	-5.34274000	7.83548000
H	-2.63102000	-3.85799000	8.17864000
H	-4.06192000	-4.36684000	8.68412000

5 References

[1] A. Hinz, *Chem. Eur. J.* **2019**, *25*, 3267–3271.

[2] M. Westerhausen, *Inorg. Chem.* **1991**, *30*, 96-101.

[3] G. R. Fulmer, A. J. M. Miller, N. H. Sherden, H. E. Gottlieb, A. Nudelman, B. M. Stoltz, J. E. Bercaw, K. I. Goldberg, *Organometallics* **2010**, *29*, 2176–2179.

[4] L. van Gerven, J. Talpe, A. van Itterbeek, *Physica* **1967**, *33*, 207.

[5] G. M. Sheldrick, *Acta Crystallogr. Sect. A* **2008**, *64*, 112–122.

[6] G. M. Sheldrick, *Acta Crystallogr. A* **2015**, *71*, 3–8.

[7] G. M. Sheldrick, *Acta Crystallogr. C* **2015**, *71*, 3–8.

[8] C. B. Hübschle, G. M. Sheldrick, B. Dittrich, *J. Appl. Crystallogr.* **2011**, *44*, 1281–1284.

[9] M. J. Frisch, G. W. Trucks, H. B. Schlegel, G. E. Scuseria, M. A. Robb, J. R. Cheeseman, G. Scalmani, V. Barone, B. Mennucci, G. A. Petersson, H. Nakatsuji, M. Caricato, X. Li, H. P. Hratchian, A. F. Izmaylov, J. Bloino, G. Zheng, J. L. Sonnenberg, M. Hada, M. Ehara, K. Toyota, R. Fukuda, J. Hasegawa, M. Ishida, T. Nakajima, Y. Honda, O. Kitao, H. Nakai, T. Vreven, J. A. Montgomery, J. E. Peralta, F. Ogliaro, M. Bearpark, J. J. Heyd, E. Brothers, K. N. Kudin, V. N. Staroverov, R. Kobayashi, J. Normand, K. Raghavachari, A. Rendell, J. C. Burant, S. S. Iyengar, J. Tomasi, M. Cossi, N. Rega, J. M. Millam, M. Klene, J. E. Knox, J. B. Cross, V. Bakken, C. Adamo, J. Jaramillo, R. Gomperts, R. E. Stratmann, O. Yazyev, A. J. Austin, R. Cammi, C. Pomelli, J. W. Ochterski, R. L. Martin, K. Morokuma, V. G. Zakrzewski, G. A. Voth, P. Salvador, J. J. Dannenberg, S. Dapprich, A. D. Daniels, Ö. Farkas, J. B. Foresman, J. V. Ortiz, J. Cioslowski, D. J. Fox, *Gaussian 16, Revision B.01*, **2016**.
